# Immunomodulation by the combination of statin and matrix-bound nanovesicle enhances optic nerve regeneration

**DOI:** 10.1038/s41536-024-00374-y

**Published:** 2024-10-26

**Authors:** Gregory P. Campbell, Dwarkesh Amin, Kristin Hsieh, George S. Hussey, Anthony J. St. Leger, Jeffrey M. Gross, Stephen F. Badylak, Takaaki Kuwajima

**Affiliations:** 1 Department of Ophthalmology, University of Pittsburgh School of Medicine, Pittsburgh, PA 15219 USA; 2 The Louis J. Fox Center for Vision Restoration, University of Pittsburgh School of Medicine, Pittsburgh, PA 15219 USA; 3 Department of Pathology, University of Pittsburgh, Pittsburgh, PA 15219 USA; 4 McGowan Institute for Regenerative Medicine, University of Pittsburgh, Pittsburgh, PA 15219 USA; 5 Department of Bioengineering, University of Pittsburgh, Pittsburgh, PA 15219 USA; 6 Department of Immunology, University of Pittsburgh, Pittsburgh, PA 15213 USA; 7 Department of Developmental Biology, University of Pittsburgh School of Medicine, Pittsburgh, PA 15213 USA; 8 Department of Surgery, University of Pittsburgh, Pittsburgh, PA 15219 USA

**Keywords:** Regeneration and repair in the nervous system, Visual system

## Abstract

Modulating inflammation is critical to enhance nerve regeneration after injury. However, clinically applicable regenerative therapies that modulate inflammation have not yet been established. Here, we demonstrate synergistic effects of the combination of an HMG-CoA reductase inhibitor, statin/fluvastatin and critical components of the extracellular matrix, Matrix-Bound Nanovesicles (MBV) to enhance axon regeneration and neuroprotection after mouse optic nerve injury. Mechanistically, co-intravitreal injections of fluvastatin and MBV robustly promote infiltration of monocytes and neutrophils, which lead to RGC protection and axon regeneration. Furthermore, monocyte infiltration is triggered by elevated expression of CCL2, a chemokine, in the superficial layer of the retina after treatment with a combination of fluvastatin and MBV or IL-33, a cytokine contained within MBV. Finally, this therapy can be further combined with AAV-based gene therapy blocking anti-regenerative pathways in RGCs to extend regenerated axons. These data highlight novel molecular insights into the development of immunomodulatory regenerative therapy.

## Introduction

CNS neurons in humans have little or no capacity for axon regeneration after injury or as a result of neurodegenerative disease. Optic nerve damage resulting from trauma and in optic neuropathies causes the death of retinal ganglion cells (RGCs), ultimately leading to vision loss. A major barrier in the field is the lack of clinically viable therapies that can be applied after injury to mitigate RGC death and optic nerve degeneration or stimulate RGC axon regeneration. Ideally, the lag time between nerve injury and the first treatment should be kept to a few days as the survival rate for RGCs after injury diminishes dramatically thereafter. Following optic nerve crush (ONC) in mice, a preclinical mouse model for optic neuropathy, RGCs begin to die within three days and ~60% of RGCs die within five to seven days^1,2^. Thus, there is an urgent need for effective neuroprotective and regenerative therapies that can be applied shortly after acute trauma and neuropathies.

In previous work, we tested >50,000 small molecules for their ability to promote axon outgrowth on inhibitory substrata in vitro. Of these, statins were identified as the most potent candidates^3^. Statins are inhibitors of 3-hydroxy-3-methylglutaryl-CoA (HMG-CoA) reductase and are widely prescribed for treatment of hypercholesterolemia. Among the nine types of statins, cerivastatin was the most effective in stimulating axon outgrowth, even in the in vitro growth-inhibitory environment, and a single intravitreal injection of cerivastatin significantly enhanced retinal axon regeneration after ONC in mouse^3^. However, cerivastatin has been withdrawn from the market because of associated rhabdomyolysis^4^. Fluvastatin was the second most effective statin for stimulating axon outgrowth in vitro^3^ and remains clinically available.

Statins directly affect neurons to enhance axon regeneration by inhibiting protein prenylation^3,5^ and other pathways^6^. However, emerging evidence indicates that statins also play significant roles as inhibitors of inflammation to protect neurons after traumatic brain injury^7,8^ and spinal cord injury^9^ and as enhancers of inflammation in other contexts^10^. Thus, statin-mediated cell- and non-cell-autonomous mechanisms regulate neuroprotection, axon regeneration and other cellular functions.

Extracellular matrix (ECM)-based biological scaffold materials have been broadly applied for regenerative medicine in humans^11^. The ECM is a complex structure containing more than 300 proteins, many of which favorably affect the immune response^12^. Our previous studies have identified matrix-bound nanovesicles (MBV) as a potent functional component of the ECM^13^. MBV are nanometer-sized lipid-bound vesicles, well-tolerated, and not toxic to the whole organism^14^. Intriguingly, MBV contain cytokines such as IL-33 and microRNAs (miRNAs) that target genes relevant to immune responses^15–18^. Indeed, MBV elicit crucial immunomodulatory functions in several rodent disease models^19,20^. In particular, in an ocular hypertension model, MBV exert RGC protective effects by immunomodulation and inhibiting the secretion of neurotoxic cytokines in microglia and astrocytes^21^. Thus, MBV may serve as functional modulators of inflammation for neuroprotection and other cellular context in diseases.

There is increasing evidence suggesting that immune cells typically associated with inflammation can contribute to optic nerve regeneration and neuroprotection. For instance, lens injury promotes RGC survival and axon regeneration after optic nerve injury by enhancing infiltration of macrophages and others^22–24^. Intravitreal injections of zymosan, a yeast cell wall extract, also stimulates infiltration of several types of immune cells to the retina and facilitates neuroprotection and axon regeneration^25^. Indeed, modulation of inflammatory responses after nerve injury has become a targeted strategy to stimulate axon regeneration and neuroprotection in the visual system as well as many other central nervous system (CNS) tissues^26^. Thus, identification of novel, effective and clinically tractable molecular interventions that modulate inflammation can be broadly beneficial for CNS axon regeneration.

Here we demonstrate that co-intravitreal injections of statin/fluvastatin and MBV robustly enhance optic nerve regeneration and RGC protection after optic nerve crush (ONC) in mice. The mechanisms by which fluvastatin and MBV act were revealed by bulk RNA sequencing and flow cytometric analysis that as a result of infiltration of monocytes and neutrophils, RGC protection and axonal regeneration were enhanced. Furthermore, the combination of fluvastatin and MBV or IL-33, an MBV-associated cytokine regulates monocyte infiltration via elevation of CCL2 expression in the superficial layer of the retina. We also demonstrate that combinatorial treatments using fluvastatin, MBV and adeno-associated virus (AAV)-based gene therapy blocking anti-regenerative protein prenylation pathways in RGCs enabled regenerated axons to extend longer. Taken together, this study provides novel molecular insights into enhanced axon regeneration and neuroprotection, leading to a significant advance in identification of the therapeutic intervention.

## Results

### Synergistic effects of fluvastatin and MBV on optic nerve regeneration and RGC protection after ONC

**Fig. 1:**
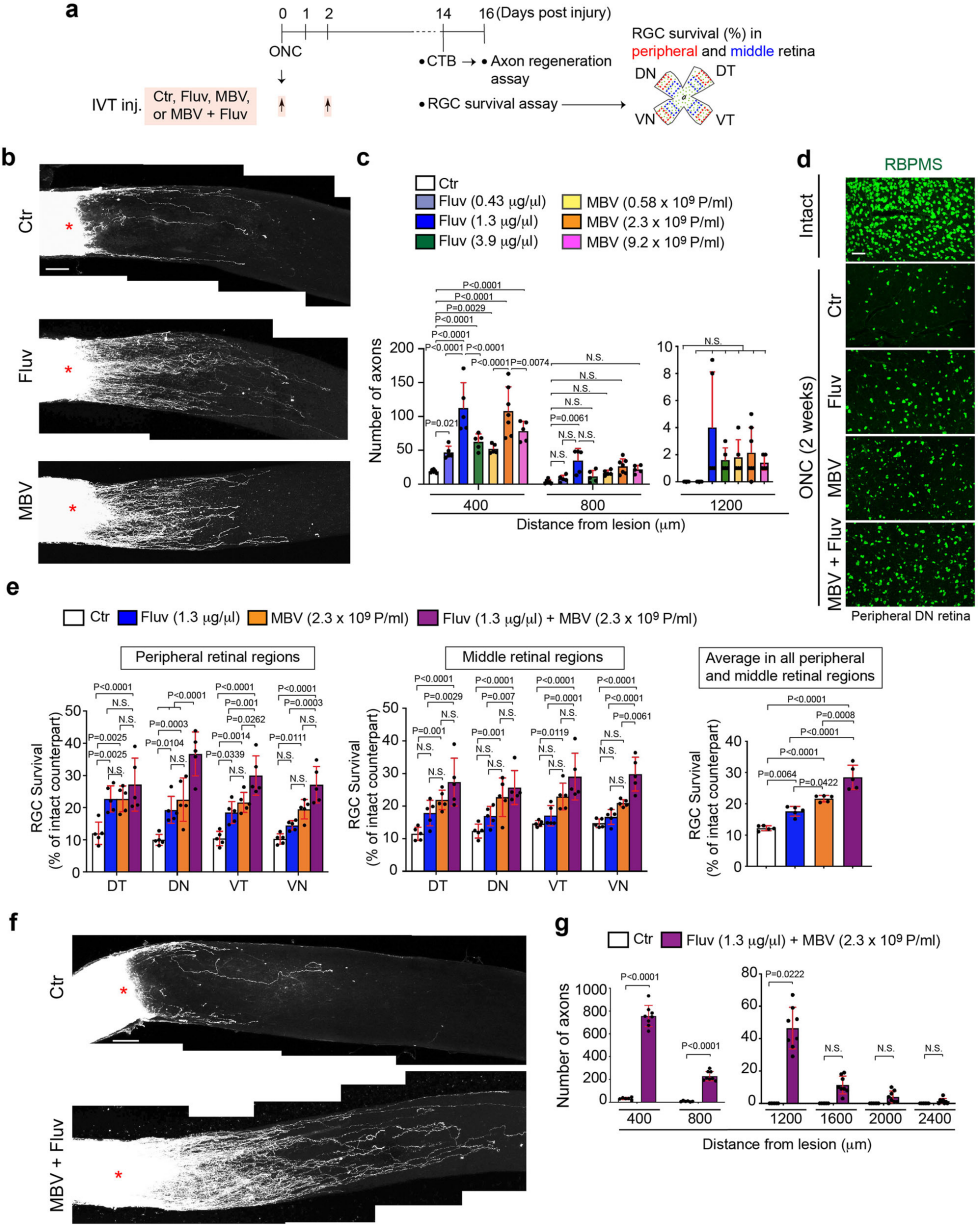
Fluvastatin and MBV synergistically promote optic nerve regeneration and RGC protection after ONC. **a** Schema of intravitreal injections (IVT. Inj.) of PBS (Ctr), fluvastatin, MBV or the combination of MBV and fluvastatin after optic nerve crush (ONC) and analysis of CTB^+^ retinal axon regeneration and RBPMS^+^ RGC survival analysis in four quadrants of the peripheral and middle retina two weeks after ONC. **b** Images of CTB^+^ axon regeneration from the retina treated with PBS (Ctr), 1.3 μg/μl fluvastatin, or 2.3 × 10^9^ particles/ml (P/ml) MBV two weeks after ONC. **c** Quantitative analysis of the number of regenerated axons at 400, 800 and 1200 μm from the lesion site in each condition two weeks after ONC. (*n* = 5–7, two-way ANOVA with Tukey’s post-hoc). **d** Images of RBPMS^+^ RGCs in the intact retina or the injured retina treated with PBS (Ctr), 1.3 μg/μl fluvastatin, 2.3 × 10^9^ particles/ml (P/ml), or the combination of MBV and fluvastatin two weeks after ONC. **e** Quantitative analysis of RBPMS^+^ RGC survival (%) in each quadrant of the peripheral and middle retina or the average of RGC survival from all four regions in the retina treated with PBS (Ctr), fluvastatin, MBV, or the combination of fluvastatin and MBV two weeks after ONC. (*n* = 5, two-way ANOVA with Tukey’s post-hoc for RGC survival in each region of the peripheral and middle retina; one-way ANOVA with Tukey’s post-hoc for average RGC survival). **f** Images of CTB^+^ axon regeneration from the retina treated with PBS (Ctr) or the combination of fluvastatin and MBV two weeks after ONC. **g** Quantitative analysis of the number of regenerated axons at 400–2400 μm from the lesion sites in each condition two weeks after ONC. (*n* = 7–8, two-way ANOVA with Bonferroni’s test). Lesion sites marked by asterisks (**b** and **f**). Data presented as mean ± SD. N.S. not significant; Scale bars represent 100 μm (**b**, **d** and **f**).

In this study, first we investigated whether intravitreal injections of fluvastatin promote retinal axon regeneration after ONC in 8-week old wild-type (WT) C57BL/6J mice (Fig. 1a–c). Fluvastatin requires a~100-fold greater concentration than cerivastatin to enhance axon outgrowth to the same extent in vitro^3^. We therefore tested 2 μl of 0.43, 1.3 and 3.9 μg/μl fluvastatin or PBS as a negative control. We also increased the frequency of injections from once to twice, administered a few minutes and two days after ONC (Fig. 1a). Repeated intravitreal injections potentially cause damage to the lens of the ONC-injured eye that augments the inflammatory responses by activated macrophages^23^. Thus, to test whether intravitreal injections affect inflammation after ONC, we intravitreally injected PBS twice after ONC and analyzed the number of macrophages (F4/80^+^ cells) three days after ONC. We found that the number of F4/80^+^ cells in the ONC-injured retina treated with PBS is comparable to that in the ONC-injured retina without any injections. Thus, at least intravitreal injections twice after ONC led to no increase in macrophages (Supplementary Fig. 1a, b). We quantified the number of regenerated axons from the lesion site using the anterograde axonal tracer Alexa Fluor 555-conjugated cholera toxin subunit B (CTB) two weeks after ONC. Among three different concentrations, 1.3 μg/μl fluvastatin showed the greatest number of regenerated axons at 400 μm from the lesion site and regenerated axons extended up to 800 μm from the lesion site (Fig. 1c).

It is known that RGC death and neurodegeneration do not occur evenly across the retina in optic neuropathies^27^, and our previous work demonstrated molecular mechanisms underlying ventral-region specific RGC death^1^. With this heterogeneous distribution, we quantified RGC survival in four quadrants: ventrotemporal (VT), ventronasal (VN), dorsotemporal (DT) and dorsonasal (DN) of the peripheral and middle retina (400 μm × 600 μm area) two weeks after ONC. We found 1.3 μg/μl fluvastatin showed two-fold increase in RGC survival when compared to PBS in the peripheral DT, DN and VT retina, while it had no neuroprotective effects in peripheral VN retina or any regions of the middle retina (Fig. 1d, e). The average of RGC survival rates from all retinal regions showed a 1.5-fold increase in RGC survival for fluvastatin when compared to PBS. Thus, fluvastatin promoted optic nerve regeneration and showed RGC protective effects in the peripheral retina.

We next aimed to identify complementary RGC protective agents to increase the number of surviving RGCs treated with fluvastatin after ONC. We focused on matrix-bound nanovesicles (MBV) as a neuroprotective agent because MBV, which were prepared from porcine urinary bladders, inhibit RGC death in an ocular hypertension rat model, and MBV also stimulate RGC axon outgrowth in vitro^21^. In this study, we also used MBV derived from the same source, porcine urinary bladders, and we examined whether intravitreal injections of MBV a few minutes and two days after ONC promote axon regeneration and RGC protection after ONC (Fig. 1a). To minimize batch-to-batch variations, quality control was performed to ensure the size and concentrations of particles by Nanosight nanoparticle tracking analysis and imaging with transmission electron microscopy (TEM) (Supplementary Fig. 2a, b). Since intravitreal injections of 1 μl of 5 μg/ml of MBV attenuated RGC death in an ocular hypertension rat model^21^, we tested 2 μl of 5, 20 and 80 μg/ml (0.58 ×10^9^, 2.3 ×10^9^ and 9.2 ×10^9^ particles/ml, respectively) of MBV (Fig. 1a). We found that 2.3 ×10^9^ particles/ml of MBV more robustly enhanced axon regeneration than other concentrations, and regenerated axons extended up to 400 μm from the lesion site (Fig. 1b, c). Next, we investigated the effects of 2.3 ×10^9^ particles/ml MBV on RGC neuroprotection two weeks after ONC (Fig. 1d, e). MBV protected RGCs in all peripheral and middle retinal regions except for middle VN regions, when compared to PBS controls (Fig. 1e). These data confirm that MBV show protective effects on both optic nerve regeneration and RGC protection after ONC.

We next assessed the effects of combining 1.3 μg/μl for fluvastatin and 2.3 ×10^9^ particles/ml for MBV on RGC protection and optic nerve regeneration two weeks after ONC following intravitreal injections of fluvastatin and MBV together a few minutes and two days after ONC (Fig. 1a). The combined treatment also enhanced RGC survival in all peripheral and middle retinal regions when compared to PBS (Fig. 1d, e). Indeed, based on the average RGC survival rate in all retinal regions, the combined treatment yielded higher neuroprotective outcomes when compared to either fluvastatin or MBV alone (Fig. 1e). Furthermore, the combined treatment more robustly promoted axon regeneration when compared to PBS, fluvastatin or MBV alone (Fig. 1f, g). For example, at 400 μm from the lesion site, 754 regenerated axons were detected in the injured nerve treated with the combination of fluvastatin and MBV, while 112 or 108 regenerated axons were detected in the injured nerve treated with either fluvastatin or MBV alone, respectively (Fig. 1c, g). Furthermore, regenerated axons after the combined treatment were detected up to 1200 μm from the lesion site, while fluvastatin or MBV alone extended regenerated axons only to 800 or 400 μm, respectively. We also validated the neuroprotective and regenerative effects of different batches of MBV with fluvastatin and confirmed the similar outcomes (Supplementary Fig. 3a, b). Thus, MBV and fluvastatin synergistically enhance axon regeneration and RGC protection pan-retinally.

### Identification of differentially expressed genes by MBV and fluvastatin after ONC

**Fig. 2:**
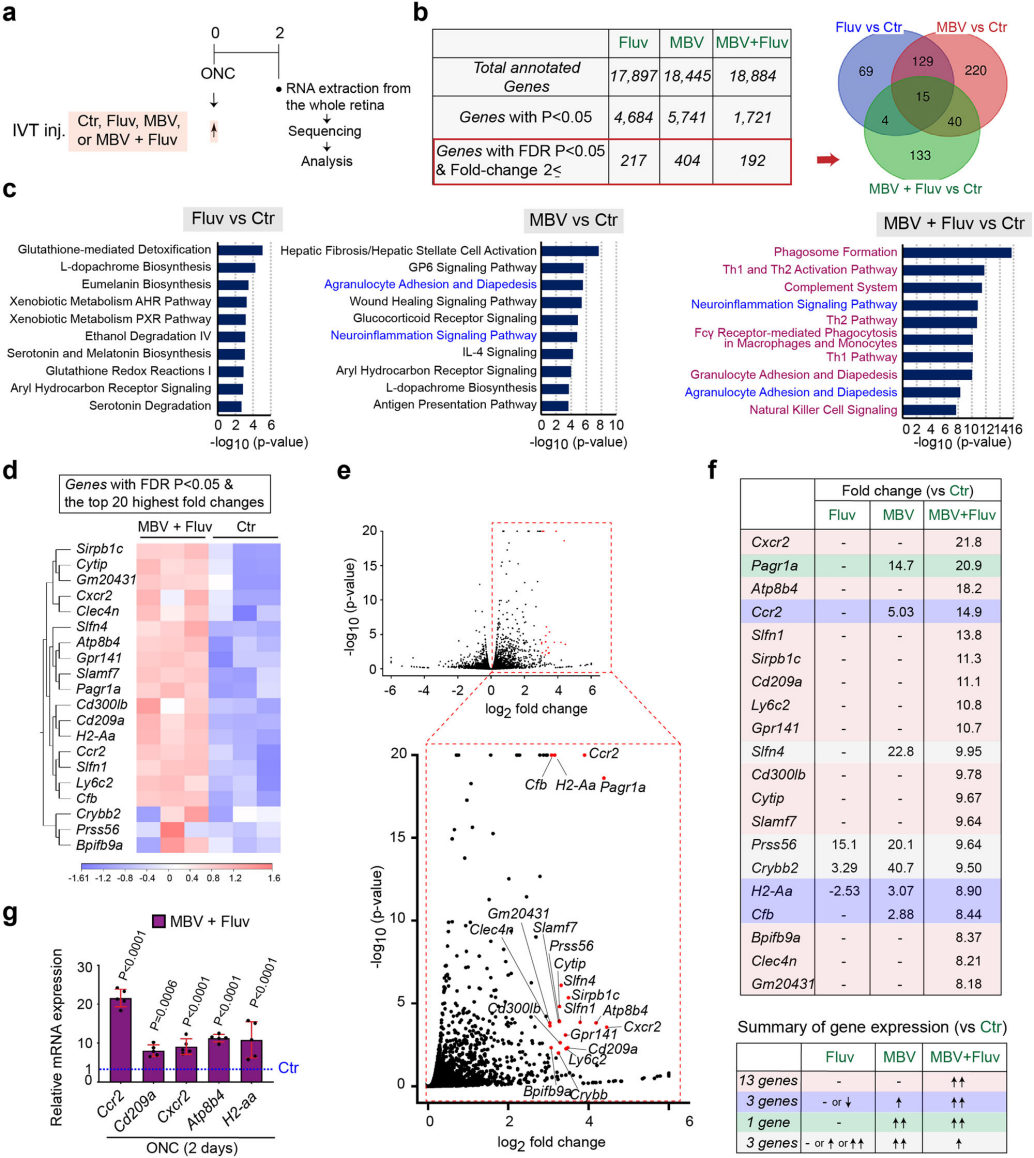
Transcriptome profiling of the retina with MBV and fluvastatin after ONC. **a** Schema of intravitreal injections (IVT inj.), RNA preparations and bulk RNA sequencing for transcriptome profiling in the whole retina two days after ONC. **b** The number of total annotated genes, genes with *P*-value < 0.05, and genes with an FDR *p*-value < 0.05 and a fold-change of at least 2 (referred to as top DEGs) in each treatment compared to PBS (Ctr). A Venn Diagram describes the similarities and differences of the top DEGs in the retina treated with the single or combined treatments. **c** Enriched cellular processes and pathways based on the top DEGs by each treatment. Bars indicate the degree of -log_10_ *P* value. **d**, **e** Heatmap (**d**) and volcano plots in red (**e**) showing 20 DEGs with the highest fold-changes and an FDR *p*-value < 0.05 (referred to as the top 20 DEGs) by the combined treatment compared to PBS (Ctr). In the heatmap (**d**), color values indicate log2-transformed expression values shown in the color key (bottom) relative to the average expression in the retina with PBS (Ctr). **f** The magnitude of expression levels of the top 20 DEGs in the retina treated with the combined treatment and analysis of expression levels of those DEGs in the retina treated with the single treatments. **g** Validation of expression of some of the top DEGs by quantitative RT-PCR in the retina treated with the combined treatment compared to PBS (Ctr) two days after ONC (*n* = 5, one-way ANOVA with Tukey’s post-hoc, Data presented as mean ± SD).

We next aimed to identify the molecular mechanisms underlying robust enhancement of axon regeneration and RGC neuroprotection by the combination therapy. Within three days, RGCs begin to die^1,2^. Prior to that, within two days after optic nerve crush or nerve transection, expression of intrinsic and extrinsic molecules relevant to RGC protection and/or axon regeneration is significantly altered^28–30^. Thus, we collected the whole retina at two days after ONC following intravitreal injections of either MBV or fluvastatin alone, the combination of MBV and fluvastatin, or PBS as a negative control a few minutes after ONC, and we identified differentially expressed genes (DEGs) by bulk RNA sequencing (RNA-seq) (Fig. 2a). DEGs with *p*-value < 0.05 revealed 4684, 5741 or 1721 DEGs in the injured retina treated with either fluvastatin or MBV alone, or the combination of MBV and fluvastatin, respectively, when compared to PBS (Fig. 2b). Among them, genes altered by a combination of MBV and fluvastatin include previously identified genes which are expressed in RGCs that mediate axon regeneration such as *Rab34*, *Rab27b*^31^, *Gal*, *Crh*^32^ and *Gap43*^33^. Next, we identified DEGs with an FDR *p*-value < 0.05 and a fold-change of at least 2 (referred to as top DEGs) when compared to PBS (Fig. 2b and Supplementary Tables 1–4). Among the top 192 DEGs induced by the combination of MBV and fluvastatin, 133 (69.3%) appeared only when MBV and fluvastatin were co-injected (Fig. 2b). Ingenuity pathway analysis identified the top 10 enriched cellular processes in each treatment (Fig. 2c). The combination of MBV and fluvastatin broadly influenced immune cell-related pathways such as phagosome formation, Th1 and Th2 activation, complement, neuroinflammation signaling and granulocyte adhesion and diapedesis, some of which are known to be involved in RGC survival/death and optic nerve regeneration^25,26^. In contrast, fluvastatin had no effects on immune responses. MBV are involved in regulation of both non-immune cell-mediated pathways and immune cell-mediated pathways such as agranulocyte adhesion and diapedesis, and neuroinflammation signaling.

Among the top DEGs, we then focused on the 20 DEGs with the highest fold-changes and an FDR p-value < 0.05 (referred to as the top 20 DEGs) in the injured retina with combined treatment when compared to PBS (Fig. 2d–g). These top 20 DEGs were all up-regulated by the combined treatment, and most of them showed an increase in expression levels in all replicates (Fig. 2d, e). Next, we compared fold-changes of these top 20 DEGs to those from the single treatments (Fig. 2f). 13 DEGs were upregulated only when MBV and fluvastatin were co-injected. Three DEGs were upregulated by MBV alone as well; however, fold-changes by the combined treatment were more robust than those elicited by MBV alone. The remaining four DEGs were unchanged or downregulated by the combined treatment when compared to MBV or fluvastatin alone. These top 20 DEGs include *Ccr2*^34^, *Sirpb1c*^35^, *Cd209a*^36^, *Ly6C2*^37^, and *Slamf7*^38^, which are normally expressed in monocytes, and *Cxcr2*^34^, *Atp8b4*^39^, *H2-aa*^40^ and *Clec4n*^41^, which are expressed in neutrophils. Among those DEGs, further analysis using qPCR confirmed that genes expressed in monocytes (*Ccr2* and *Cd209a*) and genes expressed in neutrophils (*Cxcr2*, *Atp8b4*, and *H2-aa*) are significantly upregulated by the combination of MBV and fluvastatin compared to PBS two days after ONC (Fig. 2g). Thus, the combination of MBV and fluvastatin more significantly stimulated gene expression related to immune responses compared to either MBV or fluvastatin alone after ONC.

### Neutrophil and monocyte infiltration is enhanced by MBV and fluvastatin

**Fig. 3:**
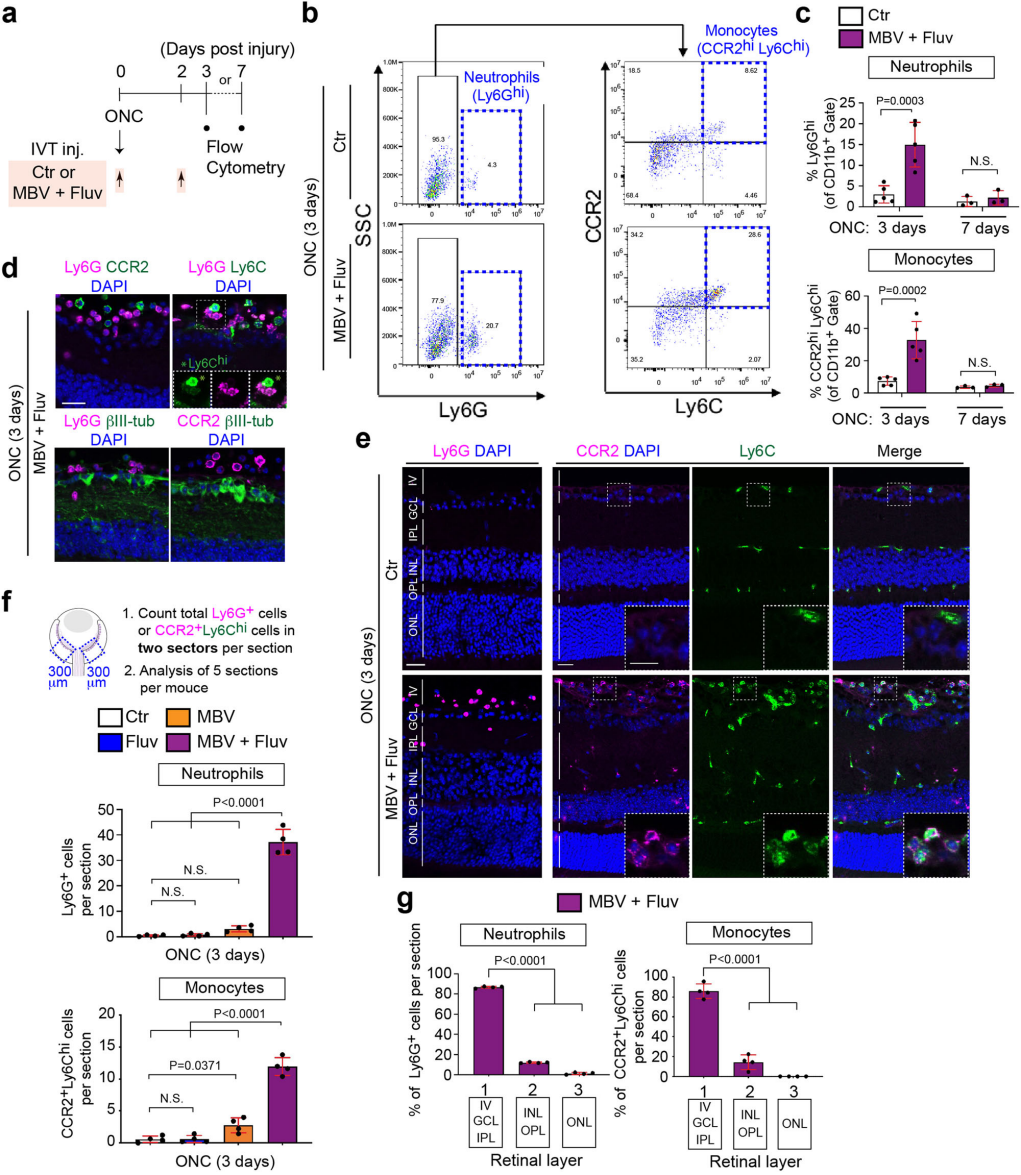
Neutrophils and monocytes are robustly infiltrated by the combination of MBV and fluvastatin after ONC. **a** Schema of intravitreal injections (IVT inj.) and preparations of the injured retina for flow cytometric analysis. **b** Representative flow cytometry dot plots of neutrophils (Ly6G^hi^) against side scatter (SSC), and monocytes (CCR2^hi^Ly6C^hi^) after gating on Ly6G-negative cells in the retina treated with PBS (Ctr) or the combination of MBV and fluvastatin three days after ONC. **c** Quantification of percentages of neutrophils (Ly6G^hi^) and monocytes (CCR2^hi^Ly6C^hi^) among the CD11b^+^ myeloid cells in the retina treated with PBS (Ctr) or the combination of MBV and fluvastatin three or seven days after ONC. (*n* = 3–5, two-way ANOVA with Bonferroni’s test). **d** Images of neutrophils (Ly6G^+^), monocytes (Ly6C^hi^ (*), CCR2^+^) and RGCs (βIII-tubulin^+^) in the retina treated with the combined treatment three days after ONC. **e** Images of neutrophils (Ly6G^+^) and monocytes (CCR2^+^Ly6C^hi^) in the retina treated with the combination of MBV and fluvastatin or PBS (Ctr) three days after ONC. **f**, **g** Quantitative analysis of the number of neutrophils (Ly6G^+^) and monocytes (CCR2^+^Ly6C^hi^) (**f**) and localization (**g**) in the retina treated with PBS (Ctr), fluvastatin, MBV and the combination of MBV and fluvastatin three days after ONC. Total number of those cells from two 300 μm (width) retinal areas adjacent to the nerve head per section were provided, and the average from five sections per animal was calculated (**f**). Quantitative analysis of those immune cell the localization within the retina in three layers, 1: Intravitreal (IV), Ganglion cell layer (GCL) and Inner plexiform layer (IPL), 2: Inner nuclear layer (INL) and Outer plexiform layer (OPL), 3: Outer nuclear layer (ONL) and Outer Limiting Membrane (OLM) (**g**). (*n* = 4, one-way ANOVA with Tukey’s post-hoc). Data presented as mean ± SD. N.S. not significant; Scale bars represent 20 μm.

Since the genes indicative of monocytes and neutrophils are upregulated by the combination of MBV and fluvastatin two days after ONC, we hypothesized that the combine treatment could enhance infiltration of monocytes and neutrophils within a few days after ONC (Fig. 3). To test the hypothesis, we quantified monocyte and neutrophil infiltration efficacy by flow cytometry at three days and at the later time point to define the duration of the effectiveness, seven days after ONC following intravitreal injections of MBV and fluvastatin or PBS as previously analyzed for neuroprotection and axon regeneration (Fig. 3a). Using Ly6G (Ly6G^hi^) expression in CD11b^+^ cells, we were able to distinguish neutrophils from other myeloid-derived cells^42^ (Fig. 3b). Significantly more neutrophils infiltrated the retina in mice treated with the combined treatment compared to mice treated with PBS three days after ONC (Fig. 3c). As cells highly expressing Ly6C (Ly6C^hi^) were plotted and defined as monocytes^42,43^, we also confirmed that infiltration of CCR2^+^ Ly6C^+^ monocytes was greater in the retina treated with the combined treatment compared to PBS three days after ONC (Fig. 3b, c). In contrast, at seven days after ONC, the combined treatment had no impact on enhancement of infiltration of neutrophils and monocytes within the retina. Thus, the combined treatment transiently stimulates infiltration of neutrophils and monocytes after ONC.

Next, we reexamined these effects using cryosections of the injured retina three days after ONC. We first confirmed that neutrophils (Ly6G^+^ cells), monocytes (CCR2^+^ Ly6C^hi^ cells) and RGCs (βIII-tubulin^+^ cells) were easily distinguishable in the injured retina treated with the combined treatment (Fig. 3d). We then compared the magnitude of infiltration of those immune cells by either fluvastatin or MBV alone to the combined treatment three days after ONC (Fig. 3e, f). We found that the combined treatment, but not fluvastatin or MBV alone, enhanced neutrophil infiltration. Furthermore, fluvastatin had no effect on infiltration of monocytes, and MBV showed a slightly increased number of monocytes. However, the combined treatment was more effective in stimulating monocyte infiltration than MBV alone. We then examined the localization of these infiltrating immune cells by the combined treatment (Fig. 3g). ~80% of infiltrating neutrophils and monocytes were located within the ganglion cell layer (GCL), the inner plexiform layer (IPL) and in the intravitreal area within 100 μm above the GCL. Thus, the combination of MBV and fluvastatin more robustly facilitated infiltration of neutrophils and monocytes than either MBV or fluvastatin after ONC.

### Neutrophils and monocytes infiltrated by MBV and fluvastatin regulate axon regeneration and neuroprotection

**Fig. 4:**
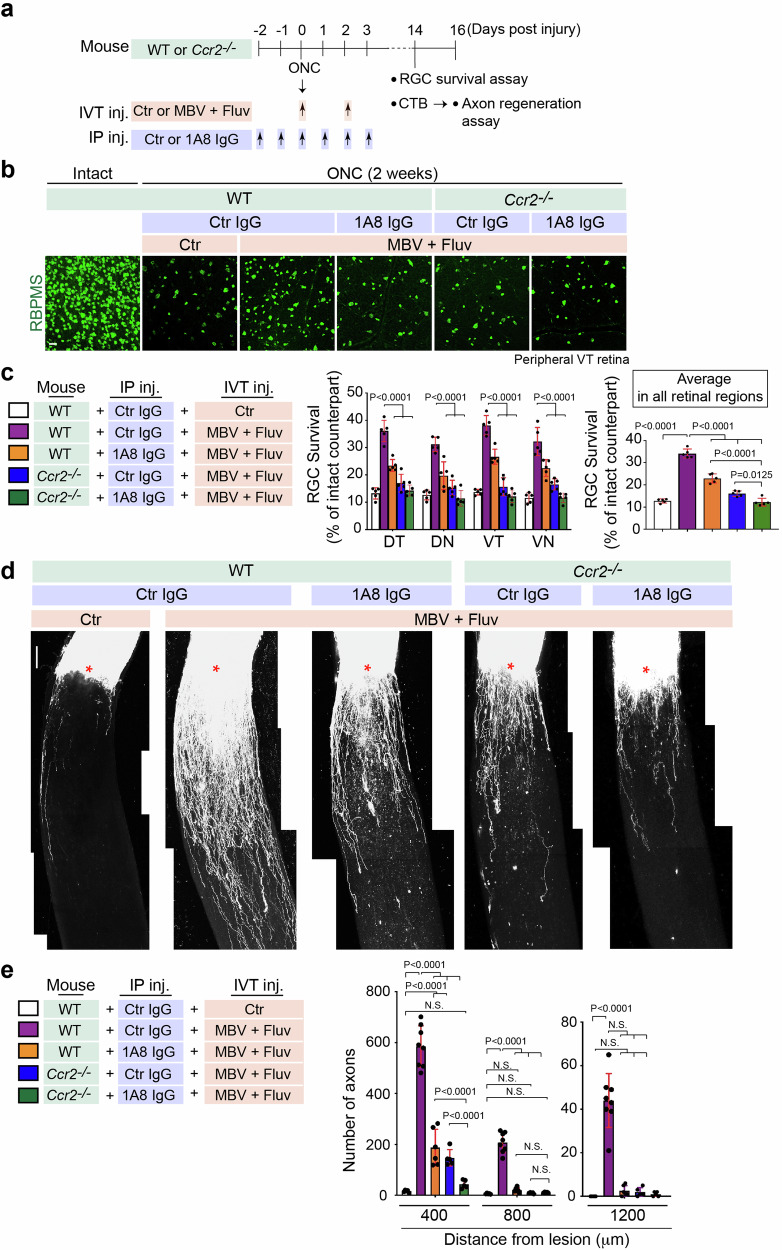
Axon regeneration and neuroprotection are mediated by neutrophils and monocytes infiltrated by MBV and fluvastatin after ONC. **a** Schema of RBPMS^+^ RGC survival analysis and axon regeneration after ONC in WT or *Ccr2*^*−/−*^ mouse with intravitreal injections (IVT inj.) of PBS (Ctr) or the combination of MBV and fluvastatin post-injury and multiple intraperitoneal injections (IP inj.) of 1A8 or control IgG2a (Ctr) antibody pre- and post-injury. **b** Images of RBPMS^+^ RGCs in the intact retina or the injured retina in each condition two weeks after ONC. **c** Quantitative analysis of RBPMS^+^ RGC survival (%) in each quadrant of the peripheral region or the average of RGC survival in all four regions of the peripheral retina in each condition two weeks after ONC. (*n* = 5, two-way ANOVA with Tukey’s post-hoc for RGC survival in each region of the peripheral retina; one-way ANOVA with Tukey’s post-hoc for average RGC survival). **d** Images of CTB^+^ regenerated axons in each condition two weeks after ONC. Lesion sites marked by asterisks. **e** Quantitative analysis of the number of regenerated axons at 400–1200 μm from the lesion sites in each condition two weeks after ONC. (*n* = 5–8, two-way ANOVA with Tukey’s post-hoc). Data presented as mean ± SD. N.S. not significant; Scale bars represent 20 μm (**b**) and 100 μm (**d**).

We hypothesized that RGC neuroprotection and axon regeneration by the combined treatment could be regulated by infiltrating neutrophils and monocytes. Therefore, we investigated whether depleting these immune cells dampened RGC survival and optic nerve regeneration even though MBV and fluvastatin were co-injected (Fig. 4). To deplete neutrophils, we used the anti-mouse Ly6G 1A8 clone antibody (hereafter referred to as 1A8) which has been previously utilized in the retina and other tissues^42,44^. We intraperitoneally injected 1A8 or an isotype-matched IgG2a antibody as a negative control at five intervals: two days and one day before ONC, and a few minutes, one, two and three days after ONC (Fig. 4a). To deplete monocytes, we used the *Ccr2*^*−/−*^ mouse^45^, which results in a significant reduction in monocyte recruitment^43^. To deplete both immune cell types, we intraperitoneally injected 1A8 into the *Ccr2*^*−/−*^ mouse, as above.

We confirmed that neutrophils (Ly6G^+^ cells) and/or monocytes (Ly6C^hi^ cells) were absent after immune cell depletion even though fluvastatin and MBV were co-injected (Supplementary Fig. 4). We next determined whether increased RGC survival by fluvastatin and MBV after ONC was dependent on infiltrating neutrophils and/or monocytes (Fig. 4b, c). Depletion of either neutrophils or monocytes alone, or both cell types, led to a reduction in RGC survival in all peripheral retinal quadrants, DT, DN, VT and VN, even though MBV and fluvastatin were co-injected. Furthermore, given the average RGC survival rate from all peripheral retinal regions, depletion of both neutrophils and monocytes resulted in a more significant reduction in RGC survival when compared to depletion of either neutrophils or monocytes alone. We then investigated whether neutrophils and monocytes contributed to optic nerve regeneration two weeks after ONC. When either neutrophils or monocytes were depleted, axon regeneration efficacy (number of regenerated axons from the lesion site) was attenuated. Depletion of both neutrophils and monocytes led to minimal axonal regeneration outcomes even though MBV and fluvastatin were co-injected (Fig. 4d, e). These data support a model in which infiltrating neutrophils and monocytes, stimulated by fluvastatin and MBV, are required for RGC protection and axon regeneration after ONC.

### Elevated CCL2 expression in the superficial layer of the retina treated with MBV and fluvastatin directs monocyte infiltration

**Fig. 5:**
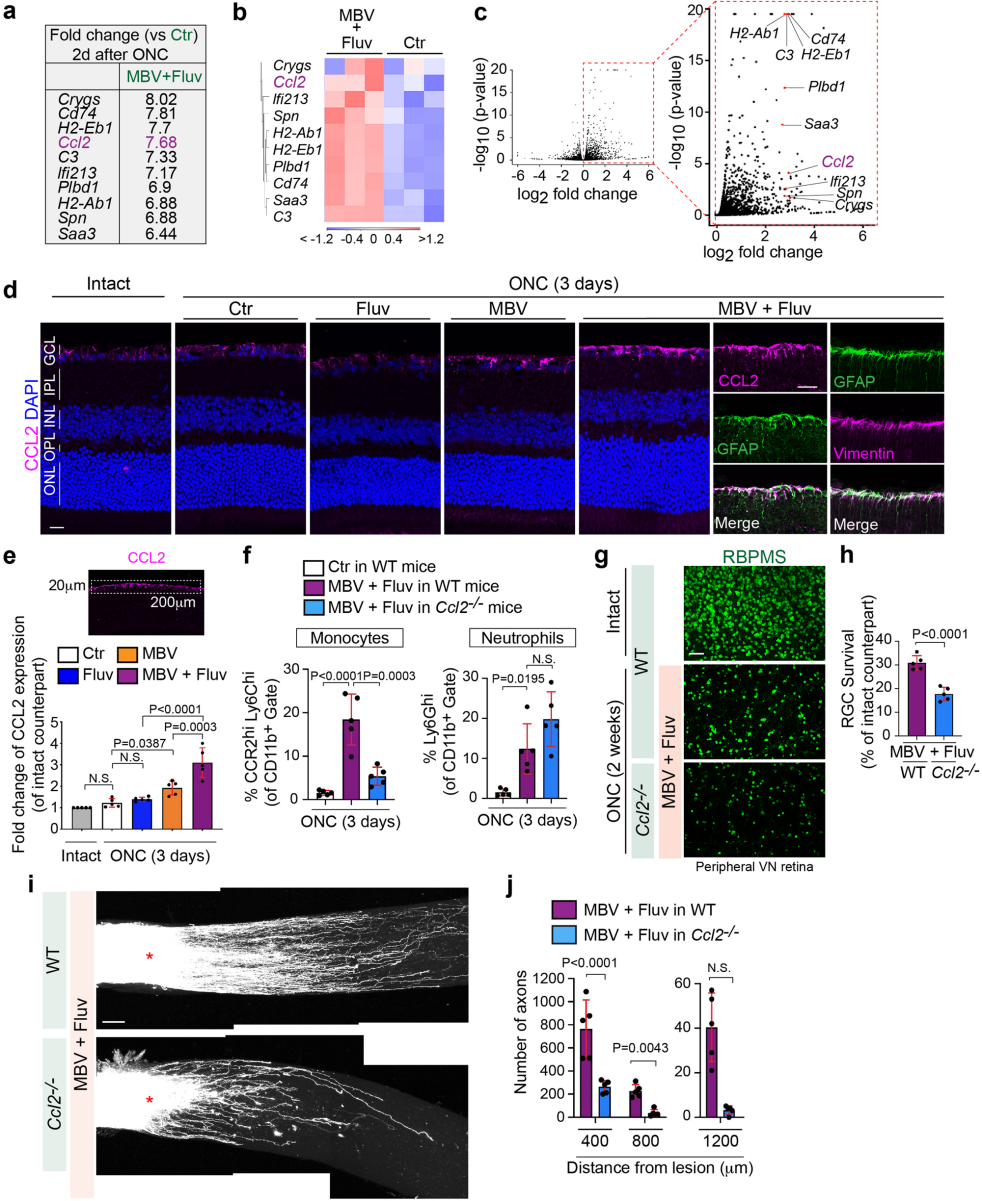
CCL2, a chemokine for monocyte infiltration is upregulated in the superficial retinal layer by the combination of fluvastatin and MBV after ONC. **a**–**c** The list showing DEGs with the 21^st^ - 30^th^ highest fold-changes based on bulk RNA-seq shown in Fig. 2a includes *Ccl2*. Heatmap (**b**) and volcano plots in red (**c**) showing *Ccl2* and other top DEGs listed in **a** (*n* = 3). In the heatmap (**b**), color values indicate log2-transformed expression values shown in the color key (bottom) relative to the average expression in Ctr. **d** Expression of CCL2 protein in the intact and injured retina with the single and combined treatments three days after ONC. Note that CCL2 is highly upregulated by the combined treatment in Müller glia (GFAP^+^ and vimentin^+^). **e** Quantitative analysis of fold-change of CCL2 expression in the injured retina with the single and combined treatments three days after ONC compared to the intact retina based on the mean pixel intensity of CCL2^+^ retinal surface area (200 μm width × 20 μm depth). (*n* = 5, one-way ANOVA with Tukey’s post-hoc). **f** Quantification of percentages of neutrophils (Ly6G^hi^) and monocytes (CCR2^hi^Ly6C^hi^) among the CD11b^+^ myeloid cells in the retina treated with PBS (Ctr) or the combination of MBV and fluvastatin three days after ONC in WT mice or *Ccl2*^*−/−*^ mice. (*n* = 5, one-way ANOVA with Tukey’s post-hoc). **g**, **h** Images of RBPMS^+^ RGCs in the intact retina or the injured retina in each condition two weeks after ONC (**g**) and quantitative analysis of RGC survival (%) based on the average of RGC survival in all four regions of the peripheral retina (**h**). (*n* = 5, two-tailed unpaired t-test). **i**, **j** Reduced axon regeneration by the combination of MBV and fluvastatin in *Ccl2*^*−/−*^ mouse compared to WT mice two weeks after ONC (**i**), and quantitative analysis of the number of regenerated axons at 400–1200 μm from the lesion site (**j**). (*n* = 5, two-way ANOVA with Bonferroni’s test). Data presented as mean ± SD. N.S. not significant; Scale bars represent 20 μm (**d**) and 100 μm (**g** and **i**).

We explored the molecular mechanisms underlying enhanced infiltration of neutrophils and/or monocytes by MBV and fluvastatin. We analyzed RNA-seq data and searched for differentially expressed cytokines, as these could act as attractants for neutrophils and/or monocytes in the ONC retina (Figs. 2 and 5). *Ccl2 (Mcp-1*, monocyte chemotactic protein-1) was amongst the top 30 DEGs by MBV and fluvastatin when compared to PBS (Fig. 5a). *Ccl2* encodes a key chemokine that mediates the recruitment of monocytes, where its receptor CCR2 is highly expressed (Fig. 3). *Ccl2* is up-regulated by the combined treatment when compared to PBS, and elevated expression of *Ccl2* was detected in all biological replicates (Fig. 5b, c). We next assessed the magnitude of CCL2 protein expression in the uninjured and injured retinae treated with the single or combined treatments or PBS as a control three days after ONC (Fig. 5d, e). We found that the combination of MBV and fluvastatin robustly increased expression of CCL2 only in the superficial retinal layer, and that CCL2 colocalized with GFAP^+^ cells where vimentin is co-expressed, suggesting CCL2 is upregulated in Müller glia^34^. We also tested whether CCL2 is highly upregulated by the combined treatment in other cells such as RGCs (Tuj1^+^ cells) and/or astrocytes (S100B^+^ cells)^34^ three days after ONC. We found that elevation of CCL2 expression was not detected in RGCs nor astrocytes (Supplementary Fig. 5). In contrast, MBV alone showed low levels of CCL2 expression, while fluvastatin alone had no effect (Fig. 5d). We then quantified CCL2 levels in each treatment condition (Fig. 5e). CCL2 expression was not significantly altered in the injured retina after ONC compared to the intact retina. In the injured retina, fluvastatin alone had no effect on CCL2 levels, but MBV alone resulted in a ~1.57-fold increase in CCL2 expression compared to PBS. The combined treatment more robustly upregulated CCL2 levels over single treatments, supporting the notion that monocyte infiltration by the combined treatment could be triggered by elevated expression of CCL2. In contrast to the retina, the combined treatment showed no increase in the number of neutrophils (Ly6G^+^ cells) and expression of CCL2 and CCR2 at the lesion site three days after ONC when compared to PBS (Supplementary Fig. 6a, b).

Next, we examined whether elevated CCL2 expression by the combined treatment contributed to monocyte infiltration after ONC (Fig. 5f). Using *Ccl2*^−/−^ mice^46^ and WT mice as a positive control, flow cytometric analysis revealed that fluvastatin and MBV promoted monocyte infiltration in WT mice, but not in *Ccl2*^−/−^ mice three days after ONC, while neutrophil infiltration was not affected in *Ccl2*^−/−^ mice. We then investigated whether loss of CCL2 impacts the combined MBV and fluvastatin treatment-mediated RGC protection (Fig. 5g, h) and axon regeneration after ONC (Fig. 5i, j). We found that enhancement of RGC protection and axon regeneration by the combination of MBV and fluvastatin was attenuated in *Ccl2*^−/−^ mice when compared to WT mice. Thus, MBV and fluvastatin synergistically evoke CCL2 expression in the retina, leading to enhancement of monocyte infiltration, which induces RGC protection and axon regeneration.

Activation of Müller glia leads to upregulation of CCL2 expression and infiltration of CCR2^+^ monocytes after light-induced injury^47^ or retinal detachment^48^, resulting in photoreceptor degeneration^49^. An additional concern is that prolonged intraocular inflammation might induce cataracts as unwanted side effects that limit the potential clinical use. Thus, we investigated whether MBV and fluvastatin induced cataract and affected thickness of other retinal cell layers, and/or the number of other retinal cells such as photoreceptors (Supplementary Fig. 7). We found that the combined treatment did not induce cataract two weeks after ONC as similarly seen in the injured retina treated with PBS (Supplementary Fig. 7a). Moreover, thickness of the whole and individual retinal layer such as INL and ONL, and other cell types like photoreceptors and bipolar cells were not affected by MBV and fluvastatin two weeks after ONC compared to the uninjured retina treated with PBS (Supplementary Fig. 7b–e). Thus, the combined treatment had little effects on induction of cataract and the number of other retinal cells.

### IL-33, an MBV-contained cytokine, acts synergistically with fluvastatin to enhance monocyte infiltration

**Fig. 6:**
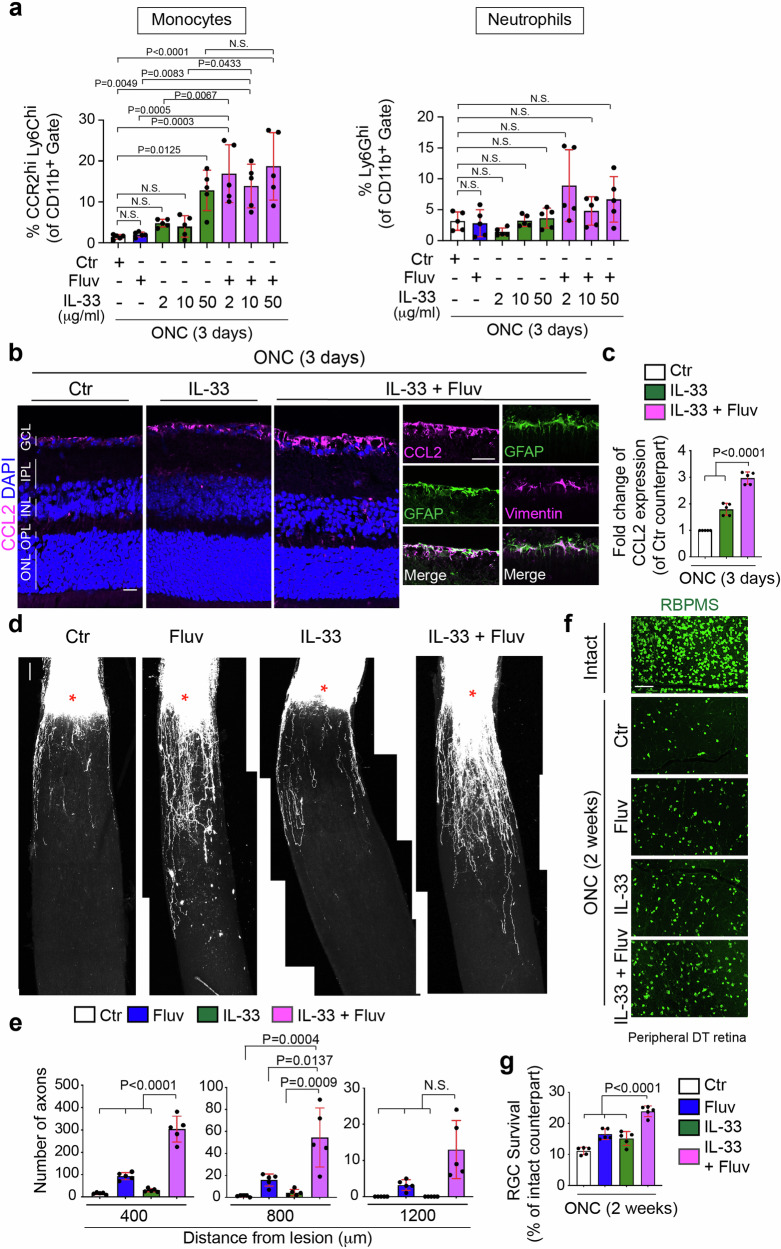
IL-33 acts synergistically with fluvastatin to enhance inflammatory monocyte infiltration, CCL2 expression and regenerative functions after ONC. **a** Quantification of percentages of neutrophils (Ly6G^hi^) and monocytes (CCR2^hi^Ly6C^hi^) among the CD11b^+^ myeloid cells in the retina treated with PBS (Ctr), fluvastatin alone, 2, 10 or 50 μg/ml IL-33 alone or the combination of 2, 10 or 50 μg/ml IL-33 and fluvastatin three days after ONC. (*n* = 5, one-way ANOVA with Tukey’s post-hoc). **b**, **c** Images of elevated expression of CCL2 protein in the retina with the combination of 10 μg/ml IL-33 and fluvastatin in Müller glia (GFAP^+^ cells expressing vimentin) three days after ONC compared to 10 μg/ml IL-33 alone or PBS (Ctr) (**b**), and quantitative analysis (**c**). (*n* = 5, one-way ANOVA with Tukey’s post-hoc). **d**, **e** Images of CTB^+^ regenerated axons in each condition two weeks after ONC (**d**) and quantitative analysis of the number of regenerated axons at 400, 800 and 1200 μm from the lesion site in each condition (**e**). (*n* = 5, two-way ANOVA with Tukey’s post-hoc). **f**, **g** Images of RBPMS^+^ RGCs in the intact retina or the injured retina in each condition two weeks after ONC (**f**), and quantitative analysis of RGC survival (%) based on the average of RGC survival in all four regions of the peripheral retina in each condition (**g**). (*n* = 5, one-way ANOVA with Tukey’s post-hoc). Data presented as mean ± SD. N.S. not significant; Scale bars represent 20 μm (**b**) and 100 μm (**d** and **f**).

The identities of MBV-contained factors that act with fluvastatin remain unknown. However, MBV derived from porcine urinary bladders and even other tissues consistently contain IL-33^16^. IL-33 mediates CCL2 expression in astrocytes and CCR2^+^ monocyte recruitment upon eukaryotic parasite infection in the brain^50^, making it an intriguing candidate. First, we confirmed whether ectopic expression of recombinant IL-33 alone increased monocyte infiltration after ONC, and found that 2 μl of 50 μg/ml, but not 2 and 10 μg/ml, of IL-33 protein significantly enhanced infiltration of monocytes three days after ONC (Fig. 6a). Next, we evaluated the overarching hypothesis that IL-33 in conjunction with fluvastatin could synergistically enhance monocyte infiltration as seen following treatment with the combination of MBV and fluvastatin. We found that 2 μl of 2 or 10 μg/ml of IL-33 alone or fluvastatin alone had little effect on monocyte infiltration, but the combination of 2 or 10 μg/ml of IL-33 and fluvastatin significantly increased monocyte infiltration after ONC to the same extent as 50 μg/ml of IL-33 alone. However, 50 μg/ml of IL-33 and fluvastatin did not show any further increased monocyte infiltration when compared to 50 μg/ml of IL-33 alone. Thus, fluvastatin potentiates the effects of IL-33 at low concentrations on monocyte infiltrations after ONC. In contrast, neutrophil infiltration was not affected by the combined treatments.

We next investigated whether the synergistic effects of IL-33 (10 μg/ml) and fluvastatin on monocyte infiltration is correlated with magnitudes of elevation of CCL2 expression (Fig. 6b, c), axon regeneration (Fig. 6d, e), and RGC protection (Fig. 6f, g) after ONC. First, we quantified CCL2 levels in the injured retina treated with IL-33 alone, the combination of IL-33 and fluvastatin, and PBS as a negative control (Fig. 6b, c). Although fluvastatin had no impact on CCL2 expression (Fig. 5d, e), the combination of IL-33 and fluvastatin more robustly increased CCL2 expression in the superficial layer of the retina, in particular by GFAP^+^/vimentin^+^ cells, Müller glia^34^ as similarly observed by MBV and fluvastatin treatment (Fig. 6b, c). Moreover, the combination of IL-33 and fluvastatin showed more robust axon regeneration and RGC protection when compared to either IL-33 or fluvastatin alone or PBS controls two weeks after ONC. Thus, the combination of IL-33 and fluvastatin showed enhancement of CCL2 expression and consequent monocyte infiltration, leading to RGC protection and axon regeneration after ONC. However, RGC protective and regenerative outcomes by IL-33 and fluvastatin were much lower than those by MBV and fluvastatin, which could be caused by little neutrophil infiltration in the retina.

### The combinatorial regenerative therapy with MBV, fluvastatin and AAV2 virus-mediated gene therapy

**Fig. 7:**
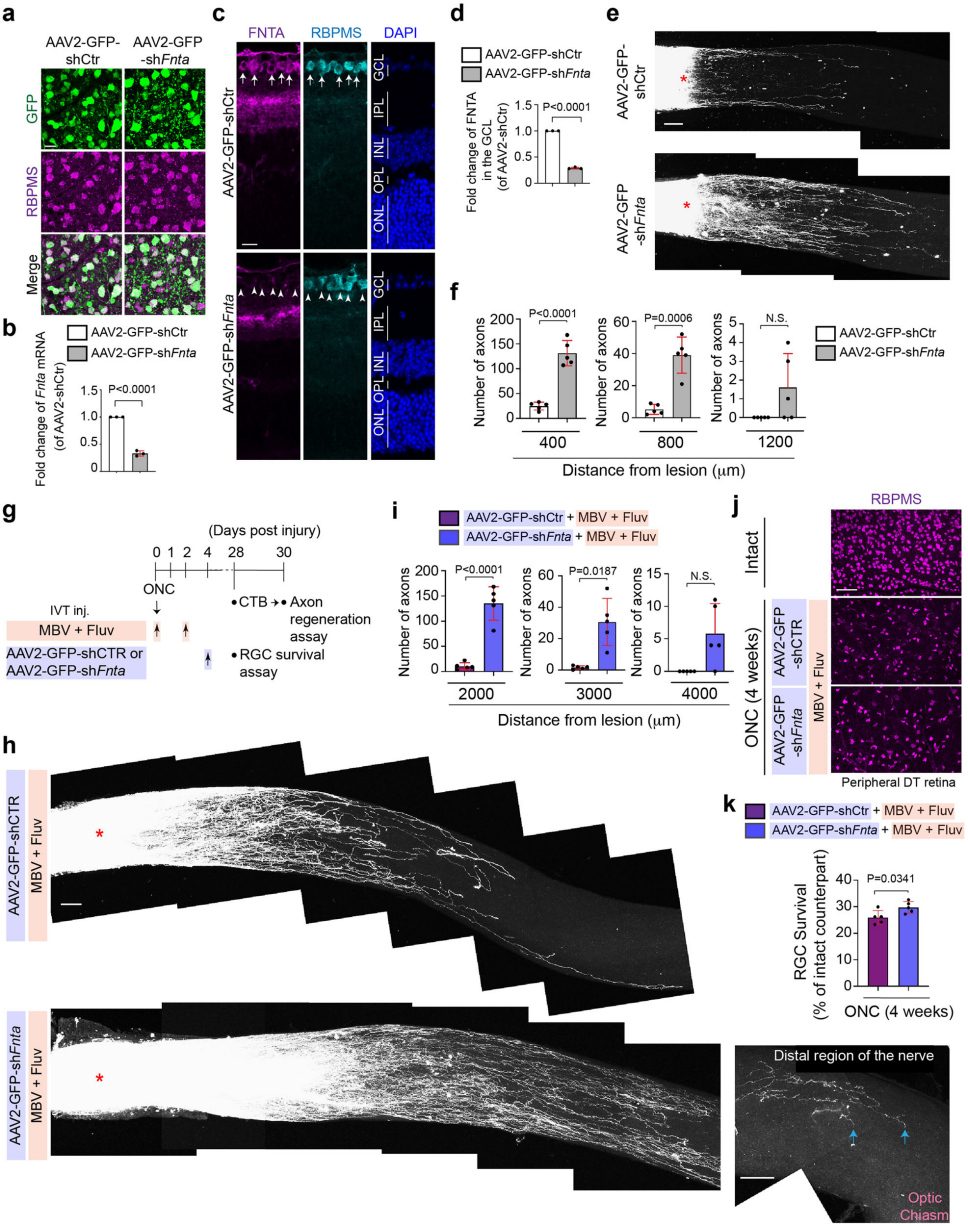
Extension of regenerated axons by the combination of fluvastatin, MBV and AAV2-shFnta virus after ONC. **a** Images of expression of GFP in RGCs (RBPMS^+^) of the retina infected with AAV2-GFP-shCtr or AAV2-GFP-sh*Fnta* viruses. **b** Validation of downregulated expression of *Fnta* mRNA by quantitative RT-PCR in the retina treated with AAV2-GFP-sh*Fnta* viruses compared to AAV2-GFP-shCtr viruses (*n* = 3, two-tailed unpaired t-test). **c**, **d** Downregulated protein expression of FNTA in the GCL (RBPMS^+^) by AAV2-GFP-sh*Fnta* viruses compared to AAV2-GFP-shCtr viruses (**c**) and quantitative analysis of fold-change of FNTA expression in the GCL (**d**). (*n* = 3, two-tailed unpaired t-test). **e**, **f** Images of CTB-labeled regenerated axons in the retina treated with AAV2-GFP-sh*Fnta* or AAV2-GFP-shCtr viruses two weeks after ONC (**e**), and quantitative analysis of the number of regenerated axons at 400, 800 and 1200 μm from the lesion site in each condition two weeks after ONC (**f**). (*n* = 5, two-way ANOVA with Bonferroni’s test). **g** Schema of axon regeneration analysis four weeks after ONC in the retina with intravitreal injections (IVT inj.) of the combination of MBV and fluvastatin with AAV2-GFP-sh*Fnta* viruses or AAV2-GFP-shCtr viruses post-injury. **h** Images of CTB^+^ regenerated axons in the retina treated with the combination of fluvastatin and MBV with AAV2-GFP-sh*Fnta* or AAV2-GFP-shCtr viruses four weeks after ONC. Tips of regenerated axons were observed near the optic chiasm, but they did not reach the brain (arrows). **i** Quantitative analysis of the number of regenerated axons at 2000–4000 μm from the lesion site in each condition four weeks after ONC. (*n* = 5, two-way ANOVA with Bonferroni’s test). **j**, **k** Images of RBPMS^+^ RGCs in the intact and injured retina in each condition four weeks after ONC (**j**), and quantitative analysis of RGC survival (%) based on the average of RGC survival in all four regions of the peripheral retina in each condition (**k**). (*n* = 5, two-tailed unpaired t-test). Lesion sites marked by asterisks (**e** and **h**). Data presented as mean ± SD. N.S. not significant; Scale bars represent 20 μm (**a** and **c**) and 100 μm (**e**, **j** and **h**).

To enable regenerated axons to extend longer in the injured retina treated with fluvastatin and MBV, we focused on adeno-associated virus (AAV)-based gene therapy, which stimulates intrinsic regenerative signals in RGCs (Fig. 7). To identify an AAV-mediated therapeutic partner, which is compatible with fluvastatin and MBV, we targeted a known statin-mediated downstream pathway, protein prenylation^3,5^. Statins inhibit protein prenylation pathways, and our previous study demonstrated that prenylation inhibitors, FTI and GGTI promote axon outgrowth even on inhibitory substrates in vitro and enhance retinal axon regeneration after ONC similar to the effect of statins^3^. Although FTI and GGTI each block distinct enzymes, FTase- and GGTase-I-mediated prenylation pathways respectively, farnesyltransferase (FNTA, encoded by *Fnta*) is a common component of both FTase and GGTase-I, suggesting that knocking down of *Fnta* can mimic functions of prenylation inhibitors FTI and GGTI in neurons. Thus, we tested the effects of AAV2-sh*Fnta* viruses alone and the combination of MBV, fluvastatin and AAV2-sh*Fnta* viruses on axon regeneration after ONC.

First, we investigated virus infection and knockdown efficiency of AAV2-GFP-sh*Fnta* viruses compared to AAV2-GFP-shCtr viruses two weeks after intravitreal injections. We confirmed that two weeks after injections of AAV2-GFP-sh*Fnta* or AAV2-GFP-shCtr viruses, 92.9% or 95.5% of RGCs express GFP, respectively (*n* = 5, 1039 RGCs in the retinae with AAV2-GFP-sh*Fnta* virus and 831 RGCs in the retinae with AAV2-GFP-shCtr virus were analyzed in total, N.S., two-tailed unpaired t-test) (Fig. 7a). We also confirmed by qPCR that *Fnta* mRNA levels in the whole retina were reduced by 67.0% in the AAV2-GFP-sh*Fnta*-injected retina, when compared to AAV2-shCtr virus-injected retina (Fig. 7b). FNTA protein expression was detected in the ganglion cell layer (GCL) and inner plexiform layer (IPL), and the expression in the GCL was reduced by 71.0% by AAV2-GFP-sh*Fnta* viruses compared to AAV2-GFP-shCtr viruses (Fig. 7c, d). Following AAV2-GFP-sh*Fnta* or control virus infection two weeks prior to ONC, we performed ONC and then examined the number of regenerated axons in the injured nerve two weeks after injury (Fig. 7e, f). We confirmed that AAV2-GFP-sh*FNTA* virus increased the number of regenerated axons, which extended up to 800 μm from the lesion site when compared to AAV2-GFP-shCtr virus.

Next, we examined whether injections of AAV2-GFP-sh*Fnta* viruses post-injury can further robustly elongate regenerated axons in the retina treated with the combination of fluvastatin and MBV (Fig. 7g, i). We performed intravitreal injections of fluvastatin and MBV a few minutes and two days after ONC, and AAV2-GFP-sh*Fnta* viruses four days after ONC, and analyzed axon regeneration four weeks instead of two weeks after ONC because AAV-mediated gene transduction is slow: at least one to two weeks are needed to reach robust expression of transgenes and then enhance regenerative outcomes (Fig. 7g). We found that all combinations - fluvastatin, MBV and AAV2-GFP-sh*Fnta* viruses robustly enhanced axon regeneration and increased the number of extending regenerated axons from the lesion site. For example, at 2000 μm from the lesion site, 135 regenerated axons were detected in the injured nerve treated with the combination of fluvastatin and MBV with AAV2-GFP-sh*Fnta* viruses, while only 11 axons were extended by fluvastatin, MBV and control viruses. The regenerated axons extended up to 3000 μm from the lesion site, but the tips of regenerated axons did not reach the brain (Fig. 7h, i). In contrast, RGC survival was slightly enhanced by all three combinations of fluvastatin and MBV with AAV2-GFP-sh*Fnta* viruses compared to fluvastatin and MBV with control viruses (Fig. 7j, k). Thus, the combinatorial therapy with MBV, fluvastatin and AAV2 virus-mediated inhibition of prenylation pathways in RGCs is effective for promoting further regenerative outcomes post-injury.

## Discussion

To explore novel clinically relevant therapies that protect neurons from death and promote nerve regeneration after optic nerve damage, our independent previous studies have shown that statins were identified as reagents enhancing axon regeneration after ONC^3^, while MBV were successfully isolated from ECM biomaterials and functionally characterized as immunomodulatory reagents for RGC protection^13,21^. In this study, we have extended previous works on the potential synergistic effects of the combination of statin/fluvastatin and MBV on axon regeneration and RGC protection after ONC, by which infiltration of monocytes and neutrophils is enhanced. We also demonstrated that combining non-cell autonomous regenerative mechanisms by fluvastatin and MBV with blocking anti-regenerative pathways in RGCs by AAV2 viruses knocking down *Fnta* led to improvement of regenerative outcomes. Thus, the molecular assembly from distinct therapeutic reagents synergistically promotes neuroprotection and axon regeneration after nerve injury.

This study demonstrated that infiltration of neutrophils and monocytes is enhanced by the combination of fluvastatin and MBV after ONC. Despite the fact that statins or MBV alone inhibit pro-inflammatory responses, leading to neuroprotection in several neurodegenerative diseases^7–9,21^, we found that when MBV and fluvastatin are co-injected, neutrophils and monocytes are robustly infiltrated, resulting in enhancement of RGC protection and axon regeneration after ONC. These opposite immunomodulatory outcomes may be achieved by which a specific pair of statin and cytokine(s) appears and in turn synergistically acts to produce other cytokine(s) to modulate inflammation responses. To strongly support the notion, our study revealed that the combination of fluvastatin and IL-33, a cytokine enriched within MBV, mimics the effects of fluvastatin and MBV on enhancement of monocyte infiltration by increasing CCL2 expression by Müller glia in the superficial layer of the retina, and the other study has shown that statin and IL-2 synergistically induce IFN-γ production in peripheral blood mononuclear cells while either statin or IL-2 alone fails to upregulate IFN-γ expression^10^. Thus, the present study contributes to identification of novel molecular mechanisms underlying neuroprotection and axon regeneration by statin and MBV via specific cytokine(s). The molecular mechanisms of how these two different reagents regulate expression of CCL2 and other genes involved in neutrophil infiltration remain unclear. However, previous studies have shown that IL-33 upregulates CCL2 expression through controlling MAPK and NF-κB pathways^51^, and statins activate or suppress NF-κB pathways depending on cell types and cellular events^52,53^, suggesting that fluvastatin and MBV may cooperate to modulate NF-κB pathways and/or other signaling pathways, leading to modulation of expression of the relevant genes.

Several concerns and biological questions on the combination therapy remain unclear. It might be concerned that the extent and effects of MBV and fluvastatin on cellular outcomes vary because MBV show heterogeneity depending on different tissue sources^18^. However, MBV, which are derived from porcine urinary bladders used in this study and other tissues, consistently contain IL-33^16^. We confirmed that at least two different batches of MBV from the same porcine urinary bladders, when combined with fluvatstain, showed the same magnitude of RGC protection and axon regeneration after ONC.

The other concern is that either fluvastatin or MBV alone showed narrow therapeutic windows compared to PBS. However, the combined treatment induced much more robust regenerative and neuroprotective outcomes compared to PBS, and either fluvastatin or MBV alone. Thus, when they are applied together, fluvastatin and MBV retain much higher efficiency in promoting neuroprotection and axon regeneration than PBS alone, suggesting that instead of single use, the combined treatment could be clinically more beneficial.

Regarding the magnitude of axon outgrowth on the inhibitory substrate, MAG, our previous study ranked the most potent statins based on EC_50_, and we determined that among clinically available statins, fluvastatin is the most effective statin while simvastatin is the second most effective statin. Thus, we have chosen fluvastatin in this study. However, simvastatin more effectively lowers cholesterol levels than fluvastatin^54,55^, and simvastatin protects RGCs after ischemia/reperfusion injury^56^ and chronic ocular hypertension^57^. Thus, future comparative studies could demonstrate differential magnitude of neuroprotective and regenerative outcomes depending on types of statins with MBV.

Based on molecular signatures in specific RGC subtypes^28,58,59^, our bulk-RNA seq revealed that for instance, expression levels of *Penk* (N-RGCs and susceptible type to ONC), *Fes* (alpha RGCs and resistant type to ONC), *Chl1* (ipRGCs and resistant type to ONC) are higher in the retina treated with the combined treatment compared to PBS, suggesting that the combined treatment may maintain resistant several RGC types and also promote survival of some of the specific susceptible RGCs. Further studies using single RNA-seq analysis in RGCs will clarify the effects of the combined treatment on protection of specific or global types of RGCs after ONC.

There is a concern that our current study showed lower RGC survival in the control injured retina (e.g. 12.2% ±0.8 (Fig. 1e), 12.7% ±0.9 (Fig. 4c)) compared to other studies^60,61^ demonstrating ~20% of RGC survival in the control. However, several studies have similarly shown 10–15% of RGC survival in control injured retinae after rodent optic nerve, in which they intravitreally injected viruses or reagents after nerve crush^62–65^, suggesting that mechanical damages to the retina, sizes of needles for injections, and/or injection frequencies may reduce basal RGC survival level. Thus, such non-invasive gel-formed delivery system^66^ could increase basal RGC survival level. If so, the safe delivery system carrying fluvastatin and MBV could also increase RGC survival from ~30%.

It remains two open questions as to molecular mechanisms underlying the effects of the combined treatment. The first question is how neutrophil infiltration is enhanced by the combination of MBV and fluvastatin. CXCR2 is a major chemokine receptor expressed by neutrophils, and *Cxcr2* is the most differentially expressed gene (21.8-fold increase) following the combination of MBV and fluvastatin compared to PBS, suggesting neutrophil infiltration could be triggered by elevated expression of CXCR2 in the retina treated with the combined treatment. However, well-characterized ligands for CXCR2, CXCL1-3, 5–8^67^ are barely expressed in the injured retina and are not upregulated by the combined treatment, suggesting that other molecular mechanisms could mediate neutrophil recruitment. For instance, CCL3 has been implicated to mediate neutrophil infiltration^68^. RNA-seq results revealed that *Ccl3* is moderately upregulated (2.55-fold) by the combined treatment compared to PBS. The receptors for CCL3, *Ccr1* and *Ccr5* are also upregulated (3.13-, 2.12-fold change) by the combined treatment. Thus, CCL3 and/or other cytokines could act as attractants for neutrophil recruitment in the injured retina following the combined treatments after ONC.

A subset of neutrophils infiltrated by zymosan/anti-CXCR2 treatment shows high expression of *Il4ra* and *Mrc1* transcripts^69^. These cells also secrete growth factors such as IGF-1 and NGF and stimulate regrowth of axons after optic nerve injury. Our bulk RNA-seq data revealed that MBV and fluvastatin upregulated expression of two genes, *II4ra* (1.22 fold-change) and *Mrc1* (1.54 fold-change). However, expression of growth factors *Igf1* and *Ngf* is not altered by the combined treatment. Thus, this subset of neutrophils may be infiltrated by the combined treatment, but molecular signatures in these neutrophils may be altered post-infiltration.

The second question is what regenerative and neuroprotective factors are expressed in those infiltrating immune cells. There is increasing evidence that infiltrating immune cells express regeneration-enhancing cytokines, trophic factors, and other potential regenerative factors such as CNTF^42^, oncomodulin^70,71^, IL-6^72^ and SDF-1 (Cxcl12)^73^, which stimulate axonal regenerative signals in RGCs. However, our RNA-seq data showed that expression of these factors is not affected by fluvastatin and MBV. In contrast, the combined treatment upregulates neurotrophic factor *Bdnf* (1.43 fold-change)^74^ and axon growth-stimulating factor *Fgf2* (1.88 fold-change)^75^, which are known to be secreted from immune cells including monocytes^76,77^. These molecules could mediate axonal regeneration and/or RGC protection. However, more studies are needed to identify uncharacterized regenerative and neuroprotective factors expressed in monocytes and neutrophils infiltrated by fluvastatin and MBV.

While MBV and fluvastatin can be utilized as therapy that can be administered shortly after nerve injury, AAV virus-mediated gene therapy is promising for long-term regenerative treatments of neurodegenerative diseases and after nerve injury. Multiple intravitreal injections of fluvastatin and MBV may show more robust regenerative and neuroprotective outcomes, but we were concerned about mechanical retinal damage and unwanted inflammation caused by multiple injections. Instead of the repeated procedures, we chose to utilize AAV-based gene manipulations after acute nerve injury because a single injection of AAV viruses is sufficient to transduce or downregulate the target gene such as *Fnta* and this limits additional retinal damage. Our results revealed that post-injury intravitreal injections of the combination of fluvastatin and MBV with AAV2 virus-mediated silencing of *Fnta* successfully enabled regenerated axons to extend longer compared to fluvastatin and MBV combined treatment. Indeed, inhibition of prenylation pathways inactivates target proteins, leading to a wide range of therapeutic benefits for treatments of cancer, neurodegenerative diseases and viral infections^78^. Our previous study also demonstrated that protein prenylation pathways in neurons exert anti-regenerative effects^3^. Rho, Ras, Ral, and other GTPases are prenylated by FTase or GGTase-I, whose common enzyme component is FNTA. Intriguingly, loss of one of the prenylated targets such as RhoA in RGCs successfully promotes optic nerve regeneration^79,80^, which is consistent with our observations that FNTA is highly expressed in RGCs, and knockdown of *Fnta* helps regenerated axons extend longer when combined with fluvastatin and MBV after ONC. However, Ras and Rac positively regulate axon regeneration and growth^81,82^. Thus, it will be important for future studies to complete protein prenylation profiling in RGCs where *Fnta* is downregulated, in order to develop a better mechanistic understanding of which targets are unprenylated to contribute to axon regeneration and extension of regenerative axons.

There are two reasons why we chose AAV2-sh*Fnta* virus instead of prenylation inhibitors and we injected the viruses post-infiltration of monocytes and neutrophils: (i) because prenylation targets such as RhoA, Cdc42, Rac and Ras are involved in regulation of not only axon outgrowth/regrowth but also motility and migration efficiency of neutrophils, monocytes and other immune cells^83–85^, and (ii) a single injection of AAV viruses carrying sh*Fnta* is sufficient to suppress prenylation pathways while FTI/GGTI prenylation inhibitors must be intravitreally injected multiple times post-injury, which could cause severe damage to the retina. However, the molecular mechanisms of how AAV2-sh*Fnta* virus potentiated the regeneration outcomes following MBV and fluvastatin treatments are still unclear. Thus, further in-depth single RNA-seq analysis in RGCs could determine whether different or same regenerative mechanisms are driven by these distinct therapeutic interventions.

Toward establishing clinical treatments of ocular trauma and optic neuropathies, a number of studies have described intrinsic and extrinsic molecules and signaling pathways that mediate RGC death/survival and axon degeneration, and axon regeneration using the rodent optic nerve injury models^28,32,42,60,61,65,86–102^. Identification of regenerative and neuroprotective genes and molecular mechanisms is critical to development of new therapeutic drugs. However, the major barrier in the field is that it usually takes 10 to 20 years from bench to clinical applications of new drugs, and success rates are extremely low. Furthermore, even first-aid strategies, which can be effective shortly after injury, do not exist to treat injured RGCs. To circumvent this issue, results in this study revealed the novel regenerative and neuroprotective strategies based on an FDA-approved drug or components of the FDA-approved ECM biomaterials, statin and MBV, suggesting this combination therapy could be easily approved for clinical applications. However, this combined treatment showed still limited capacity of optic nerve regeneration. Thus, the further combinatorial therapy containing MBV, fluvastatin and other FDA-approved validated drugs or molecular interventions and/or AAV viruses carrying regeneration-associated genes in addition to sh*Fnta* should be considered. For instance, an FDA-approved drug, glycopyrrolate could be a potential partner because it stimulates axon regeneration to re-innervate the central visual target areas after optic nerve injury through controlling intrinsic regenerative signals^103^. Thus, further comparative studies will identify new combination partners based on (i) application forms, frequencies and timing (e.g. compounds/small molecules vs AAV viruses), (ii) different cellular mechanisms (e.g. cell- vs non-cell-autonomous actions), and (iii) different molecular mechanisms (e.g. prenylation pathways vs other pathways), leading to identification of the most easily applicable and effective combinatorial strategies.

Our results suggest that the combination therapy, fluvastatin and MBV could be applied as therapeutic molecular interventions acting in non-cell-autonomous manner. However, establishing clinically viable treatments based on retinal/vitreous inflammation is controversial. Our data showed that fluvastatin and MBV therapy had no induction of cataract and no effects on thickness of other retinal layers and the number of photoreceptors and bipolar cells after ONC. These outcomes could be explained by two reasons: (1) infiltration of monocytes and neutrophils by the combined treatment is transient, and (2) ~ 80% of infiltrating neutrophils and monocytes are located only within the GCL and IPL or in the intravitreal area. Yet, it still remains unclear whether the combined treatment is safe to human retinal cells. Thus, the combination therapy using statin and specific cytokines enriched within MBV (e.g. IL-33) or driving intrinsic regenerative mechanisms in RGCs by the combination therapy could be an alternative approach to augment axon regeneration and RGC protection.

## Methods

### Ethics approval

All mice experiments were performed according to the guidelines of the Animal Care and Use Committee at the University of Pittsburgh School of Medicine (#21039031, #24024545).

### Animals

C57BL/6J mice (referred to as WT, Jackson Laboratory, JAX: 000664), *Ccr2*^*−/−*^ mice (Jackson Laboratory, JAX: 004999), and *Ccl2*^*−/−*^ mice (Jackson Laboratory, JAX: 004434) were used in the study. Male and female mice were used in this study with equal distributions for all experiments. All mice were housed in a pathogen-free barrier facility and were maintained in a 12 h light/dark cycle with access to standard laboratory chow and water *ad libitum*. As previously described^1^, for surgeries and intravitreal injections, 8-week-old animals were anesthetized with an intraperitoneal injection of ketamine/xylazine (100/10 mg/kg body weight). Before and after surgeries and injections, bupivicaine (4 mg/kg, <0.2 ml) and buprenorphine (0.1 mg/kg, <0.3 ml) were subcutaneously injected. After surgeries and injections, proparacaine and vetropolycin were dropped on the surface of the eyes. For euthanasia, CO_2_ was used with the flow rate, 30–70% per minute.

### Optic nerve crush

The optic nerve in the left eye was crushed using #5 Dumont forceps, applying pressure for 5 s at ~5 mm behind the optic disc, while the right eye remained uninjured with an intact retina. Following various time points after optic nerve crush, animals were humanely euthanized with CO_2_ and perfused with 4% paraformaldehyde (PFA) following PBS. The heads were cut and post-fixed in 4% PFA overnight at 4 °C and washed with PBS for further analysis. For double-immunostaining with antibodies for GFAP and vimentin, the whole retina was fixed with 4% PFA for 30 min without perfusion.

### Intravitreal injection

For intravitreal injections, glass micropipettes made by a micropipette puller was used. Fluvastatin (Sigma, SML0038) was dissolved in the sterile ddH_2_O or PBS at 37 °C, and stored at −20 °C. To make the combination of 1.3 μg/μl fluvastatin with 2.3 × 10^9^ particles/ml MBV or 2, 10 or 50 μg/ml IL-33 (Pepro Tech, 210-33), 2.6 μg/μl fluvastatin solution was mixed with 4.6 × 10^9^ particles/ml MBV or 4, 20 or 100 μg/ml IL-33 in a 1:1 ratio prior to injections. A hole in the middle region of the retina was made with the glass micropipette to inject 2 μl of the first treatments or PBS within a few minutes after ONC, and then the second treatments or PBS were injected through the same retinal hole two days after ONC. For axon regeneration assay, 2 μl of 2 μg/μl of Alexa Fluor 555 conjugated cholera toxin β subunit (CTB) (ThermoFisher, C22843) were injected into the retina 2 days before euthanasia. 2 μl of AAV2-CMV-GFP-shCTR virus (control sequences:AGTATATCTATGCTTCTATCCGCTCAGGTGTATATCCTTGGATAGTGGCGTAACAACG, 4 × 10^13 GC/ml, VIROVEK)^1^ or AAV2-CMV-GFP-sh*Fnta* virus (target sequences: CCGGGAACTACATCACTGCGATAATCTCGAGATTATCGCAGTGATGTAGTTCTTTTTTG, 4 × 10^13 GC/ml, VIROVEK) were intravitreally injected two weeks before ONC or four days after ONC.

### Intraperitoneal injection

Anti-mouse Ly6G antibody (clone 1A8) (200 μg, Bio Cell, BE0075-1) or control IgG2a antibody (200 μg, Bio Cell, BE0089) was diluted in the sterile PBS, and intraperitoneally injected into C57BL/6J and *Ccr2*^*−/−*^ mice two days and one day before ONC and a few minutes and one, two and three days after ONC.

### Preparation and quality control of MBV

MBV were be prepared from porcine urinary bladder ECM (UB-ECM) as previously described^13,14,19,104^. Porcine urinary bladders were obtained from Animal Biotech Industries. The luminal urothelial cells of the tunica mucosa within the bladders were dissociated from the basement membrane by washing with deionized water, and then decellularized and digested by agitation in 0.1% peracetic acid with 4% ethanol for 2 hours at 300 rpm. The tissue was washed with 1× PBS (pH 7.4) and sterile deionized water, and then lyophilized and milled into particulate. To isolate MBV, the UB-ECM was then digested with Liberase TL (highly purified collagenase I and collagenase II) in buffer [50 mM tris (pH 7.5), 5 mM CaCl_2_, and 150 mM NaCl] for 24 hours at room temperature. Digested tissues was then subjected to centrifugation at 10,000 *g* for 30 min at 4 °C and filtered through a 0.22-μm filter. The supernatant was then centrifuged at 100,000 *g* (Beckman Coulter Optima L-90K Ultracentrifuge) at 4 °C for 70 min to pellet the MBV. MBV were then resuspended in sterile 1× PBS (pH 7.4), and particle concentration was determined using particle nanotracking analysis. The size and concentration of freshly isolated MBV were measured using Nanosight nanoparticle tracking analysis (Malvern Panalytical). The Brownian motion of the vesicles was used to determine the size distribution as measured across three replicates of 45 s videos for each sample. Vesicle dosages were described in the main figure and text. Transmission electron microscopy (TEM) imaging was performed as previously described in our study^16^.

### Immunohistochemistry and imaging

Procedures and methods for whole mount immunofluorescent staining of the injured and intact retina were previously described^1^. Each retina with a small cut only in the peripheral ventral region was incubated with primary antibodies in PBS containing 0.4% Triton X-100 (PBST) overnight at 4 °C and then washed with PBST three times. The retina was incubated with following secondary antibodies in PBST overnight at 4 °C and washed with PBST three times. The dorsal, nasal and temporal regions in the intact and injured retina were also cut with micro scalpels on the microscope slide and mounted with Fluoro-Gel mounting medium (Electron Microscopy Sciences, 17985-11). For cryosectioning, the retina was incubated in 30% sucrose in PBS overnight at 4 °C, and then embedded with Tissue-Tek O.C.T.. 14 μm retinal cryosections were prepared and immunostained described above. For immunostaining with antibodies for Ly6G, Ly6C, CCR2, CCL2, GFAP, vimentin, Rhodopsin, S100B, F4/80, PNA and PKCα, cryosections were incubated with MeOH for 2 mins and washed with PBS, and sections were incubated with the primary antibodies in PBS overnight at room temperature and with the secondary antibodies in PBS for a few hours at room temperature. Immunolabeling was performed with the following primary antibodies: rabbit βIII-tubulin (1:500, Abcam, ab52623), guinea pig RBPMS (1:500, Millipore, ABN1376), chick GFAP (1:500, ThermoFisher, PA1-10004), mouse CCR2 (1:500, R&D systems, MAB150), mouse CCL2/MCP-1 (1:300, ThermoFisher, MA5-17040), Alexa Fluor 594 Ly6G (1:300, Biolegend, 127636), and Alexa Fluor 488 Ly6C (1:300, Biolegend, 128021), chick GFP (1:600, Abcam, ab13970), rabbit vimentin (1: 500, ThermoFisher, PA5-27231), rabbit FNTA (1:400, SantaCruz, sc-374262), rabbit S100B (1ː 300, ThermoFisher, PA5-78161), rat F4/80 (1ː 300, BioRad, MCA497RT), mouse Rhodopsin (1D4, 50 μg/μl, Donated by Krzysztof Palczewski Lab), Alexa Fluor 488 PNA (1:500, ThermoFisher, L21409) and mouse PKCα (1ː400, ThermoFisher, MA1-157). Secondary antibodies were used at 1:500 and purchased from Lifetechnology: goat anti-Guinea Pig IgG 647 (A21450), goat anti-Rabbit IgG 594 (A-11037), donkey anti-Mouse IgG 647 (A-31571), donkey anti-Rabbit IgG 647 (A-31573), and goat anti-Chick IgG 488 (A11039), donkey anti-Goat IgG 488 (A-11055). DAPI was used at 1:500 and purchased from ThermoFisher (D1306).

Olympus Fluoview 1000 laser scanning microscope with Fluoview software, a 20× objective lens (PlanSApo, NA = 0.85) and a 40× objective lens (UPlanFL N NA = 1.3) was used for imaging.

### Flow cytometry

Each injured left retina with defined treatments was dissected out in cold PBS three or seven days after ONC and collected for flow cytometry. The whole retina was treated with collagenase type I (84 U/cornea; Sigma-Aldrich), dissociated and incubated for an hour at 37 °C. The single-cell suspension of each retina was then filtered through a 40 μm cell strainer cap (BD Labware) and washed with FACS buffer. Cell suspensions were then exposed to Fc block and Zombie Viability Dye in Zombie Aqua™ fixable viability kit (BioLegend, 423101) for 15 min at 4 °C. After washing with PBS + 2% FBS, cells were stained with the fluorescent antibodies, PE CD11b (BioLegend, 101207), BV605 CD192 (CCR2) (BioLegend, 150615), BV650 Ly6G (BioLegend, 127641) and FITC Ly6C (BD Pharmingen, 553104). After a 20-min incubation, cells were washed two times with PBS + 2% FBS. Cells were then fixed with 4% PFA and analyzed by flow cytometer (Beckman Coulter, Cytoflex LX) with FlowJo software (TreeStarInc., Ashland, OR).

### Quantitative RT-PCR

Procedures and methods for quantitative RT-PCR of the whole retina were previously described^1^. Quantitative PCR was performed in duplicate using iTaq Universal SYBR Green (BioRad, 1725121) and the CFX384 Touch Real-Time PCR Detection System (BioRad). Results were presented as linearized Ct values normalized to *Actb* gene. The results of qRT-PCR in the injured retina treated with the combined treatment were normalized to the mean value of the injured retina with PBS two days after ONC. Experiments were repeated each condition with specific primers described in PrimerBank: *Cxcr2* (Forward: 5′-ATGCCCTCTATTCTGCCAGAT-3′, Reverse: 5′-GTGCTCCGGTTGTATAAGATGAC-3′), *Atp8b4* (Forward: 5′-GAGAAGTTCCAGTATGCGGAC-3′, Reverse: 5′-TGACAGCCGTCATCGAGATCA-3′), *Ccr2* (Forward: 5′-ATCCACGGCATACTATCAACATC-3′, Reverse: 5′-CAAGGCTCACCATCATCGTAG-3′), *H2-Aa* (Forward: 5′-TCAGTCGCAGACGGTGTTTAT-3′, Reverse: 5′-GGGGGCTGGAATCTCAGGT-3′), *Cd209a* (Forward: 5′-CTGGCGTAGATCGACTGTGC-3′, Reverse: 5′-AGACTCCTTGCTCATGTCAATG-3′), and *Actb* (Forward: 5′-GGCTGTATTCCCCTCCATCG-3′, Reverse: 5′-CCAGTTGGTAACAATGCCATGT-3′). To validate *Fnta* knockdown efficiency, the intact retinae infected with AAV2-CMV-GFP-shCTR virus and AAV2-CMV-GFP-sh*Fnta* virus two weeks after infection were collected, and quantitative PCR was performed in triplicate with specific primers for *Fnta* described in PrimerBank: *Fnta* (Forward: 5′-CCCTATGGACGACGGGTTTC-3′, Reverse: 5′-TGATCTGGACCACTGGGTTAG-3′).

### RNA sequencing

Preparations of the retina for bulk RNA-seq and data analysis were previously described^1^. Injured retinae treated with the single or combined treatments two days after ONC were collected, and RNA was extracted using TRIzol RNA isolation reagent (ThermoFisher). This sample preparation was conducted in triplicates for each condition. RNA quality was verified by the University of Pittsburgh Health Genomics Core using Agilent Tapestation RNA and Qubit Fluorometric Quantification. RNA samples with the RNA integrity number of (RIN) > 7 were utilized to synthesize cDNA. Library preparation was conducted with TruSeq Total RNA library kit (Illumina) using at least 1 μg of total RNA following the manufacture’s instruction. Libraries were sequenced using NextSeq500 (Illumina) with 75 base paired end reads resulting in 40 M reads per sample. FASTQ files were analyzed using CLC Genomics Grid Workbench v22.0.2. For RNA sequencing analysis, sequenced reads were aligned to the mouse genome (GRCm39). Read counts were generated and differentially expressed genes were obtained using CLC Genomic Workbench v22.0.2. Transcripts with more than 10 read events in two samples of either treatments or control PBS with a p-value lower than 0.05 among three were collected for further analysis. For further expression shown in heatmaps and volcano plots and GO-pathway analysis, transcripts which show the false discovery rate (FDR) *p*-value (<0.05) and a fold change higher than 2 or the highest changes were selected. Heatmaps and volcano plots were generated using CLC Genomic Workbench v23.0.1 and GraphPad – Prism 9.0. Ingenuity Pathway Analysis was performed on DEGs with an FDR *p*-value < 0.05 and a Fold Change ≥2. Pathway z-scores were generated using the Expression Log Ratio. To draw Venn diagrams, Venn diagram online analysis provided by VIB/UGent Bioinformatics & Evolutionary Genomics was used (https://bioinformatics.psb.ugent.be/webtools/Venn/).

### RGC survival analysis

RGC survival analysis in the whole mount retina was performed by counting the RBPMS^+^ RGCs in a 400 μm × 600 μm area in four quadrants, ventrotemporal (VT), ventronasal (VN), dorsotemporal (DT) and dorsonasal (DN) of the peripheral or the middle retina of the injured left eye and intact contralateral right eye in the same animal. The region-specific RGC survival (%) was provided by dividing the RGC number in each corresponding region of the injured retina by the number in the intact retina. The average RGC survival in all retinal regions (%) was provided by dividing the total RGC number from all four retinal regions of both peripheral and middle or peripheral injured retina by the total number from those regions in the intact retina.

### Immune cell infiltration analysis of cryosectioned retinal samples

For quantification of Ly6G^+^ cells and CCR2^+^Ly6C^hi^, we examined five 14 μm non-consecutive cryosections of the central retina. Total number of Ly6G^+^ cells or CCR2^+^Ly6C^hi^ from two 300 μm (width) retinal areas adjacent to the nerve head per section was provided as shown in Fig. 3f, and the average from five sections per animal was calculated. To provide the localization preference of those immune cells within the retina (%), those counted immune cells were sorted based on three retinal layers, 1: Intravitreal (IV), Ganglion cell layer (GCL) and Inner plexiform layer (IPL), 2: Inner nuclear layer (INL) and Outer plexiform layer (OPL), 3: Outer nuclear layer (ONL) and Outer Limiting Membrane (OLM), and the number of those infiltrating cells in each layer was divided by the total number of infiltrating immune cells per section, and the analysis was repeated in five sections per animal. To examine the magnitude of depletion of monocytes and neutrophils, we examined four 14 μm non-consecutive cryosections of the central retina. The total number of Ly6G^+^ cells or Ly6C^hi^ cells in the intravitreal (IV), ganglion cell layer (GCL) and inner plexiform layer (IPL) from two 300 μm (width) retinal areas adjacent to the nerve head per section was provided, and the average from four sections per animal was calculated.

### Expression analysis of cryosectioned retinal and optic nerve samples

For quantification of CCL2 fluorescent signals, four 14 μm non-consecutive retinal cryosections in the temporal retina where the single or combined treatments or PBS were intravitreally injected, were used per retina. Images were taken with the same setting of the inverted confocal microscope. The mean pixel intensity of 200 μm × 20 μm area centered on CCL2^+^ retinal surface was measured with Fiji software in each cryosection. After the average of the mean pixel intensity in the control conditions from four sections per animal such as intact retina or injured retina with PBS was obtained, we calculated the fold change of CCL2 expression in each condition based on the average value in the control samples.

For quantification of FNTA fluorescent signals, three 14 μm non-consecutive retinal cryosections in the retina were used per retina, and imaged with the same setting of the inverted confocal microscope. The mean pixel intensity of 200 μm × 20 μm area centered on the FNTA^+^ RGC layer was measured and the average of pixel intensity was provided from three sections per retina. The fold-change of FNTA expression was calculated in the retina treated with AAV2-GFP-sh*Fnta* virus compared to AAV2-GFP-shCtr virus.

For quantification of F4/80^+^ cells, we prepared four 14 μm non-consecutive retinal cryosections per animal, and we counted F4/80^+^ cells from two 300 μm (width) retinal areas close to the optic nerve head, provided the average number per section, and repeated the analysis in four sections to obtain the final average number per animal.

For quantification of PNA^+^ cells and Rhodopsin or PKCα fluorescent signals, four 14 μm non-consecutive retinal cryosections per animal were prepared. We counted PNA^+^ cells or measured signal intensities of Rhodopsin or PKCα with Fiji software within 200 μm (width) retinal areas adjacent to the nerve head per section, and then the average number or fold change of expression per animal were provided.

For quantification of CCR2 or CCL2 fluorescent signals at the lesion site of the nerve, two 14 μm non-consecutive optic nerve cryosections per animal were prepared. We measured signal intensities of CCR2 or CCL2 with Fiji software in 300 μm (width) lesion areas, and then provided the average fold change of expression per animal. For quantification of Ly6G^+^ cells at the lesion site of the nerve, three 14 μm non-consecutive optic nerve cryosections per animal were prepared. We counted Ly6G^+^ cells in 300 μm (width) lesion areas, and then the average number per animal were provided.

### Hematoxylin and eosin staining and analysis

Hematoxylin and eosin staining was performed following the manufacture’s instruction (Abcam, ab245880). We prepared six 14 μm non-consecutive retinal cryosections per animal, and we measured thickness of all retinal layers, inner nuclear layer (INL) and outer nuclear layer (ONL) at two middle regions of each retinal section, and the average was provided per animal. Bright field microscope (Olympus) was used for imaging.

### Analysis of regenerated axons

Methods of clearing CTB-labeled optic nerves have been previously described^1^ based on reagents used in *Clear*^*T*^^105^ and Sca*l*eS^106^. CTB-labeled regenerated retinal axons were imaged on the confocal microscope with a 20x objective lens, and ~50 images with 3 μm steps were provided per optic nerve. Each image was stitched to generate the whole optic nerve image. For counting axon number from the lesion site, a line transecting at every 400 or 1000 μm from the lesion site was drawn in the merged images, and the total number of the CTB-labeled axons crossing the transection line was provided per animal.

### Statistical analysis

Both male and female adult mice were randomly used in each sample group because this study was not intended to test for differences between males and females. Animals were randomized to experimental groups. For experimental analysis, one investigator was responsible for animal surgery and injections and sent images or gave dissociated samples with only codes (#1, #2, #3..) to other investigators, who counted RGCs, regenerated axons, and/or immune cells, and performed flow cytometric analysis or qPCR. For bulk RNA-seq analysis, details of experimental group were provided before the analysis to avoid analytical mistakes. Although no statistical methods were used to predetermine sample sizes, the sample size was determined based on our and other studies in the field. All data were analyzed, and graphs were constructed using Fiji (version 1.0) and Microsoft Excel. All error bars represent the standard deviation (SD), and statistical analysis was determined using four different ways depending on the number of groups and one or more than two factors such as different retinal regions or locations from the lesion site within the nerve. Unpaired two-tailed Student’s t-test was used when comparing two groups of data. One-way ANOVA followed by Tukey’s post hoc test was used when comparing more than three groups of data. In quantification data on RGC survival in different retinal areas (VT, DT, DN, VN) or the number of regenerated axons at different locations from the lesion site within the nerve, two-way ANOVA followed by Tukey’s post hoc test was used when comparing more than three groups of data and two-way ANOVA followed by Bonferroni’s test was used when comparing two groups of data. These were indicated in the figure legends associated with each figure. A *p*-value < 0.05 was considered significant. All statistical tests were performed using GraphPad Prism 7. The number of animals used in each experiment was also indicated in each plot in the figures.

## Supplementary information

Supplementary Information

## Data Availability

All data generated or analyzed during this study are included in this article and its supplementary information files. Raw RNA-seq data are available at National Center for Biotechnology Information (NCBI) Gene Expression Omnibus (GEO) Data Sets: GSE226780.

## Code availability

No custom code was used in the collection of data. Fluorescence imaging was performed with Olympus Fluoview 1000 laser scanning microscope with Fluoview software. Hematoxylin and eosin staining imaging was performed with bright field microscope (Olympus). qRT- PCR was performed in duplicate using iTaq Universal SYBR Green (BioRad, 1725121) and the CFX384 Touch Real-Time PCR Detection System (BioRad). RNA-seq data was collected with NextSeq500 (Illumina). Analysis of fluorescence signal intensity and counting cells were performed with Fiji (version 1.0). All statistical tests were performed using GraphPad Prism 7. FASTQ files by RNA-seq were analyzed using CLC Genomics Grid Workbench v22.0.2. Read counts were generated and differentially expressed genes were obtained using CLC Genomic Workbench v22.0.2. Heatmaps and volcano plots were generated using CLC Genomic Workbench v23.0.1 and GraphPad – Prism 9.0. Venn diagram online analysis provided by VIB/UGent Bioinformatics & Evolutionary Genomics was used. (https://bioinformatics.psb.ugent.be/webtools/Venn/).
