# Supplementary material for: Immunomodulation by the combination of statin and matrix-bound nanovesicle enhances optic nerve regeneration
Source: NPJ Regen Med. 2024 Oct 26;9:31. doi: 10.1038/s41536-024-00374-y (PMC11513974; doi:10.1038/s41536-024-00374-y)
Supplement: Supplementary file 1 — Supplementary Information [file 41536_2024_374_MOESM1_ESM.pdf]

**Supplementary Information**

# Immunomodulation by the combination of statin and Matrix-Bound Nanovesicle enhances optic nerve regeneration

Gregory P. Campbell[1, 2], Dwarkesh Amin[1, 2], Kristin Hsieh[1, 2], George S. Hussey[3, 4], Anthony J. St. Leger[1, 5], Jeffrey M. Gross[1, 2, 4, 6], Stephen F. Badylak[3, 4, 7, 8] and Takaaki Kuwajima[1, 2, *]

[1] Department of Ophthalmology, University of Pittsburgh School of Medicine, Pittsburgh, PA 15219, United States.

[2] The Louis J. Fox Center for Vision Restoration, University of Pittsburgh School of Medicine, Pittsburgh, PA 15219, United States.

[3] Department of Pathology, University of Pittsburgh, Pittsburgh, PA 15219, United States.

[4] McGowan Institute for Regenerative Medicine, University of Pittsburgh, Pittsburgh, PA 15219, United States.

[5] Department of Immunology, University of Pittsburgh, Pittsburgh, PA 15213, United States.

[6] Department of Developmental Biology, University of Pittsburgh School of Medicine, Pittsburgh, PA 15213, United States.

[7] Department of Surgery, University of Pittsburgh, Pittsburgh, PA 15219, United States.

[8] Department of Bioengineering, University of Pittsburgh, Pittsburgh, PA 15219, United States.

* Correspondence:
Takaaki Kuwajima, PhD
Email: kuwajima@pitt.edu

**This PDF file includes:**

Supplementary Figure 1-7
Supplementary Table 1-4

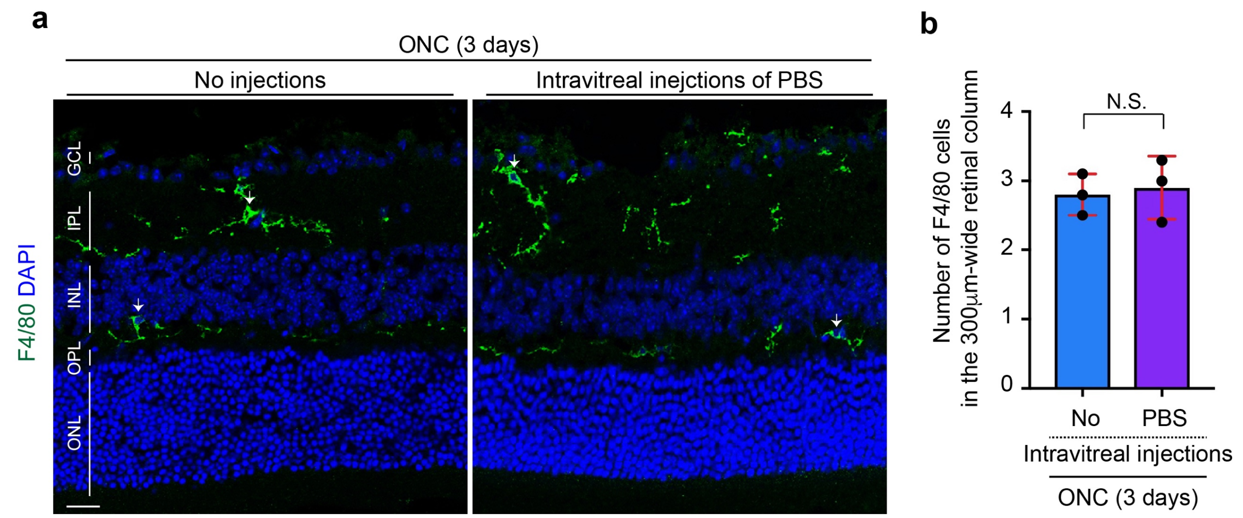


**Supplementary Figure 1: Analysis of the number of macrophages after intravitreal injections twice after ONC**

**a** Images of macrophages (F4/80$^+$ cells) in the injured retina three days after ONC following intravitreal injections of PBS twice after ONC or without any injections. **b** Quantitative analysis of macrophages (F4/80$^+$ cells) in a 300 μm (width) area of the retina. (n = 3, N.S., two-tailed unpaired t-test). Data presented as mean ± SD. N.S., not significant; Scale bar represents 20 μm

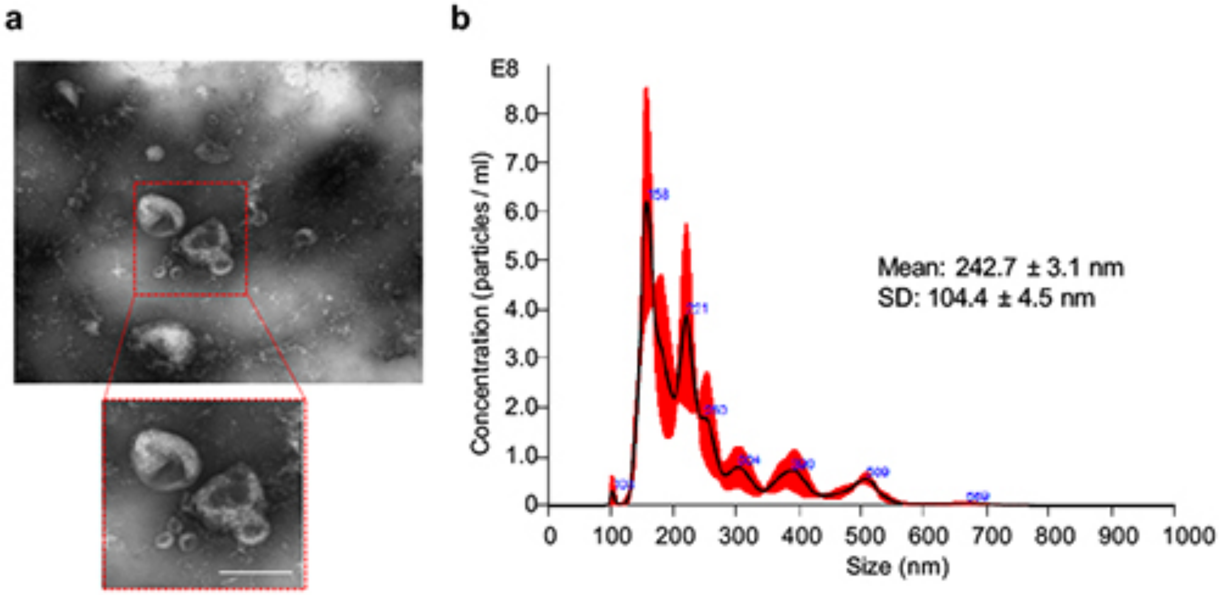


**Supplementary Figure 2: Size and concentrations of MBV**

**a** Representative image of MBV with transmission electron microscopy. **b** The size and concentrations of MBV by Nanosight nanoparticle tracking analysis. Scale bar represents 200 nm

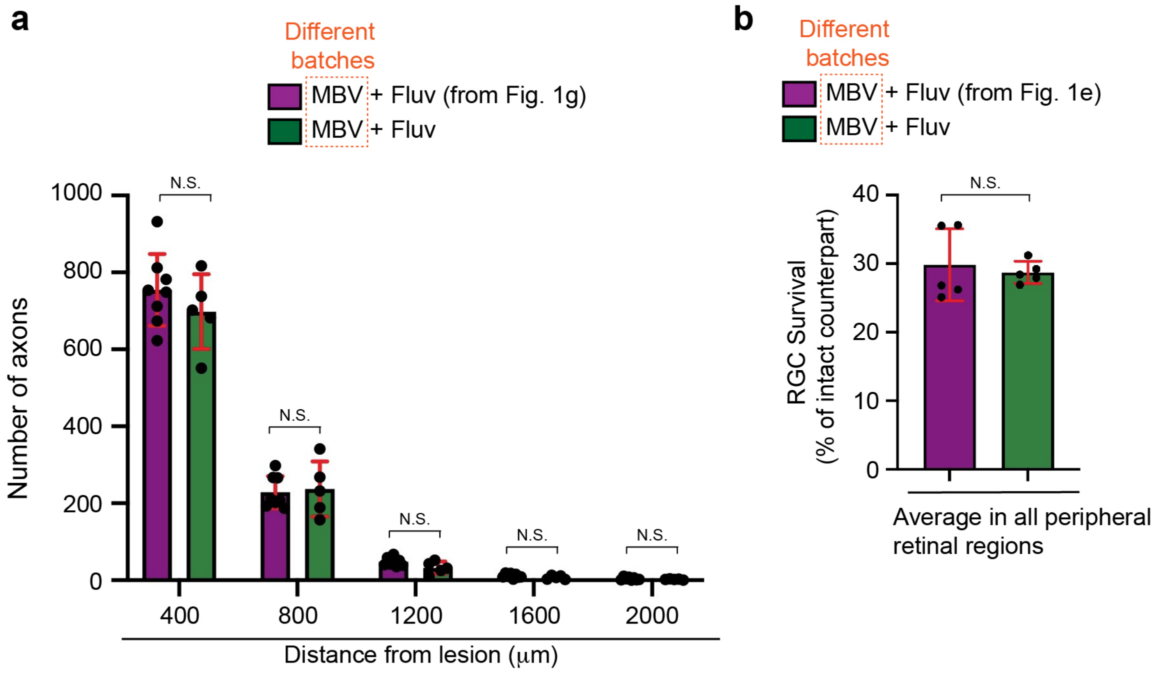


**Supplementary Figure 3: The same magnitude of axon regeneration and RGC protection by different batches of MBV with fluvastatin after ONC.**
**a** Quantitative analysis of the number of regenerated axons at 400 – 2000 μm from the lesion sites in the injured retina treated with different batches of MBV (2.3 x $10^9$ particles/ml) with fluvastatin (1.3 μg/μl) two weeks after ONC. (n = 5 - 8, two-way ANOVA with Bonferroni's test). **b** Quantitative analysis of RBPMS$^+$ RGC survival (%) in the average of RGC survival from all four regions in the peripheral retina treated with different batches of MBV with fluvastatin two weeks after ONC. (n = 5, two-tailed unpaired t-test). Data presented as mean ± SD. N.S., not significant.

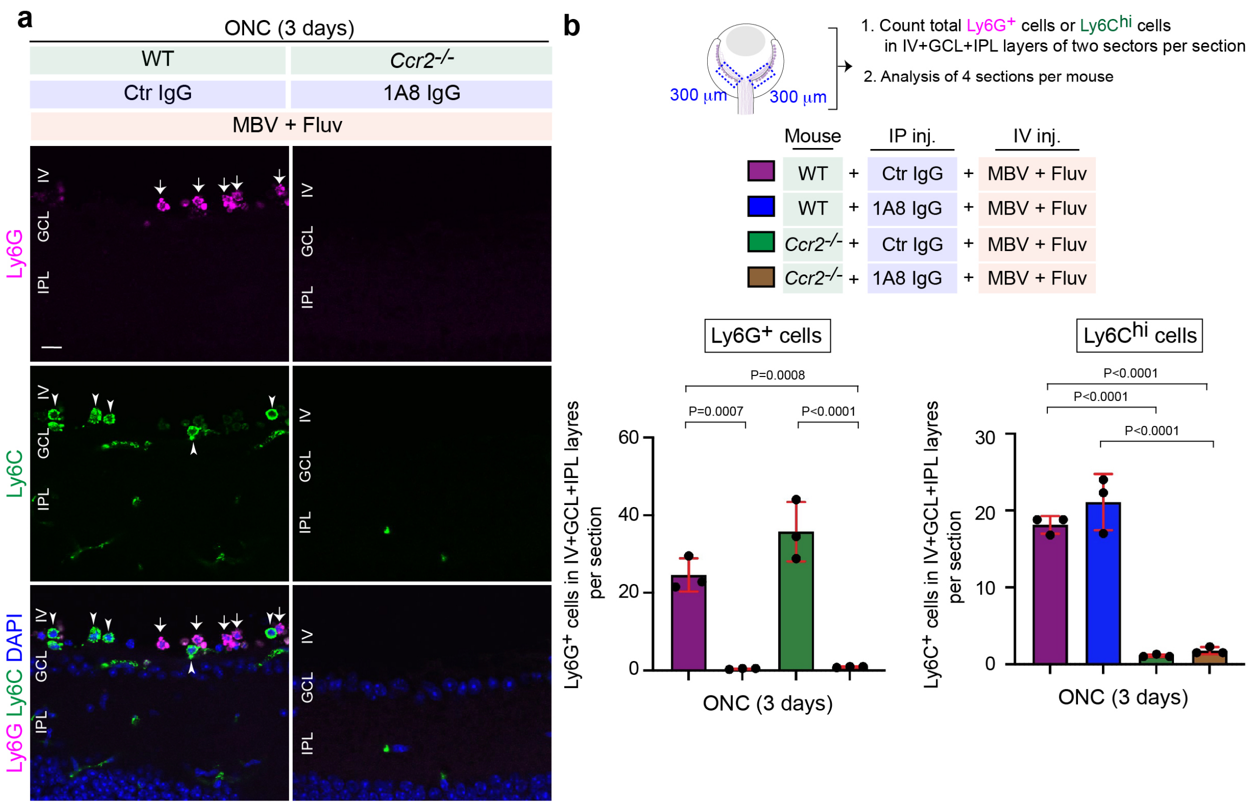


**Supplementary Figure 4: Depletion of neutrophils and monocytes in the injured retina treated with MBV and fluvastatin.**

**a** Images of neutrophils (Ly6G$^{+}$) (arrows) and monocytes (Ly6C$^{hi}$) (arrowheads) in the retina treated with the combination of MBV and fluvastatin in WT mouse treated with control IgG or in *Ccr2$^{-/-}$* mouse with 1A8 antibody. **b** Quantitative analysis of neutrophils (Ly6G$^{+}$) and monocytes (Ly6C$^{hi}$) in the retina treated with the combination of MBV and fluvastatin three days after ONC in WT or *Ccr2$^{-/-}$* mouse treated with multiple intraperitoneal injections of control IgG or 1A8 antibody. Total number of those cells from two 300 µm (width) retinal areas (IV, GCL, and IPL) adjacent to the nerve head per section as shown in the cartoon were counted each condition, and the average from four sections per mouse was provided. (n = 3, one-way ANOVA with Tukey's post-hoc). Data presented as mean ± SD; Scale bar represents 20 µm.

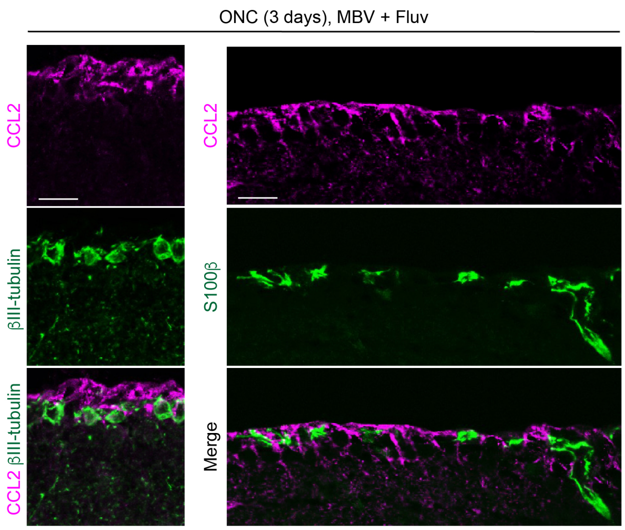


**Supplementary Figure 5: Analysis of CCL2 expression in RGCs and astrocytes of the injured retina treated with MBV and fluvastatin after ONC**

Images of RGCs (Tuj1$^{+}$ cells) and astrocytes (S100B$^{+}$ cells) three days after ONC following intravitreal injections of MBV and fluvastatin post-injury. Note that CCL2 expression is not detected in RGCs nor astrocytes. Scale bars represent 20 μm.

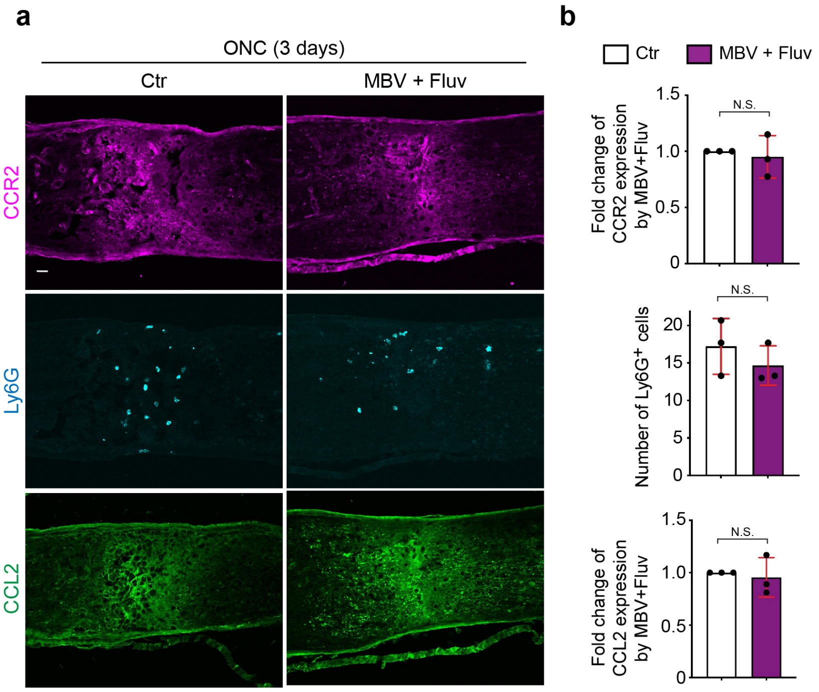


**Supplementary Figure 6: Expression of CCR2 and CCL2 and the number of Ly6G$^{+}$ cells at the lesion site of the optic nerve treated with MBV and fluvastatin or PBS after ONC**

**a** Images of expression of CCR2 and CCL2 and neutrophils (Ly6G$^{+}$ cells) at the lesion site of the optic nerve three days after ONC following intravitreal injections of MBV and fluvastatin or PBS (Ctr). **b** Quantitative analysis of CCR2 and CCL2 expression levels and the number of neutrophils (Ly6G$^{+}$) at the lesion site three days after ONC following intravitreal injections of MBV and fluvastatin or PBS (Ctr). (n = 3, two-tailed unpaired t-test). Data presented as mean ± SD; Scale bar represents 20 μm.

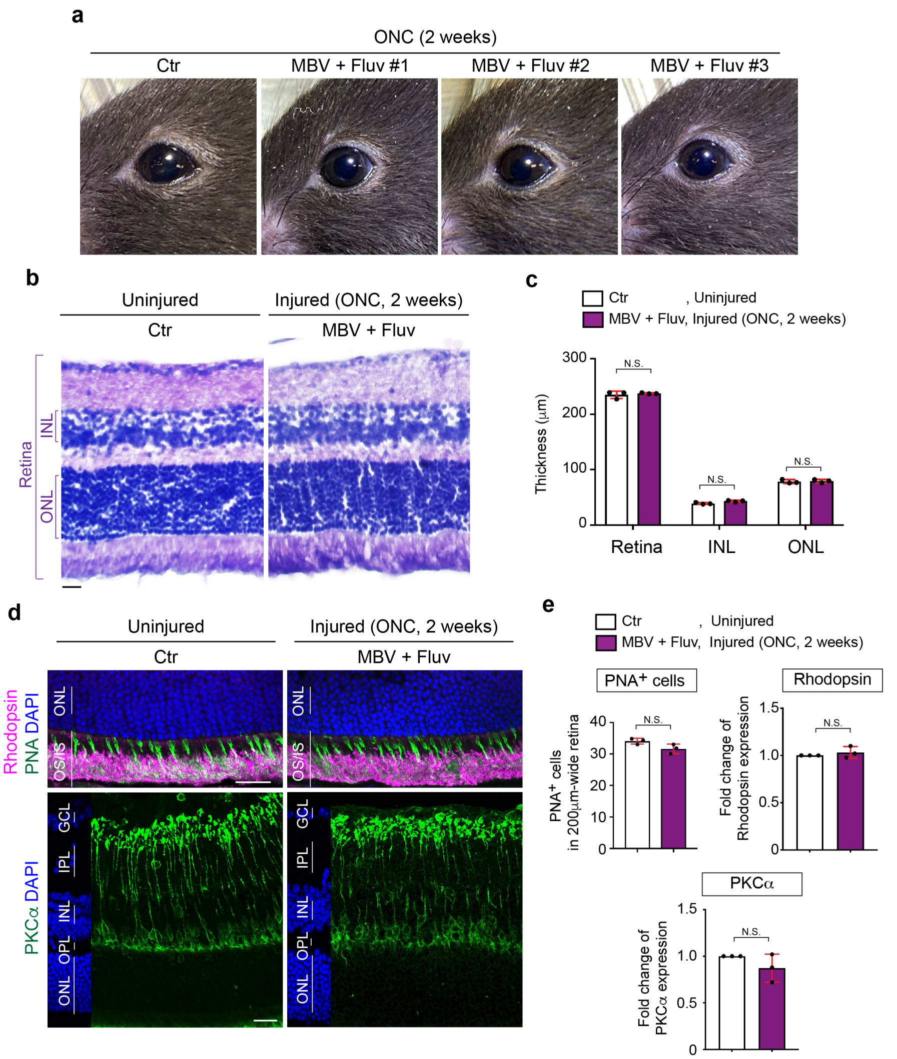


**Supplementary Figure 7: Analysis of the effects of MBV and fluvastatin on other retinal cells.** **a** No cataract induction by MBV and fluvastatin or PBS (Ctr) two weeks after ONC. **b, c** Hematoxylin and eosin staining and analysis in the uninjured retina treated with PBS (Ctr) and injured retina treated with MBV and fluvastatin two weeks after ONC (*b*) and analysis of thickness of the whole retina, inner nuclear layer (INL), and outer nuclear layer (ONL) (*c*) (n = 3, two-tailed unpaired t-test at each layer). **d, e** Images of photoreceptors (rod: Rhodopsin; cone: PNA) and bipolar cells (PKCα) in the uninjured retina treated with PBS (Ctr) and injured retina treated with MBV and fluvastatin two weeks after ONC (*d*) and analysis of the number of $PNA^+$ cells and expression of Rhodopsin and PKCα (n = 3, two-tailed unpaired t-test). Data presented as mean ± SD; Scale bars represent 20 μm.

**Supplementary Table 1: Common and different top DEGs by the single and combined treatments.**

A list of common and different DEGs with an FDR p-value < 0.05 and a fold-change of at least 2 (referred to as top DEGs) in the injured retina treated with either MBV or fluvastatin alone, or the combination of MBV and fluvastatin two days after ONC when compared to PBS (CTR).

| Treatment | Common DEG Name | Total Common DEG # |
|---|---|---|
| Fluv vs CTR<br>MBV vs CTR<br>MBV+Fluv vs CTR | Dcn, Fosl1, Cryba1, Ccl2, Glycam1, Tyrp1, Pmel, Mlana, Adamts19, Prss56, Tmem140, Sh3rf2, Gm21985, Dct, Cxcl10 | 15 |
| Fluv vs CTR<br>MBV vs CTR | Gm14744, Gstm2, H2ac12, Pycr1, Islr, Gsta3, Fam177a2, Prrx1, Col4a3, Obp1a, Olfml2a, Tmem26, Tph1, Tfcp2l1, Rps27rt, Ermap, Akr1c14, Slc22a12, Vcan, Niban1, Ndufa4l2, Slc16a12, Pabpc4l, Scn4b, Gm8797, Gm49388, Bpifa1, Prr18, Lif, Anxa8, Hmga1b, Ogn, Ucn2, Gm10358, Lad1, Shisal2b, Aplnr, Gm12185, Dsg1a, Suclg2, 5430402E10Rik, Zic1, Gm1604b, Serpine3, Tnnt2, Gja1, P4ha3, Lars2, Nupr1, Ccl3, Plscr2, Tbx22, Stac3, Ltf, Six1, Id3, Exoc3l4, Npsr1, Matn3, Nkd2, Rapsn, Cped1, 4933405O20Rik, Klhdc7b, Efemp1, Papss2, Aass, Ugt1a6a, Muc3a, Zfp185, Ch25h, Obp2b, Aldh1a2, Pvalb, Emp1, Cdh19, Gm27179, Hmga2, Lum, Maob, Mlph, Musk, Mdfic2, Veph1, Ccl21b, Mrgprf, Pmp2, Nes, Gm3839, Yy2, Gm21897, Nkd1, Srp54b, Obp2a, Glb1l3, Fbln5, Mgst1, Vxn, Mme, Myrf, Colec12, Ptgfr, Gm14434, Robo4, Eno1b, Gpx3, Hba-a2, Slc7a10, Hdc, Olfr769, Gm21962, Kcng1, Ppp1r14a, Gpnmb, Gm21833, Mup4, Kif4, Olfr1031, Gm14698, Folr1, Prrg1, Obp1b, Gm10184, Tnfsf10, Gm20721, Tyr, Tph2, Cstdc5, Slc26a4 | 129 |
| Fluv vs CTR<br>MBV+Fluv vs CTR | H2bc24, Igf2bp1, Rsad2, Chil1 | 4 |
| MBV vs CTR<br>MBV+Fluv vs CTR | Cd74, Cfb, H2-Ab1, Cebpd, Slfn5, Tlr2, Pgf, Ccl5, Nr4a3, C3, Baz1a, Mndal, Arhgap25, Ifi211, F10, Fcgr1, Ccr2, Hsd17b2, Slfn4, Aoah, Ifi207, Cybb, Slc15a3, Oas1a, Myo1f, H2-Eb1, Plbd1, H2-Aa, Crygs, Cebpa, Il6ra, Ror2, Ifi203, Syk, Crybb2, Csf2rb2, Gvin2, Pagr1a, H2-Q7, Siglec1, | 40 |

| | | |
|---|---|---|
| Fluv vs CTR | Fap, Ly75, Tap1, Pde10a, Cbr2, Acss3, Fbxw21, Ifit1bl1, H2bc23, Npcd, 2610528A11Rik, Ifitm1, Thbs4, Slc7a2, Gm21244, Nefh, Mrc2, Id1, Rgr, Bmp2, Chst5, Cyp2a5, Gm50367, Car12, Ntf3, Cdh3, Etnppl, Gm10800, Sfrp1, Nog, Gcat_2, Tcim, Reg3g, Folh1, Tpm3-rs7, Crispld1, Rpl9-ps6, Sap25, Ube2t, Upk3b, Pate2, Agbl2, Adh1, Gng11, Pdlim1, Hbb-bs, 1700007K13Rik, Isyna1, Hbbbt, Emp3, Spag4, Nat8f6, Ttr, Irx1, Hhip, Vmn2r6, Mdfic, Gstt3, Slc16a8, Plekhg6, Edn3, Gm9803, Gm4767, Rasef, Slc6a20a, Msr1, Gm13304, Tgm1, Fam107a | 69 |
| MBV vs CTR | Ehf, Rtp3, Angptl4, Knl1, Mcam, Zfp979, Ip6k3, Vmn2r54, Lmntd2, Maff, Cryaa, Olfm4, Taf4b, Flnc, Bmp4, Arid3c, Zfp972, Apobr, Ttll11, L3mbtl1, Sod3, Pik3r6, Sh2d1a, Alpk2, Gadd45b, H2-Q5, Cndp1, Mip, Lgals12, Krt6b, Synpo, Fbln7, Crygc, Gm715, Tagln2, Mfap4, Fat2, Agmo, Stap2, Dlx3, Depp1, Vmn2r7, Fam205a3, Slc47a1, Cryga, Krt5, Unc13d, Paqr6, Ripply3, Fn1, Ect2, Kif19b, A3galt2, Nxph4, Grifin, Ybx2, Cspg4b, Chek2, Foxd2, Slc39a12, Nid1, Plac8, Pld5, Olfr552 Plb1, Fam71d, Ccl21d, 2410004P03Rik, Gm12184, Myc, Vmn2r30, Gsc2, Kif19a, Cdsn, A830005F24Rik, Litaf, Loxl4, Mog, Crybb1, Olfr455, Klhdc7a, Crygf, Slc22a28, Cxcl2, Sytl1, Col6a1, 4930590J08Rik, Il2rg, 2810459M11Rik, AU021092, Mapk15, Msx1, Thbd, Sned1, Rassf10, Slc22a29, Ccdc68, Gm45337, Nkx6-2, Tmprss5, Aldh3a1, Col4a4, Dleu7, Ccdc188, Serpinb9, Acoxl, Lefty1, Kcnmb1, Ap1g2, Pdzd7, Krt13, Cryge, Zbp1, Crygd, Misp, Lgsn, Lax1, Amd2, Krt12, Ccdc187, Piwil4, Krt6a, Kcne1l, Gm10591, Gm44505, Muc4, Adamts4, Creb3l1, Col19a1, Matn2, Clec18a, Crhbp, Gm37389, Epha2, Vmn2r118, Cdcp3, Gm10282, Itgal, F5, Gm28635, Atp1a2, Ackr3, Gja8, Krt15, Zpbp, Creb3l3, Spsb1, Rnf43, Col3a1, Sox30, Col5a3, Hrh1, Oas3, Cryba2, Aldh1a3, Cgas, Spc25, Tmem252, Fam167a, Klhl14, Hrc, Gm45861, | 220 |

| | | |
|---|---|---|
| | Fbxo47, Bfsp1, Col6a6, Rassf9, Fxyd5, Eif4ebp1, Aoc3, Sp100, Heph, Col7a1, Prtg, Gm20075, Mmp23, Map3k6, Adgrg6, Atf5, Bace2, 5730507C01Rik, Otx1, Myh11, Arl13a, Mfrp, Tedc1, Cilp, Kcp, Eps8l1, Col24a1, Adora2a, Bcl3, Ltbp2, Slc25a21, Des, Egr2, Tbx6, Cryba4, Cox8b, Nuf2, 5530401A14Rik, Slc13a4, Foxi3, Defb11, Sostdc1, Crybb3, Galnt6, 4930523C07Rik, Gm6588, Gimap6, Draxin, Abcc6, Slc26a10, Obscn, Ecrg4, Jak3, Arhgef16, Rfx4, Crygb, Penk, Gm10719 | |
| MBV+Fluv vs CTR | P2ry6, Ccl6, C1ra, Treml2, Tyrobp, Apoc2, Slamf9, Dpep2_1, Capg, Egr3, Cd48, Napsa, B430306N03Rik, Sla, Tlr13, Ctla2a, Pirb, Gpr141, Clec4a3, Gpr65, C1s1, Ms4a4c, Fcgr2b, Neurl3, Cd52, Cd300lb, Ifi209, Krt8, Ptpn6, Ptafr, Trim30a, Gm20431, Csf3r, Ccr5, Cables1, Cytip, Csf2rb, Ifi213, H2ac19, Fcgr4, Icam1, Fyb, Ms4a7, Itgam, Tgfbi, Clec7a, Cd300c2, C3ar1, Ccl22, Cxcr2, Gda, Hck, Atp8b4, Ncf1, Spi1, Ctss, Adgrl4, Slfn8, Gm21188, Ms4a6c, Bcl2a1b, Gm49339, Fgr, Pmaip1, Anpep, Kcnk6, Lcn2, Runx3, Birc3, Cd209a, Gvin1, Ikzf1, Clec4n, Ms4a6d, Bpifb9a, Gal, Ly9, Arhgap30, Ncf4, Cd53, Slamf7, Phf11b, Cd300ld, C5ar1, Vav1, Itgax, Rac2, Itgb2, Nfam1, Clec5a, Apobec1, Bpifb9b, Serpine1, Bcl2a1d, Cmklr1, Depdc7, Ccr1, H2-DMb1, Themis2, Ly6c2, Saa3, Fcer1g, Lcp2, Cysltr1, Steap4, Cyp4f18, Trim30d, Cd244a, Ifi204, Slfn1, Wdfy4, Il10ra, Ms4a6b, Card11, Epsti1, Gm28539, Clec12a, Adgre1, Mpeg1, Mx1, Lcp1, Was, Ptprc, Lyz2, Sirpb1c, Clec4a1, Acp5, Spn, Pira2, Slfn2, Tlr8, Cd84, Arg1 | 133 |

**Supplementary Table 2 - 4. Top DEGs by the single and combined treatments.**
DEGs with an FDR p-value < 0.05 and a fold-change of at least 2 (referred to as top DEGs) in the injured retina treated with either fluvastatin (Supplementary Table 2) or MBV (Supplementary Table 3) alone, or the combination of MBV and fluvastatin (Supplementary Table 4) two days after ONC when compared to PBS (CTR).

**Supplementary Table 2: Fluvastatin vs CTR**

| Name | Chromosome | Fold change | P-value | FDR p-value |
|---|---|---|---|---|
| Col4a3 | 1 | -3.11 | 6.21E-36 | 5.56E-32 |
| Pmel | 10 | -4.02 | 1.30E-32 | 7.75E-29 |
| Dct | 14 | -3.19 | 4.02E-25 | 1.44E-21 |
| Pde10a | 17 | 2.08 | 3.57E-24 | 1.07E-20 |
| Nefh | 11 | 2.1 | 1.17E-22 | 2.62E-19 |
| Gpnmb | 6 | -2.44 | 1.42E-20 | 2.12E-17 |
| Hdc | 2 | -3.45 | 4.74E-18 | 4.99E-15 |
| Lars2 | 9 | 9.13 | 3.18E-17 | 2.71E-14 |
| Gm14698 | X | -2.62 | 4.64E-17 | 3.78E-14 |
| Isyna1 | 8 | -2.14 | 2.92E-16 | 2.09E-13 |
| Olfr769 | 10 | 13 | 6.36E-16 | 4.37E-13 |
| Glb1l3 | 9 | -2.91 | 1.14E-14 | 6.37E-12 |
| Eno1b | 18 | -4.48 | 5.44E-12 | 1.60E-09 |
| Id3 | 4 | -2.59 | 6.76E-12 | 1.95E-09 |
| Obp1b | X | -19.3 | 1.80E-11 | 4.68E-09 |
| Gm20721 | 2 | -2.34 | 3.74E-11 | 8.59E-09 |
| Tyr | 7 | -3.38 | 5.93E-11 | 1.30E-08 |
| Adamts19 | 18 | 6.22 | 6.01E-11 | 1.30E-08 |
| Tmem26 | 10 | 2.66 | 7.07E-11 | 1.49E-08 |
| Thbs4 | 13 | 3.59 | 1.37E-10 | 2.62E-08 |
| Slc7a2 | 8 | -2.05 | 1.52E-10 | 2.84E-08 |
| Gm14744 | X | -27.1 | 2.71E-10 | 4.53E-08 |
| Obp1a | X | -25 | 4.65E-10 | 7.36E-08 |
| Prss56 | 1 | 15.1 | 6.79E-10 | 1.01E-07 |
| Tyrp1 | 4 | -3.87 | 9.27E-10 | 1.27E-07 |
| H2ac12 | 13 | -17.5 | 2.81E-09 | 3.35E-07 |
| Hba-a2 | 11 | 2.78 | 3.90E-09 | 4.31E-07 |
| Nes | 3 | 4.38 | 6.67E-09 | 6.86E-07 |
| Gm21897 | 16 | 4.4 | 8.23E-09 | 8.23E-07 |
| Obp2a | 2 | -51.8 | 9.59E-09 | 9.28E-07 |
| Shisal2b | 13 | -4.78 | 9.80E-09 | 9.38E-07 |
| Mlana | 19 | -5.37 | 1.39E-08 | 1.26E-06 |
| Myrf | 19 | 2.21 | 1.52E-08 | 1.36E-06 |

| | | | | |
|---|---|---|---|---|
| Mup4 | 4 | -56 | 1.92E-08 | 1.65E-06 |
| Nkd1 | 8 | 2.54 | 3.66E-08 | 2.83E-06 |
| 5430402E10Rik | X | -31.2 | 3.93E-08 | 3.00E-06 |
| Serpine3 | 14 | 6.81 | 4.40E-08 | 3.31E-06 |
| Gng11 | 6 | -2.14 | 4.70E-08 | 3.49E-06 |
| Efemp1 | 11 | -2.23 | 6.18E-08 | 4.35E-06 |
| Ly75 | 2 | -2.16 | 7.46E-08 | 5.17E-06 |
| Bpifa1 | 2 | -18.6 | 8.79E-08 | 5.85E-06 |
| Gm3839 | 14 | -3.43 | 9.58E-08 | 6.32E-06 |
| Hbb-bs | 7 | 2.19 | 9.92E-08 | 6.52E-06 |
| Kif4 | X | -2.04 | 1.04E-07 | 6.72E-06 |
| Ttr | 18 | 2.23 | 1.15E-07 | 7.37E-06 |
| Islr | 9 | -2.95 | 1.37E-07 | 8.37E-06 |
| Gm9803 | 10 | -14.7 | 1.42E-07 | 8.64E-06 |
| Etnppl | 3 | -3.24 | 1.58E-07 | 9.45E-06 |
| Tnnt2 | 1 | 3.04 | 1.64E-07 | 9.73E-06 |
| Mdfic | 6 | -2.04 | 4.37E-07 | 2.20E-05 |
| Suclg2 | 6 | -2.14 | 4.37E-07 | 2.20E-05 |
| Gm21962 | 3 | 3.7 | 5.53E-07 | 2.68E-05 |
| Rps27rt | 9 | 4.28 | 5.96E-07 | 2.85E-05 |
| Folh1 | 7 | -2.49 | 7.82E-07 | 3.54E-05 |
| Akr1c14 | 13 | -7.02 | 8.04E-07 | 3.63E-05 |
| Gsta3 | 1 | -2.61 | 9.59E-07 | 4.20E-05 |
| Gm21985 | 2 | 3.51 | 1.06E-06 | 4.59E-05 |
| Gm1604b | 17 | 4.93 | 1.08E-06 | 4.63E-05 |
| Aass | 6 | -3.08 | 1.16E-06 | 4.93E-05 |
| Mgst1 | 6 | -2.6 | 1.26E-06 | 5.26E-05 |
| Zic1 | 9 | -2.34 | 1.41E-06 | 5.81E-05 |
| Rgr | 14 | 2.08 | 1.63E-06 | 6.55E-05 |
| Ppp1r14a | 7 | 2.49 | 2.11E-06 | 8.20E-05 |
| Acss3 | 10 | -2.17 | 2.42E-06 | 9.25E-05 |
| Cped1 | 6 | -2.39 | 2.86E-06 | 1.06E-04 |
| Prrx1 | 1 | -2.88 | 3.10E-06 | 1.12E-04 |
| Maob | X | -2.41 | 3.47E-06 | 1.23E-04 |
| Papss2 | 19 | -2.04 | 4.62E-06 | 1.57E-04 |
| Gm49388 | 7 | 1183.3 | 5.81E-06 | 1.89E-04 |
| Pvalb | 15 | 2.24 | 6.11E-06 | 1.96E-04 |
| Gm14434 | 2 | -15.1 | 6.19E-06 | 1.98E-04 |
| Cdh3 | 8 | 2.24 | 6.53E-06 | 2.06E-04 |
| Dcn | 10 | -4.4 | 9.06E-06 | 2.70E-04 |

| | | | | |
|---|---|---|---|---|
| Rpl9-ps6 | 19 | -3.36 | 9.27E-06 | 2.76E-04 |
| Gm10184 | 17 | -7.55 | 1.84E-05 | 4.81E-04 |
| Pycr1 | 11 | 2.09 | 2.08E-05 | 5.31E-04 |
| H2bc24 | 13 | 3.77 | 2.44E-05 | 6.01E-04 |
| Ermap | 4 | 3.53 | 3.25E-05 | 7.54E-04 |
| Olfr1031 | 2 | 43.3 | 3.35E-05 | 7.73E-04 |
| Sh3rf2 | 18 | -5.37 | 3.66E-05 | 8.35E-04 |
| Rapsn | 2 | -3.4 | 4.28E-05 | 9.53E-04 |
| Slc16a8 | 15 | 2.26 | 4.40E-05 | 9.73E-04 |
| Upk3b | 5 | 4.4 | 4.51E-05 | 9.89E-04 |
| Ptgfr | 3 | 4.38 | 4.79E-05 | 1.04E-03 |
| Scn4b | 9 | 2.88 | 5.43E-05 | 1.14E-03 |
| Sfrp1 | 8 | -2.04 | 5.46E-05 | 1.15E-03 |
| Npsr1 | 9 | -2.8 | 6.68E-05 | 1.33E-03 |
| Cxcl10 | 5 | 6.55 | 7.45E-05 | 1.46E-03 |
| Gm10800 | 2 | -2.12 | 7.57E-05 | 1.48E-03 |
| Prrg1 | X | -2.33 | 7.68E-05 | 1.50E-03 |
| Cyp2a5 | 7 | -8.96 | 8.01E-05 | 1.55E-03 |
| Tmem140 | 6 | 3.09 | 8.13E-05 | 1.57E-03 |
| Mlph | 1 | -2.94 | 8.86E-05 | 1.68E-03 |
| Slc6a20a | 9 | 2.02 | 9.62E-05 | 1.79E-03 |
| Crispld1 | 1 | -2.12 | 9.83E-05 | 1.82E-03 |
| Slc26a4 | 12 | -10.9 | 1.09E-04 | 1.96E-03 |
| Ugt1a6a | 1 | -2.68 | 1.10E-04 | 1.98E-03 |
| Hmga1b | 11 | -3.39 | 1.25E-04 | 2.20E-03 |
| Sap25 | 5 | 3.22 | 1.30E-04 | 2.26E-03 |
| Tbx22 | X | -3.64 | 1.40E-04 | 2.38E-03 |
| Gm12185 | 11 | -9.42 | 1.56E-04 | 2.60E-03 |
| Gstt3 | 10 | -2.04 | 1.83E-04 | 2.96E-03 |
| Obp2b | 2 | -22.8 | 1.90E-04 | 3.05E-03 |
| Lum | 10 | -5.92 | 1.98E-04 | 3.14E-03 |
| Npcd | 15 | -2.54 | 2.21E-04 | 3.44E-03 |
| Exoc3l4 | 12 | -3.45 | 2.25E-04 | 3.48E-03 |
| Id1 | 2 | -2.1 | 2.33E-04 | 3.59E-03 |
| Vxn | 1 | -2.38 | 2.37E-04 | 3.64E-03 |
| Lad1 | 1 | 2.54 | 2.38E-04 | 3.65E-03 |
| H2bc23 | 13 | -2.38 | 2.57E-04 | 3.86E-03 |
| Plscr2 | 9 | -2.59 | 2.89E-04 | 4.24E-03 |
| Car12 | 9 | 2.06 | 3.07E-04 | 4.41E-03 |
| Cdh19 | 1 | -2.32 | 3.11E-04 | 4.46E-03 |

| | | | | |
|---|---|---|---|---|
| Gm8797 | 3 | -2.25 | 3.21E-04 | 4.58E-03 |
| Six1 | 12 | -2.24 | 3.24E-04 | 4.62E-03 |
| Musk | 4 | -6.35 | 3.34E-04 | 4.73E-03 |
| Tph2 | 10 | -2.07 | 3.40E-04 | 4.80E-03 |
| Cryba1 | 11 | 4.51 | 3.41E-04 | 4.82E-03 |
| Igf2bp1 | 11 | -4.1 | 3.74E-04 | 5.21E-03 |
| Niban1 | 1 | -2.07 | 3.97E-04 | 5.46E-03 |
| Slc16a12 | 19 | -3.82 | 4.01E-04 | 5.49E-03 |
| Klhdc7b | 15 | 2.6 | 4.40E-04 | 5.90E-03 |
| Adh1 | 3 | -4.55 | 5.10E-04 | 6.63E-03 |
| Robo4 | 9 | 2.11 | 5.39E-04 | 6.92E-03 |
| Folr1 | 7 | -2.37 | 5.46E-04 | 7.00E-03 |
| Mme | 3 | -2.57 | 5.97E-04 | 7.53E-03 |
| Tap1 | 17 | -2.07 | 5.99E-04 | 7.54E-03 |
| Nupr1 | 7 | 2.36 | 6.47E-04 | 7.95E-03 |
| Ifitm1 | 7 | -3.55 | 6.49E-04 | 7.97E-03 |
| Tcim | 8 | -2.41 | 7.00E-04 | 8.43E-03 |
| Vcan | 13 | -2.94 | 7.25E-04 | 8.64E-03 |
| Gm21833 | 16 | -7.01 | 7.30E-04 | 8.69E-03 |
| Slc7a10 | 7 | 2.73 | 7.35E-04 | 8.74E-03 |
| Fam107a | 14 | -2.37 | 7.52E-04 | 8.90E-03 |
| Anxa8 | 14 | -7.01 | 7.98E-04 | 9.33E-03 |
| Ltf | 9 | -6.46 | 8.56E-04 | 9.82E-03 |
| Pabpc4l | 3 | -2.91 | 9.55E-04 | 0.010688541 |
| Emp1 | 6 | -2.23 | 1.03E-03 | 0.01134821 |
| Pdlim1 | 19 | 2.12 | 1.04E-03 | 0.011435214 |
| Nog | 11 | 2.91 | 1.05E-03 | 0.01150388 |
| Tfcp2l1 | 1 | -2.16 | 1.09E-03 | 0.011822139 |
| Tgm1 | 14 | -2.36 | 1.11E-03 | 0.011921468 |
| Chil1 | 1 | -2.35 | 1.13E-03 | 0.012080289 |
| Gpx3 | 11 | -5.19 | 1.15E-03 | 0.012303514 |
| Edn3 | 2 | -3.92 | 1.22E-03 | 0.012854898 |
| Fbln5 | 12 | -2.04 | 1.23E-03 | 0.012904349 |
| Hhip | 8 | -2.07 | 1.29E-03 | 0.01339676 |
| Glycam1 | 15 | -4.05 | 1.29E-03 | 0.01339676 |
| Stac3 | 10 | 2.01 | 1.32E-03 | 0.013576013 |
| Lif | 11 | 3.06 | 1.34E-03 | 0.013758601 |
| Pate2 | 9 | -2.1 | 1.36E-03 | 0.013893999 |
| Ndufa4l2 | 10 | 2.16 | 1.38E-03 | 0.014042558 |
| Mrgprf | 7 | -3.22 | 1.42E-03 | 0.014335187 |

| | | | | |
|---|---|---|---|---|
| Rasef | 4 | -2.01 | 1.43E-03 | 0.014368995 |
| Reg3g | 6 | -12.1 | 1.45E-03 | 0.014517606 |
| Pmp2 | 3 | -2.15 | 1.52E-03 | 0.015075579 |
| Kcng1 | 2 | 3.46 | 1.52E-03 | 0.015114314 |
| Fam177a2 | 12 | 11.2 | 1.59E-03 | 0.015618526 |
| Veph1 | 3 | -2.88 | 1.64E-03 | 0.01598286 |
| Gja1 | 10 | -2.9 | 1.64E-03 | 0.01598286 |
| Fbxw21 | 9 | -2.41 | 1.72E-03 | 0.016573798 |
| Tnfsf10 | 3 | -2.23 | 1.75E-03 | 0.016844577 |
| Hbb-bt | 7 | 2.85 | 1.78E-03 | 0.017031082 |
| Vmn2r6 | 3 | -9.66 | 1.84E-03 | 0.017449097 |
| Gstm2 | 3 | -3.26 | 2.07E-03 | 0.019020586 |
| Gm21244 | Y | -3.39 | 2.18E-03 | 0.019757129 |
| Gm27179 | 14 | 132.3 | 2.23E-03 | 0.020167172 |
| Colec12 | 18 | -2.77 | 2.24E-03 | 0.020200317 |
| Ntf3 | 6 | -2.99 | 2.29E-03 | 0.020557038 |
| Msr1 | 8 | -3.18 | 2.30E-03 | 0.020578277 |
| Bmp2 | 2 | -2.83 | 2.31E-03 | 0.020684262 |
| Zfp185 | X | -2.51 | 2.32E-03 | 0.020684262 |
| Plekhg6 | 6 | -2.23 | 2.37E-03 | 0.020995425 |
| P4ha3 | 7 | 2.14 | 2.51E-03 | 0.021916967 |
| Slc22a12 | 19 | 4.97 | 2.52E-03 | 0.021987043 |
| Chst5 | 8 | -4.94 | 2.61E-03 | 0.022576649 |
| Gm10358 | 8 | -2.02 | 2.62E-03 | 0.022612753 |
| 1700007K13Rik | 2 | 2.16 | 2.66E-03 | 0.022845443 |
| Aplnr | 2 | 3.31 | 2.78E-03 | 0.023631479 |
| Matn3 | 12 | -4.28 | 2.79E-03 | 0.023679197 |
| Ogn | 13 | -3.38 | 3.02E-03 | 0.025137489 |
| Nkd2 | 13 | -2.05 | 3.30E-03 | 0.026990309 |
| Nat8f6 | 6 | -3.6 | 3.36E-03 | 0.02737347 |
| Ccl3 | 11 | 3.39 | 3.39E-03 | 0.027502439 |
| Aldh1a2 | 9 | -3.73 | 3.41E-03 | 0.027627538 |
| Prr18 | 17 | 2 | 3.77E-03 | 0.02992919 |
| Cstdc5 | 16 | -5.29 | 3.95E-03 | 0.031036732 |
| Spag4 | 2 | 2.42 | 3.98E-03 | 0.031210055 |
| Ccl21b | 4 | -18.9 | 4.03E-03 | 0.031519164 |
| Srp54b | 12 | -4.79 | 4.06E-03 | 0.031619827 |
| Cbr2 | 11 | -3.14 | 4.21E-03 | 0.032549563 |
| Olfml2a | 2 | -2.41 | 4.31E-03 | 0.033020551 |
| Rsad2 | 12 | 2.17 | 4.52E-03 | 0.034243931 |

| | | | | | |
|---|---|---|---|---|---|
| Fap | | 2 | 2.27 | 4.53E-03 | 0.034265808 |
| Ube2t | | 1 | -2.6 | 4.74E-03 | 0.035410071 |
| 2610528A11Rik | | 14 | 2.38 | 4.97E-03 | 0.036693303 |
| Mrc2 | | 11 | -2.03 | 5.24E-03 | 0.038154699 |
| Yy2 | X | | 2.25 | 5.29E-03 | 0.038409942 |
| Fosl1 | | 19 | 3.25 | 5.42E-03 | 0.039104937 |
| Gm50367 | | 17 | -3.38 | 5.48E-03 | 0.039403434 |
| Ccl2 | | 11 | 3.79 | 5.49E-03 | 0.039449492 |
| Dsg1a | | 18 | -5.02 | 5.55E-03 | 0.039761104 |
| Gm13304 | | 4 | 2.57 | 5.61E-03 | 0.040095234 |
| Muc3a | | 5 | 2.13 | 5.70E-03 | 0.040600838 |
| Tpm3-rs7 | | 14 | -2.83 | 5.72E-03 | 0.040677009 |
| Agbl2 | | 2 | -2.28 | 5.75E-03 | 0.040815289 |
| Irx1 | | 13 | 2.92 | 5.96E-03 | 0.041934746 |
| Gm4767 | | 10 | -3.08 | 6.08E-03 | 0.042551556 |
| Tph1 | | 7 | 2.08 | 6.15E-03 | 0.042920107 |
| Emp3 | | 7 | -2.05 | 6.23E-03 | 0.043340239 |
| Hmga2 | | 10 | 2.16 | 6.36E-03 | 0.043946269 |
| Ifit1bl1 | | 19 | -2.13 | 6.61E-03 | 0.045296728 |
| Ucn2 | | 9 | 2.6 | 6.86E-03 | 0.046574907 |
| 4933405O20Rik | | 7 | 2.76 | 6.89E-03 | 0.046729524 |
| Mdfic2 | | 6 | -2.06 | 7.16E-03 | 0.048008015 |
| Ch25h | | 19 | -2.79 | 7.21E-03 | 0.048175571 |
| Gcat_2 | | 15 | 3.36 | 7.28E-03 | 0.048546968 |

**Supplementary Table 3: MBV vs CTR**

| Name | Chromosome | Fold change | P-value | FDR p-value |
|---|---|---|---|---|
| Col4a3 | 1 | -4.79 | 2.79E-62 | 5.14E-58 |
| Pmel | 10 | -5.4 | 8.33E-44 | 7.69E-40 |
| Glb1l3 | 9 | -6.64 | 6.09E-36 | 2.81E-32 |
| Hdc | 2 | -6.34 | 7.56E-35 | 2.79E-31 |
| Taf4b | 18 | -2.43 | 5.13E-29 | 1.05E-25 |
| Cdcp3 | 7 | -2.2 | 5.51E-28 | 9.24E-25 |
| Ttll11 | 2 | -2.37 | 1.17E-27 | 1.79E-24 |
| Ccdc187 | 2 | 3.57 | 1.17E-26 | 1.54E-23 |
| Dct | 14 | -3.29 | 2.33E-26 | 2.87E-23 |
| Gpnmb | 6 | -2.67 | 2.30E-24 | 2.50E-21 |
| Col6a1 | 10 | 2.08 | 3.19E-23 | 2.81E-20 |
| Cryba1 | 11 | 51.3 | 1.02E-21 | 7.84E-19 |
| Nxph4 | 10 | 2.23 | 1.78E-19 | 1.17E-16 |
| Col7a1 | 9 | 2.05 | 5.42E-19 | 3.23E-16 |
| Col5a3 | 9 | 3.07 | 1.39E-18 | 7.56E-16 |
| Olfr769 | 10 | 15.2 | 7.89E-18 | 3.16E-15 |
| Col4a4 | 1 | -2.14 | 1.91E-17 | 7.05E-15 |
| 5730507C01Rik | 12 | -2.5 | 6.51E-17 | 2.24E-14 |
| Crygs | 16 | 51.3 | 1.34E-16 | 4.33E-14 |
| Prtg | 9 | 2.74 | 2.85E-16 | 8.77E-14 |
| Eno1b | 18 | -6.45 | 3.18E-16 | 9.47E-14 |
| Tyr | 7 | -4.61 | 1.21E-15 | 3.18E-13 |
| Kif19a | 11 | 2.82 | 2.23E-15 | 5.55E-13 |
| Gm20721 | 2 | -2.76 | 9.90E-15 | 2.17E-12 |
| Fn1 | 1 | 2.08 | 2.66E-14 | 5.50E-12 |
| Tyrp1 | 4 | -5.31 | 7.18E-14 | 1.33E-11 |
| Cryba4 | 5 | 20.8 | 9.98E-14 | 1.72E-11 |
| Tnnt2 | 1 | 4.71 | 1.28E-13 | 2.19E-11 |
| Gm14698 | X | -2.32 | 1.52E-13 | 2.55E-11 |
| Crybb1 | 5 | 19 | 2.46E-13 | 3.81E-11 |
| Adamts19 | 18 | 7.5 | 5.20E-13 | 7.73E-11 |
| Crybb3 | 5 | 10.3 | 8.29E-13 | 1.19E-10 |
| Cryaa | 17 | 43.8 | 8.80E-13 | 1.25E-10 |
| Ybx2 | 11 | 2.94 | 8.94E-13 | 1.26E-10 |
| Id3 | 4 | -2.69 | 1.36E-12 | 1.82E-10 |
| Atf5 | 7 | 2.38 | 1.47E-12 | 1.95E-10 |
| Tmem26 | 10 | 2.86 | 2.19E-12 | 2.80E-10 |
| Crybb2 | 5 | 40.7 | 2.86E-12 | 3.53E-10 |

| | | | | |
|---|---|---|---|---|
| Shisal2b | 13 | -7.63 | 4.71E-12 | 5.50E-10 |
| Prss56 | 1 | 20.1 | 8.53E-12 | 9.37E-10 |
| Crygb | 1 | 39.8 | 1.03E-11 | 1.10E-09 |
| Cryba2 | 1 | 13.2 | 1.17E-11 | 1.24E-09 |
| Crygd | 1 | 51.8 | 1.29E-11 | 1.32E-09 |
| Mlana | 19 | -7.9 | 3.19E-11 | 2.95E-09 |
| Lars2 | 9 | 5.61 | 4.74E-11 | 4.14E-09 |
| Obscn | 11 | 2.24 | 1.06E-10 | 8.65E-09 |
| Mcam | 9 | 2.08 | 1.34E-10 | 1.07E-08 |
| Alpk2 | 18 | -2.35 | 3.45E-10 | 2.48E-08 |
| Pdzd7 | 19 | 2.37 | 5.57E-10 | 3.80E-08 |
| 5530401A14Rik | 11 | 2.2 | 1.07E-09 | 6.82E-08 |
| Nes | 3 | 4.69 | 1.32E-09 | 8.24E-08 |
| Crygc | 1 | 31.4 | 1.91E-09 | 1.15E-07 |
| Gm21897 | 16 | 4.67 | 1.89E-09 | 1.15E-07 |
| Mip | 10 | 10.5 | 2.08E-09 | 1.24E-07 |
| Pagr1a | 7 | 14.7 | 3.94E-09 | 2.13E-07 |
| Heph | X | -2.11 | 4.45E-09 | 2.37E-07 |
| Hba-a2 | 11 | 2.77 | 4.77E-09 | 2.52E-07 |
| Kif4 | X | -2.2 | 6.67E-09 | 3.41E-07 |
| Lad1 | 1 | 4.31 | 8.31E-09 | 4.17E-07 |
| Mfrp | 9 | -2.28 | 8.44E-09 | 4.21E-07 |
| Nkd1 | 8 | 2.63 | 1.06E-08 | 5.15E-07 |
| Gsta3 | 1 | -3.16 | 1.20E-08 | 5.73E-07 |
| Cryge | 1 | 289.8 | 1.85E-08 | 8.49E-07 |
| Gm21962 | 3 | 4.29 | 1.91E-08 | 8.72E-07 |
| Crygf | 1 | 73.2 | 2.25E-08 | 1.00E-06 |
| Dleu7 | 14 | -2.69 | 2.24E-08 | 1.00E-06 |
| Adamts4 | 1 | 2.58 | 2.28E-08 | 1.01E-06 |
| H2ac12 | 13 | -10.8 | 2.78E-08 | 1.21E-06 |
| Suclg2 | 6 | -2.32 | 2.85E-08 | 1.24E-06 |
| Spc25 | 2 | -2.01 | 3.42E-08 | 1.45E-06 |
| Mup4 | 4 | -9.05 | 3.42E-08 | 1.45E-06 |
| Islr | 9 | -3.09 | 4.29E-08 | 1.78E-06 |
| Papss2 | 19 | -2.34 | 6.98E-08 | 2.73E-06 |
| Gm1604b | 17 | 5.7 | 8.38E-08 | 3.17E-06 |
| Nupr1 | 7 | 3.77 | 1.30E-07 | 4.69E-06 |
| Obp1b | X | -5.76 | 1.53E-07 | 5.36E-06 |
| Gm3839 | 14 | -3.34 | 1.81E-07 | 6.24E-06 |
| Knl1 | 2 | -2.19 | 1.93E-07 | 6.57E-06 |

| | | | | |
|---|---|---|---|---|
| Loxl4 | 19 | 2.49 | 2.18E-07 | 7.34E-06 |
| Col3a1 | 1 | 2.98 | 2.51E-07 | 8.36E-06 |
| Zic1 | 9 | -2.46 | 3.09E-07 | 9.93E-06 |
| Myrf | 19 | 2.04 | 4.23E-07 | 1.30E-05 |
| Bpifa1 | 2 | -13.9 | 4.34E-07 | 1.32E-05 |
| Lgsn | 1 | 12 | 4.40E-07 | 1.34E-05 |
| Efemp1 | 11 | -2.11 | 4.48E-07 | 1.36E-05 |
| Slfn4 | 11 | 22.8 | 4.66E-07 | 1.41E-05 |
| Col19a1 | 1 | -2.38 | 4.99E-07 | 1.49E-05 |
| Adgrg6 | 10 | 2.54 | 5.29E-07 | 1.57E-05 |
| Plb1 | 5 | 2.52 | 5.44E-07 | 1.61E-05 |
| Fam167a | 14 | 2.48 | 6.47E-07 | 1.89E-05 |
| Serpinb9 | 13 | -2 | 7.56E-07 | 2.16E-05 |
| Nr4a3 | 4 | 2.39 | 8.86E-07 | 2.48E-05 |
| Nid1 | 13 | 2.19 | 1.06E-06 | 2.86E-05 |
| Slc22a29 | 19 | -2.28 | 1.13E-06 | 3.01E-05 |
| Cdh19 | 1 | -3.41 | 1.14E-06 | 3.05E-05 |
| Amd2 | 10 | -2.71 | 1.15E-06 | 3.07E-05 |
| Gm14744 | X | -6.86 | 1.19E-06 | 3.14E-05 |
| Aass | 6 | -3.08 | 1.20E-06 | 3.16E-05 |
| Flnc | 6 | 2.2 | 1.33E-06 | 3.44E-05 |
| A3galt2 | 4 | 2.14 | 1.38E-06 | 3.56E-05 |
| Tmem140 | 6 | 3.92 | 1.43E-06 | 3.65E-05 |
| Ppp1r14a | 7 | 2.52 | 1.61E-06 | 4.05E-05 |
| Maob | X | -2.49 | 1.82E-06 | 4.48E-05 |
| Gm8797 | 3 | -3.14 | 2.02E-06 | 4.89E-05 |
| Tagln2 | 1 | 2.43 | 2.03E-06 | 4.90E-05 |
| Synpo | 18 | 2.06 | 2.67E-06 | 6.15E-05 |
| Hsd17b2 | 8 | 3.16 | 2.68E-06 | 6.16E-05 |
| Gm10184 | 17 | -9.59 | 2.68E-06 | 6.16E-05 |
| Ip6k3 | 17 | -2.24 | 2.95E-06 | 6.70E-05 |
| Zpbp | 11 | -2.04 | 3.31E-06 | 7.38E-05 |
| Lif | 11 | 4.96 | 3.98E-06 | 8.66E-05 |
| Il6ra | 3 | 2.46 | 4.28E-06 | 9.16E-05 |
| Sh2d1a | X | -2.2 | 5.04E-06 | 1.05E-04 |
| Pycr1 | 11 | 2.19 | 5.70E-06 | 1.17E-04 |
| Cped1 | 6 | -2.31 | 7.15E-06 | 1.42E-04 |
| Misp | 10 | 2.41 | 7.29E-06 | 1.44E-04 |
| Niban1 | 1 | -2.5 | 8.28E-06 | 1.60E-04 |
| Tbx22 | X | -5 | 8.34E-06 | 1.61E-04 |

| | | | | |
|---|---|---|---|---|
| Akr1c14 | 13 | -5.06 | 8.91E-06 | 1.70E-04 |
| Epha2 | 4 | 2.52 | 9.74E-06 | 1.81E-04 |
| Kcne1l | X | -2.68 | 1.01E-05 | 1.88E-04 |
| Spsb1 | 4 | 2.51 | 1.02E-05 | 1.89E-04 |
| Cryga | 1 | 25.4 | 1.04E-05 | 1.92E-04 |
| Gm14434 | 2 | -12.2 | 1.05E-05 | 1.93E-04 |
| Unc13d | 11 | 2.14 | 1.05E-05 | 1.93E-04 |
| Klhdc7b | 15 | 3.25 | 1.05E-05 | 1.93E-04 |
| Fosl1 | 19 | 6.18 | 1.35E-05 | 2.37E-04 |
| Pld5 | 1 | -2.01 | 1.49E-05 | 2.57E-04 |
| Ap1g2 | 14 | 2.09 | 1.52E-05 | 2.61E-04 |
| Siglec1 | 2 | 5.11 | 1.53E-05 | 2.62E-04 |
| Gm49388 | 7 | 831.3 | 1.66E-05 | 2.81E-04 |
| Sh3rf2 | 18 | -6.49 | 1.71E-05 | 2.88E-04 |
| Slc26a10 | 10 | 3.18 | 1.76E-05 | 2.95E-04 |
| Tnfsf10 | 3 | -3.1 | 1.79E-05 | 2.99E-04 |
| Bace2 | 16 | -2.28 | 1.82E-05 | 3.04E-04 |
| Fbln5 | 12 | -2.61 | 1.86E-05 | 3.10E-04 |
| Ror2 | 13 | 2.33 | 1.97E-05 | 3.25E-04 |
| Obp1a | X | -4.12 | 2.28E-05 | 3.65E-04 |
| Litaf | 16 | 2.21 | 2.51E-05 | 3.96E-04 |
| Pmp2 | 3 | -2.94 | 2.56E-05 | 4.03E-04 |
| Cspg4b | 13 | -2.17 | 2.83E-05 | 4.36E-04 |
| Tph1 | 7 | 2.97 | 2.87E-05 | 4.41E-04 |
| Ackr3 | 1 | -2.14 | 2.97E-05 | 4.53E-04 |
| L3mbtl1 | 2 | 2.14 | 3.03E-05 | 4.61E-04 |
| Col24a1 | 3 | 2.16 | 3.17E-05 | 4.77E-04 |
| Bfsp1 | 2 | 4.8 | 3.20E-05 | 4.81E-04 |
| Agmo | 12 | -2.84 | 3.68E-05 | 5.40E-04 |
| Gpx3 | 11 | -8.09 | 3.71E-05 | 5.44E-04 |
| Tlr2 | 3 | 2.93 | 4.03E-05 | 5.85E-04 |
| Acoxl | 2 | -2.15 | 4.29E-05 | 6.17E-04 |
| Gsc2 | 16 | -2.73 | 4.38E-05 | 6.26E-04 |
| Pvalb | 15 | 2.07 | 4.76E-05 | 6.71E-04 |
| Ect2 | 3 | -2.28 | 5.31E-05 | 7.38E-04 |
| Hmga2 | 10 | 3.08 | 5.38E-05 | 7.46E-04 |
| Rassf10 | 7 | -2.51 | 6.12E-05 | 8.33E-04 |
| Egr2 | 10 | 2.73 | 6.35E-05 | 8.58E-04 |
| Aoc3 | 11 | 2.01 | 6.83E-05 | 9.17E-04 |
| Col6a6 | 9 | 2.05 | 7.08E-05 | 9.42E-04 |

| | | | | |
|---|---|---|---|---|
| Aldh3a1 | 11 | -5.85 | 7.21E-05 | 9.55E-04 |
| Npsr1 | 9 | -2.79 | 7.27E-05 | 9.60E-04 |
| Penk | 4 | -5.56 | 7.30E-05 | 9.63E-04 |
| Otx1 | 11 | -4.37 | 7.67E-05 | 1.00E-03 |
| Olfm4 | 14 | -2.08 | 7.68E-05 | 1.01E-03 |
| Cebpd | 16 | 3.19 | 7.75E-05 | 1.01E-03 |
| Mgst1 | 6 | -2.15 | 8.77E-05 | 1.12E-03 |
| Ugt1a6a | 1 | -2.74 | 8.90E-05 | 1.13E-03 |
| Glycam1 | 15 | -7.01 | 8.88E-05 | 1.13E-03 |
| Kcp | 6 | 2.78 | 9.19E-05 | 1.16E-03 |
| Ccl5 | 11 | 5.26 | 9.29E-05 | 1.17E-03 |
| Srp54b | 12 | -8.44 | 9.32E-05 | 1.17E-03 |
| Creb3l1 | 2 | 2.07 | 9.85E-05 | 1.22E-03 |
| Slc26a4 | 12 | -11.1 | 9.87E-05 | 1.22E-03 |
| Rtp3 | 9 | 2.13 | 9.98E-05 | 1.23E-03 |
| Gm45861 | 8 | 2.42 | 1.02E-04 | 1.26E-03 |
| Maff | 15 | 2.49 | 1.02E-04 | 1.26E-03 |
| Tph2 | 10 | -2.2 | 1.10E-04 | 1.34E-03 |
| 5430402E10Rik | X | -4.18 | 1.10E-04 | 1.34E-03 |
| Cebpa | 7 | 2.1 | 1.11E-04 | 1.35E-03 |
| Exoc3l4 | 12 | -3.8 | 1.19E-04 | 1.42E-03 |
| Foxi3 | 6 | -2.83 | 1.29E-04 | 1.53E-03 |
| Mlph | 1 | -2.85 | 1.38E-04 | 1.61E-03 |
| Slc15a3 | 19 | 2.44 | 1.39E-04 | 1.62E-03 |
| Pgf | 12 | 2.77 | 1.49E-04 | 1.70E-03 |
| Fxyd5 | 7 | 2.14 | 1.54E-04 | 1.74E-03 |
| Sostdc1 | 12 | -2.28 | 1.55E-04 | 1.75E-03 |
| Ccr2 | 9 | 5.03 | 1.62E-04 | 1.82E-03 |
| Cdsn | 17 | 2.55 | 1.64E-04 | 1.83E-03 |
| Eif4ebp1 | 8 | 2.16 | 1.68E-04 | 1.87E-03 |
| Obp2a | 2 | -5.54 | 1.72E-04 | 1.91E-03 |
| Matn3 | 12 | -11.7 | 1.76E-04 | 1.94E-03 |
| Tedc1 | 12 | -2.37 | 1.83E-04 | 1.99E-03 |
| Vcan | 13 | -3.29 | 1.93E-04 | 2.09E-03 |
| Ptgfr | 3 | 3.87 | 1.99E-04 | 2.13E-03 |
| Stac3 | 10 | 2.22 | 2.20E-04 | 2.31E-03 |
| Slc39a12 | 2 | -2.03 | 2.21E-04 | 2.32E-03 |
| Tmem252 | 19 | 2.21 | 2.26E-04 | 2.36E-03 |
| Prrx1 | 1 | -2.23 | 2.39E-04 | 2.47E-03 |
| Eps8l1 | 7 | 2.3 | 2.43E-04 | 2.50E-03 |

| | | | | | |
|---|---|---|---|---|---|
| Hrc | | 7 | 2.46 | 2.54E-04 | 2.60E-03 |
| Draxin | | 4 | 2.17 | 2.56E-04 | 2.62E-03 |
| Zfp185 | X | | -3.04 | 2.59E-04 | 2.64E-03 |
| Plscr2 | | 9 | -2.62 | 2.62E-04 | 2.67E-03 |
| Des | | 1 | 2.31 | 2.86E-04 | 2.88E-03 |
| Slc16a12 | | 19 | -3.94 | 2.98E-04 | 2.99E-03 |
| Arhgef16 | | 4 | -2.12 | 3.00E-04 | 3.00E-03 |
| Olfr455 | | 6 | 2.53 | 3.06E-04 | 3.05E-03 |
| Gadd45b | | 10 | 2.22 | 3.10E-04 | 3.09E-03 |
| Prrg1 | X | | -2.15 | 3.13E-04 | 3.11E-03 |
| Piwil4 | | 9 | -3.7 | 3.20E-04 | 3.16E-03 |
| Folr1 | | 7 | -2.45 | 3.36E-04 | 3.30E-03 |
| Mme | | 3 | -2.69 | 3.48E-04 | 3.40E-03 |
| Gm10282 | | 8 | -2.35 | 3.51E-04 | 3.42E-03 |
| Cxcl2 | | 5 | 6.13 | 3.56E-04 | 3.45E-03 |
| Myc | | 15 | 2.72 | 3.72E-04 | 3.57E-03 |
| Gm21985 | | 2 | 2.52 | 3.76E-04 | 3.60E-03 |
| Ogn | | 13 | -4.32 | 3.81E-04 | 3.65E-03 |
| H2-Ab1 | | 17 | 3.26 | 3.89E-04 | 3.70E-03 |
| Gja1 | | 10 | -3.31 | 4.01E-04 | 3.80E-03 |
| Mndal | | 1 | 3.01 | 4.03E-04 | 3.83E-03 |
| Veph1 | | 3 | -3.32 | 4.07E-04 | 3.85E-03 |
| Klhdc7a | | 4 | -4.04 | 4.08E-04 | 3.87E-03 |
| Six1 | | 12 | -2.21 | 4.18E-04 | 3.94E-03 |
| Gimap6 | | 6 | 2.89 | 4.30E-04 | 4.03E-03 |
| Fam177a2 | | 12 | 14.6 | 4.59E-04 | 4.25E-03 |
| Dlx3 | | 11 | 3.13 | 4.72E-04 | 4.35E-03 |
| Thbd | | 2 | -2.11 | 4.73E-04 | 4.36E-03 |
| Robo4 | | 9 | 2.12 | 4.74E-04 | 4.37E-03 |
| Zfp979 | | 4 | -2.01 | 5.03E-04 | 4.59E-03 |
| Sod3 | | 5 | -3.85 | 5.09E-04 | 4.63E-03 |
| Fbxo47 | | 11 | -2.17 | 5.34E-04 | 4.83E-03 |
| 4930523C07Rik | | 1 | -2.07 | 5.37E-04 | 4.85E-03 |
| Nkx6-2 | | 7 | 3.12 | 5.39E-04 | 4.86E-03 |
| Slfn5 | | 11 | 2.11 | 5.67E-04 | 5.07E-03 |
| Olfr552 | | 7 | 8.82 | 5.76E-04 | 5.13E-03 |
| Cd74 | | 18 | 3.23 | 6.49E-04 | 5.64E-03 |
| Obp2b | | 2 | -10.7 | 6.69E-04 | 5.81E-03 |
| Kcng1 | | 2 | 3.76 | 6.87E-04 | 5.93E-03 |
| Ccl2 | | 11 | 5.01 | 6.97E-04 | 6.01E-03 |

| | | | | |
|---|---|---|---|---|
| Tbx6 | 7 | 2.16 | 7.00E-04 | 6.03E-03 |
| Lum | 10 | -4.54 | 7.09E-04 | 6.08E-03 |
| Mmp23 | 4 | 3.63 | 7.24E-04 | 6.19E-03 |
| Vxn | 1 | -2.2 | 7.61E-04 | 6.44E-03 |
| Rps27rt | 9 | 2.7 | 7.65E-04 | 6.47E-03 |
| Slc22a12 | 19 | 5.87 | 7.70E-04 | 6.49E-03 |
| Ch25h | 19 | -3.74 | 8.01E-04 | 6.69E-03 |
| Chek2 | 5 | 2.14 | 8.32E-04 | 6.90E-03 |
| Nuf2 | 1 | -2.32 | 8.53E-04 | 7.02E-03 |
| Sp100 | 1 | 2.26 | 8.78E-04 | 7.19E-03 |
| Myh11 | 16 | -2.07 | 8.84E-04 | 7.22E-03 |
| Depp1 | 6 | 2.24 | 8.91E-04 | 7.26E-03 |
| Itgal | 7 | 2.83 | 8.96E-04 | 7.29E-03 |
| Gvin2 | 7 | 2.14 | 9.04E-04 | 7.34E-03 |
| Dcn | 10 | -2.9 | 9.08E-04 | 7.36E-03 |
| Matn2 | 15 | -2.04 | 9.19E-04 | 7.43E-03 |
| Slc25a21 | 12 | -3.66 | 9.27E-04 | 7.48E-03 |
| Anxa8 | 14 | -6.85 | 9.37E-04 | 7.53E-03 |
| Gm10358 | 8 | -2.18 | 9.89E-04 | 7.87E-03 |
| Prr18 | 17 | 2.19 | 9.88E-04 | 7.87E-03 |
| AU021092 | 16 | 2.35 | 9.92E-04 | 7.89E-03 |
| Ltf | 9 | -6.34 | 9.94E-04 | 7.90E-03 |
| 2410004P03Rik | 12 | 3.34 | 1.02E-03 | 8.05E-03 |
| F10 | 8 | 4.28 | 1.05E-03 | 8.21E-03 |
| Sox30 | 11 | 2.03 | 1.08E-03 | 8.38E-03 |
| Ucn2 | 9 | 3.12 | 1.10E-03 | 8.51E-03 |
| Gm27179 | 14 | 182.5 | 1.11E-03 | 8.56E-03 |
| Plbd1 | 6 | 4.01 | 1.14E-03 | 8.74E-03 |
| Lefty1 | 1 | -7.64 | 1.15E-03 | 8.79E-03 |
| Ccdc188 | 16 | 2.63 | 1.16E-03 | 8.84E-03 |
| Arhgap25 | 6 | 2.62 | 1.24E-03 | 9.34E-03 |
| Gm6588 | 5 | 2.23 | 1.24E-03 | 9.35E-03 |
| Krt6b | 15 | -4.46 | 1.24E-03 | 9.37E-03 |
| H2-Q7 | 17 | 2.08 | 1.26E-03 | 9.46E-03 |
| Ifi203 | 1 | 2.36 | 1.27E-03 | 9.51E-03 |
| Olfr1031 | 2 | 19.5 | 1.27E-03 | 9.52E-03 |
| Scn4b | 9 | 2.35 | 1.28E-03 | 9.58E-03 |
| Creb3l3 | 10 | 2.43 | 1.31E-03 | 9.74E-03 |
| Myo1f | 17 | 2.21 | 1.32E-03 | 9.79E-03 |
| Gm12185 | 11 | -5.86 | 1.33E-03 | 9.83E-03 |

| | | | | | |
|---|---|---|---|---|---|
| Krt15 | | 11 | -5.68 | 1.33E-03 | 9.83E-03 |
| Ripply3 | | 16 | -4.47 | 1.40E-03 | 0.010259936 |
| Cox8b | | 7 | -2.65 | 1.42E-03 | 0.010384541 |
| Lmntd2 | | 7 | 2.36 | 1.42E-03 | 0.010384541 |
| Gm21833 | | 16 | -5.57 | 1.44E-03 | 0.010487539 |
| Cndp1 | | 18 | -6.47 | 1.45E-03 | 0.010588848 |
| Fbln7 | | 2 | -2.81 | 1.46E-03 | 0.010634453 |
| Ecrg4 | | 1 | -3.48 | 1.48E-03 | 0.010751299 |
| Ccl21d | | 4 | -25.7 | 1.51E-03 | 0.010880632 |
| Tfcp2l1 | | 1 | -2.11 | 1.55E-03 | 0.011130842 |
| Slc7a10 | | 7 | 2.58 | 1.57E-03 | 0.011251919 |
| Aldh1a3 | | 7 | 2.25 | 1.59E-03 | 0.011323069 |
| Ndufa4l2 | | 10 | 2.15 | 1.59E-03 | 0.011354527 |
| F5 | | 1 | -4.09 | 1.60E-03 | 0.011417051 |
| Slc47a1 | | 11 | 2.19 | 1.61E-03 | 0.011457215 |
| Gm10591 | | 4 | -26.5 | 1.76E-03 | 0.012263464 |
| Aplnr | | 2 | 3.49 | 1.78E-03 | 0.012378487 |
| Gja8 | | 3 | 3.21 | 1.82E-03 | 0.012602233 |
| A830005F24Rik | | 13 | 2.74 | 1.83E-03 | 0.012663622 |
| Baz1a | | 12 | 2.08 | 1.86E-03 | 0.012826523 |
| Gstm2 | | 3 | -3.31 | 1.88E-03 | 0.012898301 |
| Ccl3 | | 11 | 3.63 | 1.91E-03 | 0.013054641 |
| Fat2 | | 11 | -4.39 | 1.96E-03 | 0.013374034 |
| Atp1a2 | | 1 | -2.74 | 1.96E-03 | 0.013400167 |
| Sytl1 | | 4 | 2.65 | 1.97E-03 | 0.013452875 |
| Musk | | 4 | -3.97 | 1.98E-03 | 0.013514063 |
| Plac8 | | 5 | 5.54 | 1.99E-03 | 0.013514507 |
| Ifi207 | | 1 | 3.32 | 1.99E-03 | 0.013534307 |
| Paqr6 | | 3 | -2.01 | 2.01E-03 | 0.013610124 |
| Fcgr1 | | 3 | 2.4 | 2.04E-03 | 0.013835565 |
| Krt5 | | 15 | -3.74 | 2.07E-03 | 0.013974886 |
| Slc22a28 | | 19 | -3.13 | 2.13E-03 | 0.014252946 |
| Gm10719 | | 9 | 2.37 | 2.21E-03 | 0.014656609 |
| Crhbp | | 13 | -2.61 | 2.24E-03 | 0.014817869 |
| Rnf43 | | 11 | -2.02 | 2.24E-03 | 0.014830862 |
| Emp1 | | 6 | -2.09 | 2.54E-03 | 0.016370892 |
| Krt6a | | 15 | -3.33 | 2.60E-03 | 0.016695913 |
| Rapsn | | 2 | -2.32 | 2.85E-03 | 0.018014097 |
| Cybb | X | | 2.63 | 2.93E-03 | 0.018378047 |
| Gm44505 | | 19 | -7.18 | 2.97E-03 | 0.018575363 |

| | | | | |
|---|---|---|---|---|
| Lax1 | 1 | 3.83 | 3.02E-03 | 0.018825354 |
| Gm20075 | 13 | -2.05 | 3.04E-03 | 0.018911812 |
| Grifin | 5 | 5.04 | 3.09E-03 | 0.019133859 |
| Lgals12 | 19 | -2.21 | 3.09E-03 | 0.019156737 |
| Cgas | 9 | 2.59 | 3.10E-03 | 0.019197018 |
| Kcnmb1 | 11 | 2.15 | 3.15E-03 | 0.019428642 |
| Jak3 | 8 | 2.07 | 3.20E-03 | 0.019648797 |
| 4933405O20Rik | 7 | 3 | 3.30E-03 | 0.020129527 |
| Pabpc4l | 3 | -2.53 | 3.34E-03 | 0.020286543 |
| Ehf | 2 | -2.02 | 3.40E-03 | 0.020633183 |
| 4930590J08Rik | 6 | 2.01 | 3.47E-03 | 0.02097648 |
| Ifi211 | 1 | 3.47 | 3.61E-03 | 0.021604393 |
| Nkd2 | 13 | -2.03 | 3.68E-03 | 0.02191808 |
| Galnt6 | 15 | 2.78 | 3.74E-03 | 0.02223616 |
| Mapk15 | 15 | 2.05 | 3.76E-03 | 0.022317411 |
| Cxcl10 | 5 | 4 | 3.77E-03 | 0.022330273 |
| Fam71d | 12 | 2.16 | 3.91E-03 | 0.02303071 |
| Gm45337 | 7 | 2.51 | 3.92E-03 | 0.023079934 |
| C3 | 17 | 2.5 | 3.97E-03 | 0.02327811 |
| H2-Aa | 17 | 3.07 | 4.00E-03 | 0.023405047 |
| Arid3c | 4 | 2.86 | 4.05E-03 | 0.023608255 |
| Yy2 | X | 2.3 | 4.05E-03 | 0.023608255 |
| Stap2 | 17 | 2.45 | 4.06E-03 | 0.02365736 |
| Aoah | 13 | 3.43 | 4.10E-03 | 0.02387159 |
| Csf2rb2 | 15 | 2.03 | 4.11E-03 | 0.023922841 |
| Serpine3 | 14 | 2.75 | 4.12E-03 | 0.02394674 |
| Cstdc5 | 16 | -5.21 | 4.43E-03 | 0.02535614 |
| Olfml2a | 2 | -2.4 | 4.54E-03 | 0.025857452 |
| Clec18a | 8 | -2.08 | 4.60E-03 | 0.026140885 |
| Msx1 | 5 | -4.28 | 4.60E-03 | 0.026152061 |
| Vmn2r30 | 7 | -2.76 | 4.72E-03 | 0.026679163 |
| Gm37389 | 3 | -7.58 | 4.90E-03 | 0.027329066 |
| Gm28635 | 2 | -179.3 | 4.95E-03 | 0.027584198 |
| Mrgprf | 7 | -2.74 | 4.96E-03 | 0.027610338 |
| Foxd2 | 4 | -3.07 | 4.99E-03 | 0.027708859 |
| Colec12 | 18 | -2.55 | 5.02E-03 | 0.027826754 |
| Tmprss5 | 9 | -2.02 | 5.15E-03 | 0.028450432 |
| H2-Eb1 | 17 | 3.11 | 5.16E-03 | 0.028471465 |
| Rassf9 | 10 | -2.12 | 5.19E-03 | 0.028585109 |
| Apobr | 7 | 2.42 | 5.31E-03 | 0.029121256 |

| | | | | |
|---|---|---|---|---|
| Angptl4 | 17 | 2.15 | 5.33E-03 | 0.029245326 |
| Zbp1 | 2 | 2.82 | 5.39E-03 | 0.029444098 |
| Il2rg | X | 2.34 | 5.47E-03 | 0.029823208 |
| Bmp4 | 14 | -2.09 | 5.49E-03 | 0.029904974 |
| Oas3 | 5 | 3.3 | 5.50E-03 | 0.029944086 |
| P4ha3 | 7 | 2.02 | 5.60E-03 | 0.030390488 |
| Ccdc68 | 18 | -2.99 | 5.67E-03 | 0.030676184 |
| Muc4 | 16 | -2.59 | 5.73E-03 | 0.030933091 |
| Vmn2r118 | 17 | 2.14 | 5.81E-03 | 0.03126935 |
| Zfp972 | 2 | 2.18 | 5.94E-03 | 0.031799214 |
| Cilp | 9 | 2.35 | 6.06E-03 | 0.032321045 |
| Mdfic2 | 6 | -2.1 | 6.16E-03 | 0.032731027 |
| H2-Q5 | 17 | 2.96 | 6.53E-03 | 0.034189365 |
| Aldh1a2 | 9 | -3.28 | 6.63E-03 | 0.034634472 |
| Ermap | 4 | 2.35 | 6.71E-03 | 0.034953888 |
| Oas1a | 5 | 2.51 | 6.73E-03 | 0.035010176 |
| Bcl3 | 7 | 2.31 | 6.75E-03 | 0.035113414 |
| Cfb | 17 | 2.88 | 6.92E-03 | 0.035809119 |
| Map3k6 | 4 | 2.06 | 7.15E-03 | 0.036674029 |
| Ltbp2 | 12 | -4.14 | 7.27E-03 | 0.037190205 |
| Hrh1 | 6 | 2.59 | 7.35E-03 | 0.037489389 |
| Fam205a3 | 4 | -2.43 | 7.74E-03 | 0.039100873 |
| Klhl14 | 18 | -2.58 | 7.84E-03 | 0.03945617 |
| Krt13 | 11 | -4.03 | 8.01E-03 | 0.040003214 |
| Gm12184 | 11 | -2.66 | 8.04E-03 | 0.040141892 |
| Syk | 13 | 2.05 | 8.23E-03 | 0.040904202 |
| Mfap4 | 11 | -4.41 | 8.31E-03 | 0.041204495 |
| Dsg1a | 18 | -4.62 | 8.36E-03 | 0.041351419 |
| Vmn2r7 | 3 | -3.33 | 8.52E-03 | 0.041914586 |
| Adora2a | 10 | 2.25 | 8.53E-03 | 0.041955881 |
| Vmn2r54 | 7 | 3.12 | 8.59E-03 | 0.042151187 |
| Arl13a | X | -2.07 | 8.60E-03 | 0.042227291 |
| Muc3a | 5 | 2.06 | 8.69E-03 | 0.042567826 |
| Hmga1b | 11 | -2.19 | 8.97E-03 | 0.043677442 |
| Kif19b | 5 | 2.71 | 9.00E-03 | 0.043762664 |
| Ccl21b | 4 | -7.98 | 9.00E-03 | 0.043764624 |
| Abcc6 | 7 | 2.63 | 9.03E-03 | 0.043869876 |
| Mog | 17 | 2.75 | 9.13E-03 | 0.044239043 |
| Defb11 | 8 | -5.93 | 9.60E-03 | 0.046107434 |
| Slc13a4 | 6 | -2.91 | 9.64E-03 | 0.046207601 |

| | | | | |
|---|---|---|---|---|
| Gm715 | X | -2.96 | 9.68E-03 | 0.046325594 |
| Sned1 | 1 | -2.09 | 0.010015811 | 0.047662959 |
| Pik3r6 | 11 | 2.46 | 0.010193454 | 0.048296494 |
| 2810459M11Rik | 1 | -2.3 | 0.01030234 | 0.048712297 |
| Krt12 | 11 | -2.85 | 0.010464344 | 0.04926361 |
| Rfx4 | 10 | -2.26 | 0.010486301 | 0.049341791 |

**Supplementary Table 4: Fluvastatin + MBV vs CTR**

| Name | Chromosome | Fold change | P-value | FDR p-value |
|---|---|---|---|---|
| Cfb | 17 | 8.44 | 1.57E-71 | 2.73E-67 |
| Cd74 | 18 | 7.81 | 1.14E-70 | 9.97E-67 |
| Ccr2 | 9 | 14.92 | 2.64E-59 | 1.54E-55 |
| H2-Aa | 17 | 8.9 | 4.54E-54 | 1.98E-50 |
| C3 | 17 | 7.33 | 4.81E-52 | 1.68E-48 |
| H2-Eb1 | 17 | 7.7 | 2.24E-46 | 6.51E-43 |
| H2-Ab1 | 17 | 6.88 | 2.25E-45 | 5.60E-42 |
| Nr4a3 | 4 | 4.72 | 1.13E-31 | 2.46E-28 |
| Cybb | X | 4.63 | 4.65E-26 | 7.36E-23 |
| Lyz2 | 10 | 4.85 | 7.69E-25 | 1.12E-21 |
| C1ra | 6 | 2.95 | 3.07E-24 | 3.82E-21 |
| Pagr1a | 7 | 20.86 | 2.11E-22 | 2.45E-19 |
| Ctss | 3 | 2.1 | 4.85E-22 | 5.28E-19 |
| Mpeg1 | 19 | 2.15 | 2.36E-19 | 2.28E-16 |
| Chil1 | 1 | 3.07 | 6.27E-19 | 5.46E-16 |
| Plbd1 | 6 | 6.9 | 2.77E-16 | 2.10E-13 |
| Tgfbi | 13 | 4.03 | 4.08E-16 | 2.96E-13 |
| Itgax | 7 | 5.47 | 5.33E-15 | 3.58E-12 |
| Ptprc | 1 | 2.87 | 8.15E-15 | 5.26E-12 |
| Cables1 | 18 | 2.07 | 2.15E-13 | 1.25E-10 |
| Ccl6 | 11 | 4.2 | 7.89E-13 | 4.30E-10 |
| Saa3 | 7 | 6.44 | 1.87E-12 | 9.56E-10 |
| C1s1 | 6 | 2.96 | 3.49E-12 | 1.74E-09 |
| Pirb | 7 | 3.69 | 1.20E-11 | 5.36E-09 |
| Slfn5 | 11 | 2.42 | 3.27E-11 | 1.36E-08 |
| Slfn2 | 11 | 3.82 | 7.21E-11 | 2.92E-08 |
| Itgam | 7 | 2 | 1.66E-10 | 6.15E-08 |
| Adgre1 | 17 | 2.77 | 2.13E-10 | 7.58E-08 |
| Lcn2 | 2 | 4.2 | 4.39E-10 | 1.53E-07 |
| Siglec1 | 2 | 3.99 | 5.74E-10 | 1.92E-07 |
| Clec7a | 6 | 2.95 | 8.58E-10 | 2.72E-07 |
| Clec12a | 6 | 5.94 | 1.87E-09 | 5.52E-07 |
| Slfn4 | 11 | 9.95 | 2.73E-09 | 7.94E-07 |
| Adgrl4 | 3 | 2.03 | 3.77E-09 | 1.03E-06 |
| Gvin2 | 7 | 2.42 | 4.45E-09 | 1.19E-06 |
| C3ar1 | 6 | 2.46 | 4.96E-09 | 1.29E-06 |
| Syk | 13 | 3.22 | 5.95E-09 | 1.52E-06 |
| Gda | 19 | 3.12 | 6.41E-09 | 1.60E-06 |

| | | | | |
|---|---|---|---|---|
| Myo1f | 17 | 2.98 | 1.53E-08 | 3.56E-06 |
| Gal | 19 | 3.21 | 1.83E-08 | 4.20E-06 |
| Sirpb1c | 3 | 11.29 | 2.00E-08 | 4.47E-06 |
| Fgr | 4 | 3.79 | 3.49E-08 | 7.70E-06 |
| Fcer1g | 1 | 2.21 | 3.65E-08 | 7.95E-06 |
| Ifi209 | 1 | 3.37 | 7.32E-08 | 1.48E-05 |
| Cytip | 2 | 9.67 | 7.63E-08 | 1.53E-05 |
| Lcp1 | 14 | 2.02 | 1.64E-07 | 3.11E-05 |
| Fcgr1 | 3 | 3.02 | 2.08E-07 | 3.78E-05 |
| Itgb2 | 10 | 2.21 | 2.84E-07 | 4.94E-05 |
| Ccl2 | 11 | 7.68 | 3.63E-07 | 5.99E-05 |
| Fcgr2b | 1 | 2.33 | 3.85E-07 | 6.27E-05 |
| Csf3r | 4 | 3.21 | 3.93E-07 | 6.35E-05 |
| Bpifb9b | 2 | 5.91 | 4.04E-07 | 6.46E-05 |
| Serpine1 | 5 | 3.84 | 4.38E-07 | 6.94E-05 |
| Tyrobp | 7 | 2.37 | 4.84E-07 | 7.61E-05 |
| Pmel | 10 | -3.84 | 5.54E-07 | 8.62E-05 |
| Ifi211 | 1 | 4.29 | 5.66E-07 | 8.66E-05 |
| Wdfy4 | 14 | 2.59 | 5.66E-07 | 8.66E-05 |
| Gvin1 | 7 | 2.08 | 7.01E-07 | 1.04E-04 |
| Slamf7 | 1 | 9.64 | 7.70E-07 | 1.14E-04 |
| Ms4a6c | 19 | 2.89 | 7.89E-07 | 1.16E-04 |
| Prss56 | 1 | 9.64 | 8.49E-07 | 1.23E-04 |
| Slfn1 | 11 | 13.81 | 9.43E-07 | 1.35E-04 |
| Cyp4f18 | 8 | 3.96 | 9.89E-07 | 1.38E-04 |
| Adamts19 | 18 | 4.48 | 1.05E-06 | 1.44E-04 |
| Atp8b4 | 2 | 18.2 | 1.08E-06 | 1.47E-04 |
| Gm20431 | 2 | 8.18 | 1.10E-06 | 1.48E-04 |
| Ncf1 | 5 | 2.02 | 1.10E-06 | 1.48E-04 |
| Ms4a6d | 19 | 4.19 | 1.19E-06 | 1.59E-04 |
| Ms4a6b | 19 | 3.06 | 1.26E-06 | 1.67E-04 |
| Cd48 | 1 | 3.88 | 1.40E-06 | 1.84E-04 |
| Egr3 | 14 | 2.25 | 1.71E-06 | 2.19E-04 |
| Clec4n | 6 | 8.21 | 1.73E-06 | 2.20E-04 |
| Ifi207 | 1 | 4 | 1.87E-06 | 2.36E-04 |
| Cd84 | 1 | 2.27 | 1.93E-06 | 2.42E-04 |
| Trim30a | 7 | 2.15 | 1.96E-06 | 2.43E-04 |
| Cd300ld | 11 | 6.33 | 2.07E-06 | 2.56E-04 |
| Cxcr2 | 1 | 21.81 | 2.19E-06 | 2.67E-04 |
| Tlr13 | X | 2.5 | 2.84E-06 | 3.32E-04 |

| | | | | |
|---|---|---|---|---|
| Ptafr | 4 | 2.82 | 3.78E-06 | 4.31E-04 |
| Cebpa | 7 | 2.18 | 3.81E-06 | 4.32E-04 |
| Cd53 | 3 | 2.05 | 4.07E-06 | 4.57E-04 |
| Nfam1 | 15 | 2.37 | 5.14E-06 | 5.59E-04 |
| Mlana | 19 | -2.7 | 5.28E-06 | 5.65E-04 |
| Ikzf1 | 11 | 2.39 | 5.31E-06 | 5.65E-04 |
| Mndal | 1 | 3.15 | 5.78E-06 | 6.07E-04 |
| H2-DMb1 | 17 | 2.8 | 5.95E-06 | 6.10E-04 |
| Arhgap30 | 1 | 2.28 | 5.98E-06 | 6.10E-04 |
| Ccl5 | 11 | 5.05 | 5.98E-06 | 6.10E-04 |
| Ccr5 | 9 | 2.12 | 7.15E-06 | 7.16E-04 |
| Ctla2a | 13 | 2.48 | 8.12E-06 | 7.92E-04 |
| Gpr141 | 13 | 10.73 | 8.13E-06 | 7.92E-04 |
| Tlr2 | 3 | 2.63 | 9.29E-06 | 8.93E-04 |
| Trim30d | 7 | 2.16 | 1.00E-05 | 9.56E-04 |
| Pmaip1 | 18 | 2.97 | 1.19E-05 | 1.12E-03 |
| Themis2 | 4 | 2.73 | 1.27E-05 | 1.18E-03 |
| H2-Q7 | 17 | 2.51 | 1.44E-05 | 1.33E-03 |
| Spi1 | 2 | 2.71 | 1.52E-05 | 1.38E-03 |
| Ptpn6 | 6 | 2.32 | 1.64E-05 | 1.46E-03 |
| Ccr1 | 9 | 3.13 | 1.78E-05 | 1.57E-03 |
| Bcl2a1b | 9 | 3.61 | 1.80E-05 | 1.58E-03 |
| Sla | 15 | 2.31 | 2.05E-05 | 1.73E-03 |
| Ifi203 | 1 | 2.63 | 2.06E-05 | 1.74E-03 |
| Clec4a3 | 6 | 6.02 | 2.12E-05 | 1.76E-03 |
| F10 | 8 | 4.97 | 2.12E-05 | 1.76E-03 |
| Gm49339 | 10 | 3.34 | 2.30E-05 | 1.89E-03 |
| Clec4a1 | 6 | 4.02 | 2.44E-05 | 1.97E-03 |
| Arg1 | 10 | 2.34 | 2.56E-05 | 2.05E-03 |
| Cd300lb | 11 | 9.78 | 2.93E-05 | 2.31E-03 |
| Icam1 | 9 | 2.43 | 3.05E-05 | 2.39E-03 |
| Cxcl10 | 5 | 4.05 | 3.10E-05 | 2.43E-03 |
| Apoc2 | 7 | 6.08 | 3.22E-05 | 2.47E-03 |
| Ifi213 | 1 | 7.17 | 3.25E-05 | 2.47E-03 |
| Csf2rb | 15 | 2.63 | 3.30E-05 | 2.50E-03 |
| Acp5 | 9 | 6.88 | 3.39E-05 | 2.55E-03 |
| Gpr65 | 12 | 3.68 | 3.47E-05 | 2.58E-03 |
| Napsa | 7 | 4.22 | 3.48E-05 | 2.58E-03 |
| Cd52 | 4 | 2.43 | 3.69E-05 | 2.72E-03 |
| Runx3 | 4 | 4.77 | 4.11E-05 | 3.00E-03 |

| | | | | |
|---|---|---|---|---|
| Il6ra | 3 | 2.11 | 4.39E-05 | 3.15E-03 |
| Neurl3 | 1 | 2.3 | 4.54E-05 | 3.20E-03 |
| Fyb | 15 | 2.25 | 4.61E-05 | 3.24E-03 |
| Fosl1 | 19 | 3.04 | 5.59E-05 | 3.82E-03 |
| Lcp2 | 11 | 2.07 | 6.23E-05 | 4.21E-03 |
| Bpifb9a | 2 | 8.37 | 6.89E-05 | 4.60E-03 |
| Cd209a | 8 | 11.14 | 6.95E-05 | 4.63E-03 |
| Cmklr1 | 5 | 2.07 | 7.53E-05 | 4.96E-03 |
| C5ar1 | 7 | 2.3 | 7.63E-05 | 4.98E-03 |
| Ly6c2 | 15 | 10.84 | 8.30E-05 | 5.30E-03 |
| Il10ra | 9 | 2.28 | 8.67E-05 | 5.47E-03 |
| Epsti1 | 14 | 2.81 | 9.47E-05 | 5.89E-03 |
| Oas1a | 5 | 2.77 | 9.74E-05 | 6.04E-03 |
| Steap4 | 5 | 2.63 | 9.80E-05 | 6.04E-03 |
| Cd300c2 | 11 | 2.53 | 1.15E-04 | 6.96E-03 |
| Tyrp1 | 4 | -2.4 | 1.21E-04 | 7.23E-03 |
| Tlr8 | X | 5.11 | 1.42E-04 | 8.27E-03 |
| Rac2 | 15 | 2.09 | 1.45E-04 | 8.41E-03 |
| Ms4a4c | 19 | 4.89 | 1.47E-04 | 8.45E-03 |
| Apobec1 | 6 | 2.06 | 1.51E-04 | 8.60E-03 |
| Csf2rb2 | 15 | 2.25 | 1.69E-04 | 9.41E-03 |
| Ror2 | 13 | 2.1 | 1.70E-04 | 9.41E-03 |
| Glycam1 | 15 | -3.51 | 1.74E-04 | 9.55E-03 |
| Crybb2 | 5 | 9.5 | 1.77E-04 | 9.67E-03 |
| Arhgap25 | 6 | 2.67 | 2.05E-04 | 0.01 |
| Hck | 2 | 2.14 | 2.23E-04 | 0.01 |
| Pira2 | 7 | 5.36 | 2.28E-04 | 0.01 |
| Spn | 7 | 6.88 | 2.28E-04 | 0.01 |
| Ms4a7 | 19 | 2.63 | 2.32E-04 | 0.01 |
| Baz1a | 12 | 2.01 | 2.41E-04 | 0.01 |
| Dct | 14 | -2.46 | 2.42E-04 | 0.01 |
| Gm21188 | 13 | 7.85 | 2.48E-04 | 0.01 |
| Kcnk6 | 7 | 2.13 | 2.66E-04 | 0.01 |
| Anpep | 7 | 2.67 | 2.79E-04 | 0.01 |
| Birc3 | 9 | 2.23 | 2.89E-04 | 0.01 |
| Dcn | 10 | -2.01 | 3.02E-04 | 0.02 |
| Cebpd | 16 | 2.46 | 3.15E-04 | 0.02 |
| B430306N03Rik | 17 | 4.22 | 3.49E-04 | 0.02 |
| Ncf4 | 15 | 3.9 | 3.53E-04 | 0.02 |
| Vav1 | 17 | 2.02 | 3.74E-04 | 0.02 |

| | | | | |
|---|---|---|---|---|
| P2ry6 | 7 | 2.14 | 3.81E-04 | 0.02 |
| Capg | 6 | 2.07 | 3.83E-04 | 0.02 |
| Slamf9 | 1 | 2.5 | 3.97E-04 | 0.02 |
| Clec5a | 6 | 2.51 | 3.98E-04 | 0.02 |
| Crygs | 16 | 8.02 | 4.09E-04 | 0.02 |
| Krt8 | 15 | -2.62 | 4.13E-04 | 0.02 |
| Rsad2 | 12 | 2.49 | 4.21E-04 | 0.02 |
| Mx1 | 16 | 5.01 | 4.32E-04 | 0.02 |
| Sh3rf2 | 18 | -3.05 | 4.35E-04 | 0.02 |
| Slc15a3 | 19 | 2.25 | 4.73E-04 | 0.02 |
| Phf11b | 14 | 3.11 | 5.00E-04 | 0.02 |
| Slfn8 | 11 | 2.17 | 5.30E-04 | 0.02 |
| Aoah | 13 | 3.45 | 5.34E-04 | 0.02 |
| Hsd17b2 | 8 | 2.13 | 5.85E-04 | 0.03 |
| Dpep2_1 | 8 | 4.44 | 5.93E-04 | 0.03 |
| Ly9 | 1 | 2.74 | 6.38E-04 | 0.03 |
| Cysltr1 | X | 2.49 | 6.72E-04 | 0.03 |
| Cryba1 | 11 | 4.25 | 6.73E-04 | 0.03 |
| H2ac19 | 3 | 3.2 | 6.86E-04 | 0.03 |
| Pgf | 12 | 2.54 | 6.99E-04 | 0.03 |
| H2bc24 | 13 | 2.42 | 7.87E-04 | 0.03 |
| Gm21985 | 2 | 2.57 | 7.92E-04 | 0.03 |
| Bcl2a1d | 9 | 3.93 | 8.08E-04 | 0.03 |
| Fcgr4 | 1 | 2.82 | 8.77E-04 | 0.03 |
| Ifi204 | 1 | 2.07 | 9.40E-04 | 0.04 |
| Card11 | 5 | 2.02 | 9.66E-04 | 0.04 |
| Was | X | 2.46 | 9.67E-04 | 0.04 |
| Treml2 | 17 | 2.7 | 9.95E-04 | 0.04 |
| Ccl22 | 8 | 5.47 | 9.97E-04 | 0.04 |
| Gm28539 | 16 | -3.5 | 1.01E-03 | 0.04 |
| Tmem140 | 6 | 2.41 | 1.08E-03 | 0.04 |
| Depdc7 | 2 | 2.59 | 1.22E-03 | 0.04 |
| Igf2bp1 | 11 | -2.2 | 1.25E-03 | 0.04 |
| Cd244a | 1 | 3.12 | 1.26E-03 | 0.04 |
